# Analysis of shared ceRNA networks and related-hub genes in rats with primary and secondary photoreceptor degeneration

**DOI:** 10.3389/fnins.2023.1259622

**Published:** 2023-09-21

**Authors:** Jia Liang, Dong Fang, Fei Yao, Lu Chen, Zhenhua Zou, Xiangcheng Tang, Lujia Feng, Yijing Zhuang, Ting Xie, Pengxue Wei, Pengfeng Li, Huiyan Zheng, Shaochong Zhang

**Affiliations:** Shenzhen Eye Hospital, Shenzhen Eye Institute, Jinan University, Shenzhen, Guangdong, China

**Keywords:** lncRNA-miRNA-mRNA networks, bioinformatics analysis, molecular mechanisms, photoreceptor degeneration, immune regulation

## Abstract

**Introduction:**

Photoreceptor degenerative diseases are characterized by the progressive death of photoreceptor cells, resulting in irreversible visual impairment. However, the role of competing endogenous RNA (ceRNA) in photoreceptor degeneration is unclear. We aimed to explore the shared ceRNA regulation network and potential molecular mechanisms between primary and secondary photoreceptor degenerations.

**Methods:**

We established animal models for both types of photoreceptor degenerations and conducted retina RNA sequencing to identify shared differentially expressed long non-coding RNAs (lncRNAs), microRNAs (miRNAs), and messenger RNAs (mRNAs). Using ceRNA regulatory principles, we constructed a shared ceRNA network and performed function enrichment and protein–protein interaction (PPI) analyses to identify hub genes and key pathways. Immune cell infiltration and drug–gene interaction analyses were conducted, and hub gene expression was validated by quantitative real-time polymerase chain reaction (qRT-PCR).

**Results:**

We identified 37 shared differentially expressed lncRNAs, 34 miRNAs, and 247 mRNAs and constructed a ceRNA network consisting of 3 lncRNAs, 5 miRNAs, and 109 mRNAs. Furthermore, we examined 109 common differentially expressed genes (DEGs) through functional annotation, PPI analysis, and regulatory network analysis. We discovered that these diseases shared the complement and coagulation cascades pathway. Eight hub genes were identified and enriched in the immune system process. Immune infiltration analysis revealed increased T cells and decreased B cells in both photoreceptor degenerations. The expression of hub genes was closely associated with the quantities of immune cell types. Additionally, we identified 7 immune therapeutical drugs that target the hub genes.

**Discussion:**

Our findings provide new insights and directions for understanding the common mechanisms underlying the development of photoreceptor degeneration. The hub genes and related ceRNA networks we identified may offer new perspectives for elucidating the mechanisms and hold promise for the development of innovative treatment strategies.

## Introduction

The photoreceptors in the retina, including rods and cones, can convert light into brain-recognizable electrical signals transmitted to the brain (Kawamura and Tachibanaki, 2022). However, degenerative diseases such as age-related macular degeneration and retinitis pigmentosa can lead to photoreceptor damage or degeneration, resulting in blindness (Newton and Megaw, 2020). Unfortunately, the natural repair mechanisms of photoreceptors are limited, making the degenerative process irreversible. Photoreceptor degeneration can be classified into two types: primary and secondary. Primary photoreceptor degeneration is often associated with genetic mutations, such as the Mertk gene mutation (Evans et al., 2017; Lew et al., 2020), while secondary photoreceptor degeneration can be induced by chemical agents or ultraviolet stimulation. N-methyl-N-nitrosourea (MNU) is a common drug used to induce secondary photoreceptor degeneration (Deng et al., 2021). Despite previous studies suggesting that activated microglia, inflammation, autophagy, and oxidative stress contribute to photoreceptor degeneration (Newton and Megaw, 2020; Rashid et al., 2020), the common underlying pathogenesis of primary and secondary photoreceptor degeneration remains unclear. Understanding the shared pathogenesis and identifying therapeutic targets for both types of photoreceptor degeneration would be of great clinical value for patients.

In recent years, microarray and RNA-seq data analysis have emerged as powerful tools in disease research, aiding diagnosis and treatment strategies (Zhou et al., 2018). The competing endogenous RNA (ceRNA) hypothesis (Salmena et al., 2011) proposes that various types of RNAs, including long non-coding RNAs (lncRNAs), microRNAs (miRNAs), and messenger RNAs (mRNAs), form a complex regulatory network that controls gene expression at the post-transcriptional level. Specifically, cytoplasmic lncRNAs indirectly regulate mRNA expression by competitively binding miRNAs through reciprocally complementary seed region-shared miRNA response elements. This interaction ultimately leads to up-regulation of the corresponding mRNAs by influencing their stability and translational regulation (Zhang et al., 2019). Increasing evidence has demonstrated the involvement of ceRNA-mediated regulation in the pathogenesis and progression of various diseases, including coronary heart disease (Li H. et al., 2021), hepatocellular carcinoma (Bai et al., 2019) and retinal ischemia-reperfusion injury (Zhang et al., 2021). However, the expression patterns and underlying mechanisms of specific ceRNA networks in primary and secondary photoreceptor degenerative diseases remain unclear.

Therefore, in this study, we conducted whole-transcriptome sequencing of the retina in RCS rats (a primary photoreceptor degeneration model) and RDY rats treated with MNU (a secondary photoreceptor degeneration model) to investigate the shared ceRNA network. First, we compared the differentially expressed lncRNAs, miRNAs, and mRNAs between the retinas of primary and secondary photoreceptor degeneration models and normal tissues. Subsequently, we identified 3 lncRNAs, 5 miRNAs, and 109 mRNAs as candidates for constructing a shared regulatory network of ceRNAs. Next, we identified hub genes through protein–protein interaction (PPI) analysis and reconstructed the ceRNA network associated with these hub genes. The reconstructed network, including 2 lncRNAs, 2 miRNAs and 8 mRNAs, was validated using reverse transcription-quantitative polymerase chain reaction (RT-qPCR). Finally, we performed functional enrichment analysis, functional annotation, and drug–gene analysis of the hub genes, aiming to reveal the underlying shared mechanisms and potential therapeutic drugs of primary and secondary photoreceptor degeneration.

## Materials and methods

### Animals

Royal College of Surgeons (RCS) rats have pathologic rdy- RCS rats (RCS, male, 180∼ 200 g, 8 weeks old) and normal rdy + RCS rats (RDY, male, 180∼ 200 g, 8 weeks old) were obtained from the Xian Provincial Experimental Animal Research Center (Xian, China). Rats were housed comfortably under a 12-h light/dark cycle. All animal experiments complied with the National Institute of Health guidelines and were approved by the ethics committee of Shenzhen Eye Hospital, Jinan University (Guangdong, China). RDY rats were considered as a control group. RCS rats were considered for primary photoreceptor degeneration. We randomly selected 96 RDY rats and equally divided them into 4 groups. The rats were, respectively, performed intraperitoneally injected with different concentrations of MNU (0, 40, 60, 100 mg/kg) in each group at the same time. We sampled and observed the retinal outer nuclear layer (ONL) thickness and cell numbers at 0, 1, 3, 7 days after MNU treatment. We observed that the ONL thickness and cell numbers decreased by 50% within 1 day when using 60 mg/kg of MNU, which was considered ideal injury condition. Therefore, models of secondary photoreceptor degeneration were established by single intraperitoneal injections of 60 mg/kg MNU in RDY rats for 1 day. Animals were randomly assigned to three groups: control group (healthy RDY rats; *n* = 40), RCS group (*n* = 40) and MNU group (RDY rats were intraperitoneally injected with 60 mg/kg MNU for 1 day, *n* = 40).

### Retinal histology and morphology

The rats were examined after MNU treatment with various doses (0, 40, 60, 100 mg/kg) and their eyeballs were extracted after a humane sacrifice and examined histologically at different times (0, 1, 3, 7 days) after MNU treatment. The RCS rats and RDY rats (8 weeks old) were sampled. The retinal tissues were rapidly stripped, fixed overnight in 4% paraformaldehyde, embedded in paraffin, and cut into 5-m serial slices (RM2125; Leica), after which the retina morphology and histology were determined by hematoxylin-eosin (HE) staining (Hu et al., 2018). The thickness of the ONL across the optic nerve head (ONH) and along the vertical meridian of the eyeball from nasal to temporal was measured using Fiji ImageJ 2.3.0 software (Stossi and Singh, 2023).

### Optical coherence tomography (OCT)

To assess retinal structures, OCT was used with a Retinal Imaging Microscope (Micron IV; Phoenix Research Lab, Pleasanton, CA, USA). In addition, the retinal ONL thickness was examined using Fiji ImageJ 2.3.0 software.

### Transcriptome analysis

The retinas of RDY rats were obtained after MNU treatment with 60 mg/kg for 1 day. The retina of rats (8 weeks old) in the other two groups were obtained at the same time. Four individual retinas were pooled as one sample, and three samples were analyzed per group. RNA sequencing was performed using the DNBSEQ platform (BGI) (Huada Gene Technologies Ltd., Guangdong, China). The data were analyzed on the Dr. Tom II network platform of the BGI.^1^

### Differentially expressed gene analysis

FASTQC, a Java-based quality control tool, was used for FASTQ data quality control (QC) and then SOAPnuke (v1.5.6) was used to filter the sequencing data (raw data) (Li et al., 2008). The clean data were then used to map to the reference genome using HISAT2 (v2.1.0) (Kim D. et al., 2015). Finally, Bowtie2 (v2.2.5) was used to align the clean data to the reference gene set and RSEM (v1.2.8) was used to determine the expression level of the genes and draw heatmap by Pheatmap (v1.0.8) (Li and Dewey, 2011). Besides, genes with a Q value lower than 0.05 and a fold-change ≥ 1.5 regarded as differentially expressed, were screened and analyzed using DESeq2 (v1.4.5) (Love et al., 2014). All the procedures above procedures were performed by the Dr. Tom II network platform of the BGI (see text footnote 1) (Langmead and Salzberg, 2012).

### Functional enrichment analysis

Gene ontology (GO) enrichment of co-DEmRNAs was evaluated and visualized by the Dr. Tom II network platform of the BGI. The Kyoto Encyclopedia of Genes and Genomes (KEGG) pathway enrichment analysis was assessed by the Metascape and visualized by the bioinformatics analysis platform.^2^ The Q value < 0.05 was significant for the enrichment threshold.

### Construction of lncRNA-miRNA-mRNA network

The target miRNA of lncRNAs and target mRNA of miRNAs were predicted from the TargetScan (Li H. et al., 2021), RNAhybrid (Krüger and Rehmsmeier, 2006) and miRandam (Zhu et al., 2019) databases via the Dr. Tom II network platform of the BGI (see text footnote 1). Besides, because lncRNAs can function as nodes of the ceRNA network, mainly in the cytoplasm, we investigated the intracellular localization of the lncRNAs via the lncATLAS database (Mas-Ponte et al., 2017). Finally, ceRNA networks based on interactions between cytoplasmic DElncRNAs and DEmiRNAs and between DEmiRNAs and DEmRNAs were constructed and visualized using Cytoscape software (version 3.9.1) (Shannon et al., 2003).

### Protein–protein interaction network and hub gene identification

A PPI network of co-DEGs was constructed by the STRING online database (Szklarczyk et al., 2021) and was visualized using Cytoscape (version 3.9.1). The degree computation of MCODE plugin in Cytoscape was used to identify hub genes, which are thought to exert crucial functions in the network. The hub genes were selected from the top 8 ranking degree scores by cytoHubba.

### Analyses of the ceRNA network-related hub genes

Gene ontology (GO) annotation and visualization of the hub genes were performed by the Metascape database (Zhou et al., 2019). Next, the hub genes were analyzed by the ImmQuant software (Frishberg et al., 2016), which provides as output the predicted quantities of various immune-cell types between each sample based on marker gene-expression data using deconvolution methodology. Besides, according to the analysis results of immune infiltration, we evaluated the correlation between the expression of 8 hub genes and the abundance of immune cell infiltration in three groups and made a lollipop chart in the bioinformatics analysis platform (see text footnote 2). The drug–gene interaction analysis of hub genes was evaluated by the DGIDB database^3^ and visualized by Cytoscape (version 3.9.1). Finally, the ceRNA network related to the hub genes was reconstructed and visualized with an alluvial chart in the bioinformatics analysis platform (see text footnote 2).

### RT-qPCR validation analysis

Total RNA was extracted from retina samples using an EastepTM Super Total RNA Extraction Kit (Cat. No. LS1040, Promega Co., Madison, USA) and was reverse transcribed into cDNA with a reverse transcription kit [PrimeScript RT reagent kit (Cat. No. RR600A, Takara Bio, Inc., Tokyo, Japan)] or miDETECT A TrackTM miRNA qRT-PCR Starter Kit (RiboBio Co., Guangzhou, China) and gDNA Remover. Subsequently, real-time PCR was performed using the S6 Universal SYBR qPCR mix (NovaBio Co., Shanghai, China) on a Bio-Rad iCycler Real-time Quantitative PCR system (Bio-Rad, CA, United States). Relevant qPCR primers of the lncRNAs, miRNAs and hub genes were designed in Supplementary Table 1. The relative expression levels of the target genes were normalized to those of GAPDH and calculated using the 2-ΔΔCt method (Schmittgen and Livak, 2008).

### Statistical considerations

Data are normally distributed and presented as the median ± range (SD). Groups were compared using a one-way analysis of variance (ANOVA) followed by the Bonferroni *post-hoc* test in SPSS 29.0 (IBM, Armonk, NY, USA). Values of *P* < 0.05 were considered to be statistically significant.

## Results

### Primary and secondary photoreceptor degeneration model was constructed *in vivo*

**FIGURE 1:**
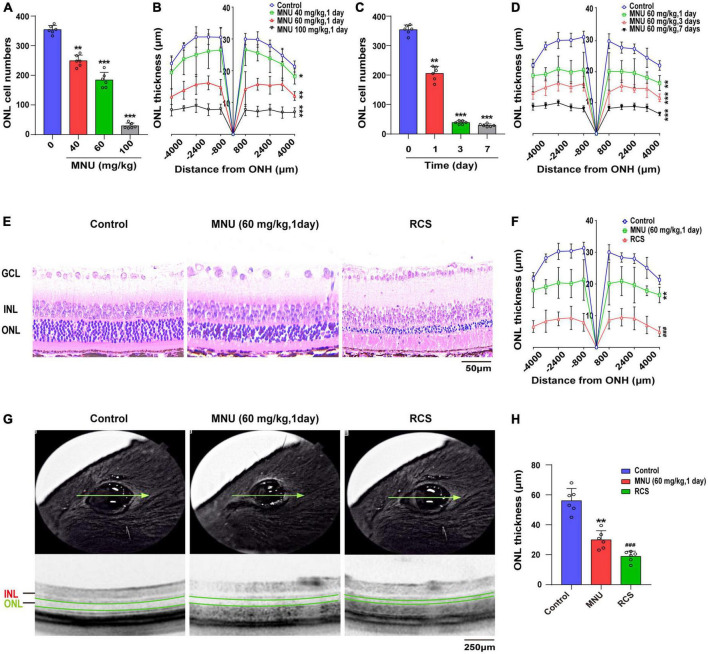
Primary and secondary photoreceptor degeneration model was constructed. **(A)** RDY rats were subjected to MNU injection at various doses for 1 day and the cell numbers of ONL were decreased, and **(B)** the thickness of the ONL was also reduced (*n* = 6). **(C)** The RDY rats treated with 60 mg/kg MNU at different times and the retinal ONL cell number was reduced, and **(D)** the ONL thickness decreased over time (*n* = 6). **(E)** Representative images of HE-stained retinal tissues in RDY rats, RDY rats treated with 60 mg/kg MNU for 1 day and RCS rats at 8 weeks old and **(F)** quantification of the thickness of the ONL (*n* = 6). **(G)** Representative retinal tissue images of OCT in RDY rats, RDY rats treated with 60 mg/kg MNU for 1 day and RCS rats at 8 weeks old and **(H)** quantification of the thickness of the ONL (*n* = 6). RDY, normal rdy + RCS rats; MNU, N-methyl-N-nitrosourea; RCS, Royal College of Surgeons rats; INL, inner nuclear layer; ONL, outer nuclear layer; ONH, optic nerve head; GCL, ganglion cell layer; OCT, optical coherence tomography. Scale bars = 50 μm **(E)**. 250 μm **(G)**. *P ≤ 0.05, **P ≤ 0.01 and ***P ≤ 0.001 according to the ANOVA test (MNU-treatment vs. the control). ^###^*P* ≤ 0.001 according to the ANOVA test (the RCS vs. the control).

N-methyl-N-nitrosourea (MNU) is a DNA alkylating agent commonly used to induce secondary photoreceptor degeneration in RDY rats. In MNU-treated rats, retinal ONL cell number and thickness decrease in a time- and dose-dependent manner (Figures 1A–D). RDY rats are intraperitoneally injected with 60 mg/kg MNU for 1 day as an animal model for photoreceptor denaturation. Moreover, respective HE staining and OCT images are shown in Figures 1E–H. The RCS rats are a naturally mutated photoreceptor degeneration model because of the Mertk (-/-) mutant, which alters the functions of retinal pigmented epithelium cells involved in binding desquamating plaque protein products. The photoreceptors of RCS rats start lost at 18 days and completely disappear after 3 months. Therefore, we selected RCS rats at 8 weeks old as the primary photoreceptor degeneration model. The decreased retina thickness and cell numbers of the ONL were validated by OCT and HE staining (Figures 1E–H).

### Aberrantly expressed lncRNAs, miRNAs, and mRNAs

**FIGURE 2:**
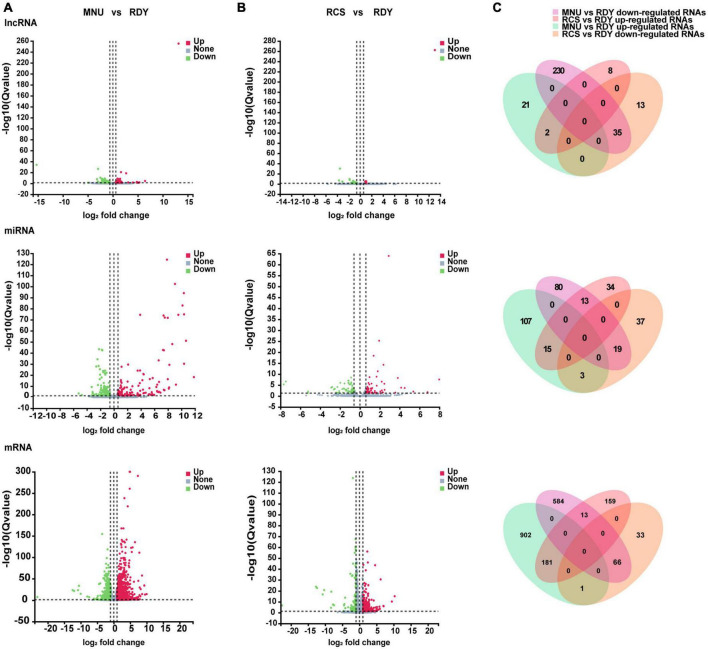
Volcano plots of differentially expressed lncRNAs, miRNAs and mRNAs. Volcano plots of the differentially expressed lncRNAs (above plot), miRNAs (medium plot), and mRNAs (below plot) in the comparison between MNU group versus RDY group **(A)** and RCS group versus RDY group **(B)**. Upregulated genes marked red and downregulated genes marked green. **(C)** Venn diagrams show the intersections of upregulated and downregulated co-DElncRNAs (above plot), co-DEmiRNAs (medium plot), and co-mRNAs (below plot) both in MNU group versus RDY group and RCS group versus RDY group. The purple and green areas represent downregulated and upregulated RNAs in the MNU group versus the RDY group. The orange and pink areas represent downregulated and upregulated RNAs in the RCS versus the RDY group. DEG, differentially expressed gene; lncRNA, long non-coding RNA; miRNA, microRNA; mRNA, messenger RNA.

Retinal full-length transcriptome sequencing was performed between primary and secondary photoreceptor degeneration and normal rats. To better understand the disease-associated differentially expressed genes (DEGs), we first classified the entire sample into RDY, RCS, and MNU-treated retinal groups. Firstly, we compared differential lncRNAs, miRNAs (fold change ≥ 1.5, Q value < 0.05) and mRNAs (fold change ≥ 2, Q value < 0.05) both in the RCS group and MNU-treated group, respectively, with those of the RDY group, as shown in the volcano map (Figures 2A, B). The three groups of DEG cross-points consist of 37 lncRNA (2 up-regulated, 35 down-regulated), 34 miRNAs (15 up-regulated, 19 down-regulated), and 247 mRNA (181 up-regulated, 66 down-regulated), which are considered to be critical genes involved in photoreceptor degeneration (Figure 2C).

### Prediction of miRNAs targeted by lncRNAs and intracellular localization of lncRNAs

**FIGURE 3:**
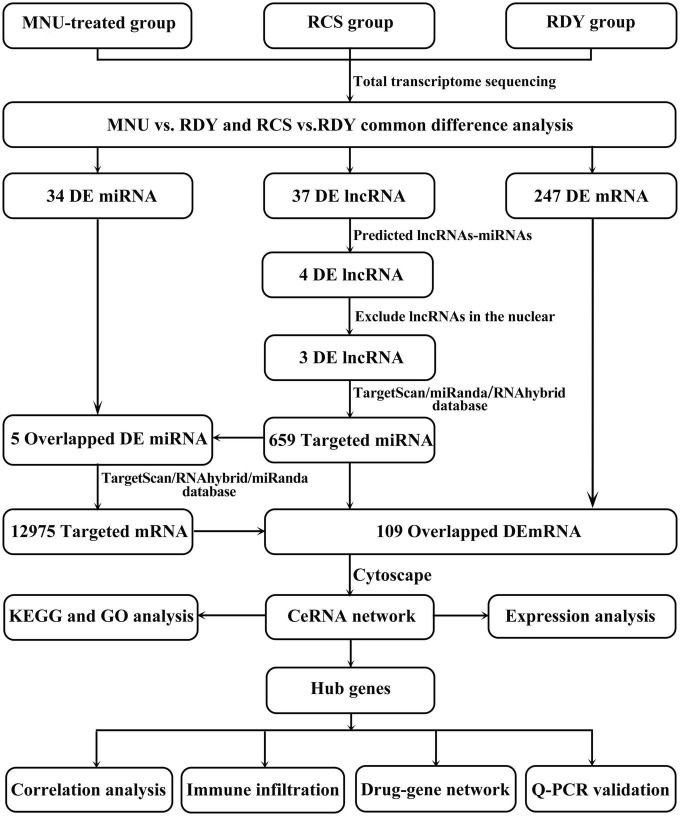
Flow chart of this study. RDY, normal rdy + RCS rats; MNU, N-methyl-N-nitrosourea; RCS, Royal College of Surgeons rats; ceRNA, competitive endogenous RNA; lncRNA, long non-coding RNA; miRNA, microRNA; mRNA, messenger RNA.

The process of creating a ceRNA network is shown in Figure 3. First, we identified 37 overlapped differentially expressed lncRNAs. Then, we predicted potential miRNA interactions with 37 lncRNA shared by the three databases (RNAhybrid/miRanda/Targetscan) through the Dr. Tom II network platform of the BGI. Next, crossover miRNAs between predicted miRNAs and 34 DEmiRNAs were obtained. We identified 4 lncRNAs and 6 interacting miRNAs (Supplementary Table 2). Since the intrinsic competition of lncRNAs is mainly in the cytoplasm, we excluded one of the 4 DElncRNAs in the nucleus through the lncLocator database. Finally, 3 lncRNAs and 5 DEmiRNAs were identified.

### Prediction of mRNAs targeted by miRNAs and construction of the ceRNA network

**FIGURE 4:**
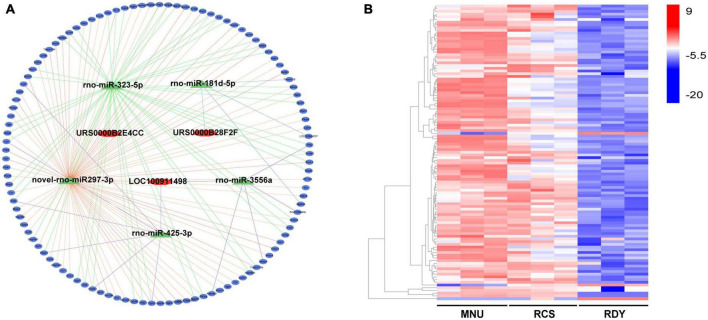
Construction of the ceRNA network and analysis of ceRNA-related DEG expression. **(A)** The ceRNA network was constructed based on 3 lncRNAs (represented by red rhomb), 5 miRNAs (represented by green triangles), and 109 DEGs (represented by blue ovals). **(B)** The expression levels of ceRNA-related DEGs were visualized in a heatmap, where red gene bars indicate up-regulated genes and blue bars represent downregulated genes. ceRNA, competitive endogenous RNA; DEGs, differentially expressed genes; lncRNA, long non-coding RNA; miRNA, microRNA; mRNA, messenger RNA.

Similarly, the target mRNA of the 5 miRNAs mentioned above was predicted by the Dr. Tom II network platform of the BGI. One hundred and nine overlapped mRNA were obtained between 12,975 candidate target mRNAs and 247 DEmRNAs (Supplementary Table 3). Finally, 3 DElncRNAs, 5 DEmiRNAs, and 109 DEmRNAs were integrated into a final ceRNA network visualized with Cytoscape (Figure 4A). Besides, 109 DEmRNAs include 106 up-regulated genes and 3 down-regulated genes, which were presented in heatmap by the biocloud analysis platform^4^ (Figure 4B).

### GO and KEGG enrichment analyses

**FIGURE 5:**
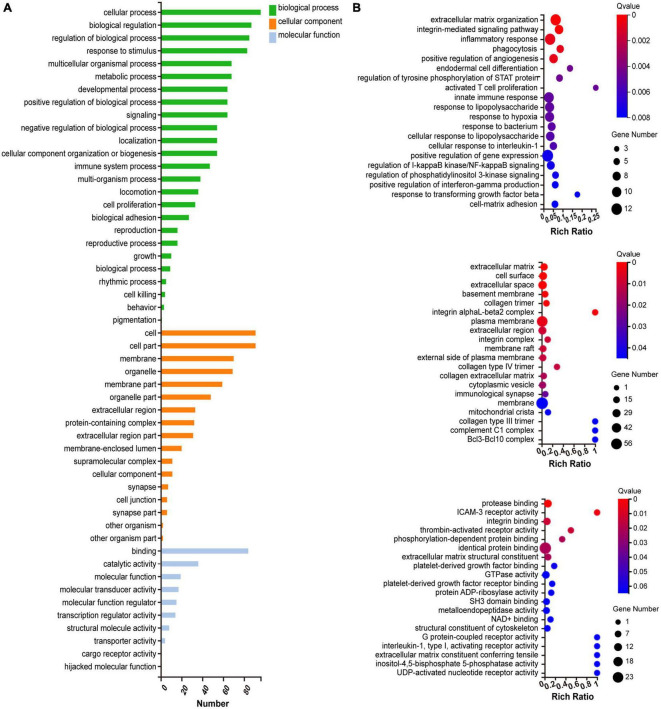
Gene ontology (GO) functional enrichment analysis of DEGs. **(A)** Classification of GO functional enrichment terms. **(B)** Bubble plots present DEGs’ top 20 significant biological processes, cellular components, and molecular functions. Each bubble with varied colors represents a different q-value. DEGs, differentially expressed genes.

**FIGURE 6:**
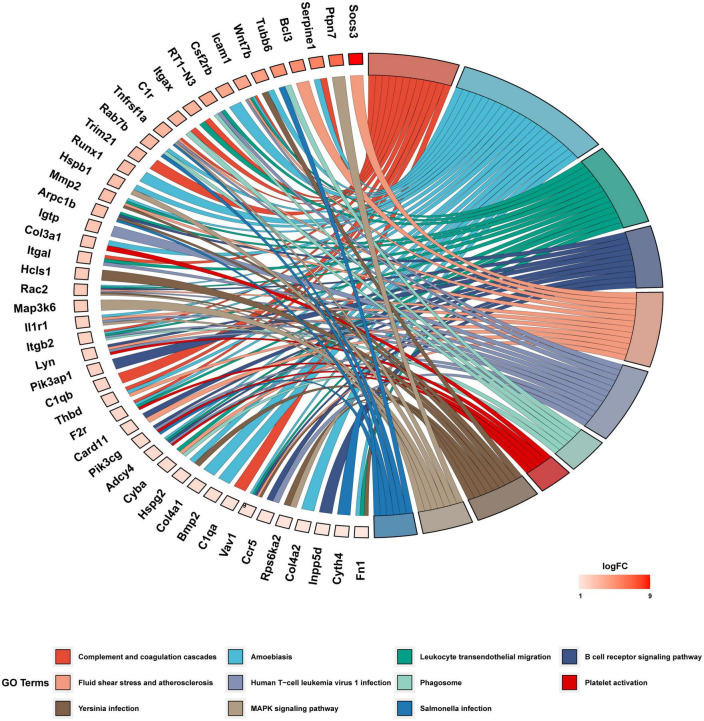
KEGG functional enrichment analysis of DEGs. The chord diagram displayed 11 significantly enriched GO terms of 109 DEmRNAs. The GO terms are defined as indicated color bars at the bottom and shown on the right of the chord diagram; the involved DEmRNAs are shown on the left. The red gene bars represent upregulated. CeRNA, competitive endogenous RNA; DEGs, differentially expressed genes; KEGG, Kyoto Encyclopedia of Genes and Genomes; lncRNA, long non-coding RNA; miRNA, microRNA; mRNA, messenger RNA.

We investigated the underlying biological function and pathways of 109 DEGs in the newly formed ceRNA networks. Using the Dr. Tom II network platform of the BGI, we performed a GO functional enrichment analysis (including biological processes BP, cellular components CC, and molecular functions MF) and identified 24 significant GO terms in BP, 17 GO terms in CC and 8 GO terms in MF (*p* < 0.01; Figure 5A). The three parts of GO enrichment results are presented in the bubble graph. Besides, among all GO terms, the top 5 BPs were “extracellular matrix organization,” “integrin-mediated signaling pathway,” “inflammatory response,” “phagocytosis,” and “positive regulation of angiogenesis”; the top 5 CCs were “extracellular matrix,” “cell surface,” “extracellular space,” “basement membrane,” and “collagen trimer”; the top 5 MFs were “protease binding,” “ICAM-3 receptor activity”, “integrin binding,” “thrombin-activated receptor activity,” and “phosphorylation-dependent protein binding,” which ranked in ascent order of *p*-values (all *p* < 0.01) (Figure 5B). Subsequently, the KEGG pathway enrichments of 109 DEGs were performed by the Metascape database. The top 11 KEGG clusters with their representative enriched terms were found, with the most significant pathway being complement and coagulation cascades (Supplementary Table 4). The chord plot presented the relationship between the DEmRNA and the top 11 KEGG clusters with their representative enriched terms, visualized with the bioinformatics analysis platform (see text footnote 2) (Figure 6).

### The protein–protein interaction network and hub gene screening

**FIGURE 7:**
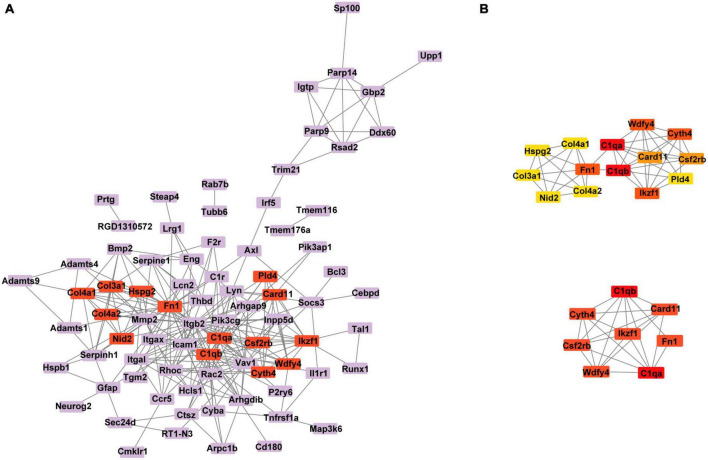
**(A)** Protein–protein interaction (PPI) network of the DEGs was constructed. **(B)** The network of the cluster with the highest degree score contained 14 genes (above plot) and the interaction network of the top 8 hub genes (below plot). DEGs, differentially expressed genes.

To further visualize the interaction between DEGs, we constructed a PPI network based on a STRING database. The network comprises 80 nodes and 263 edges visualized via the Cytoscape (Figure 7A). According to the node degree method, 4 clusters were identified. The cluster with the highest degree score contained 14 genes and 43 edges (Figure 7B). We selected the top 8 hub genes for further study, including Card11, Csf2rb, C1qb, C1qa, Ikzf1, Fn1, Wdfy4 and Cyth4 (Supplementary Table 5). In addition, interactions between the 8 hub genes were reconstructed by Cytoscpe (Figure 7B).

### Immune infiltration analysis of photoreceptor degeneration based on ceRNA-related hub genes

**FIGURE 8:**
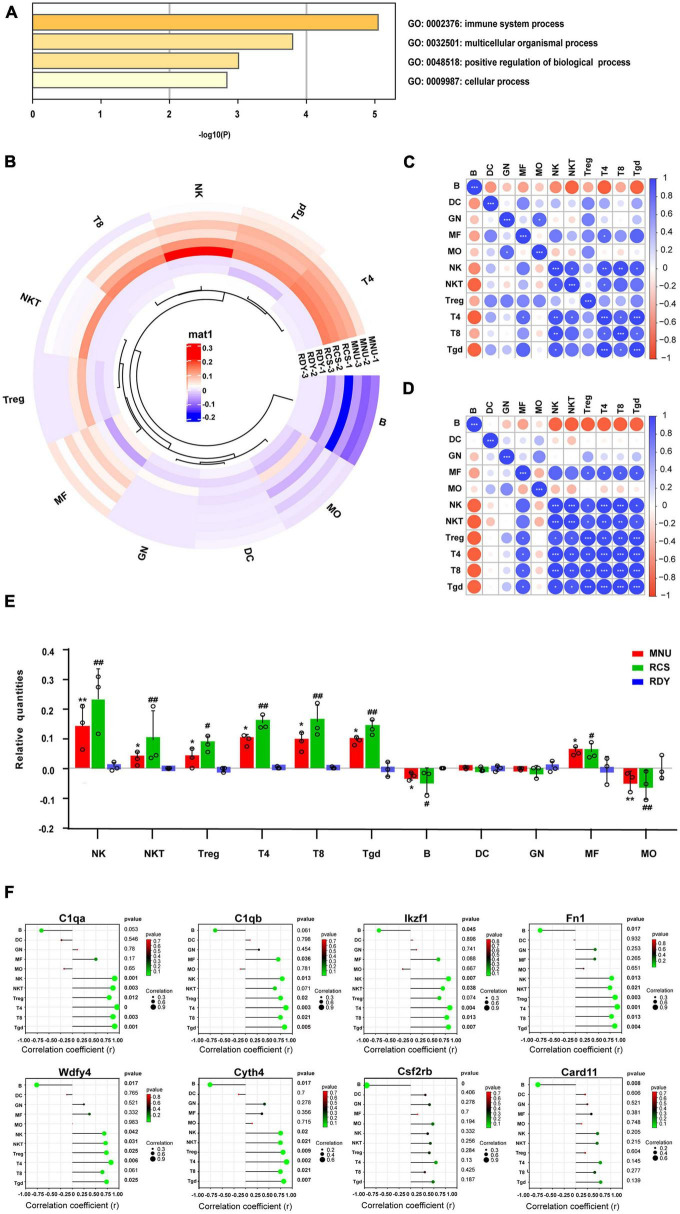
Immune infiltration analysis of ceRNA-related hub genes in two photoreceptor degenerations. **(A)** GO enrichment of ceRNA-related hub genes involving the immune system process. **(B)** Immune cell infiltrates analysis by Immquant software. The heatmap presents differences in cell-type quantities between the samples (*n* = 3). **(C)** The correlation analysis between each of the immune cells of primary photoreceptor degeneration and **(D)** secondary photoreceptor degeneration. **(E)** The relative quantities of immune cells between two photoreceptor degenerations and the control. CeRNA, competitive endogenous RNA; MNU, N-methyl-N-nitrosourea; RCS, Royal College of Surgeons rats; T4, CD4+ T cells; T8, CD8+ T cells; Tgd cells, gamma delta T cells; NK, natural killer cell. *P ≤ 0.05, **P ≤ 0.01 and ***P ≤ 0.001 according to the ANOVA test (MNU-treatment vs. the control). ^#^*P* ≤ 0.05 and ^##^*P* < 0.01 according to the ANOVA test (the RCS vs. the control). **(F)** The Spearman correlation analysis between hub genes and immune cell-type quantities. The size of the circle represents the correction coefficient. Bold font represents *p* < 0.05.

**FIGURE 9:**
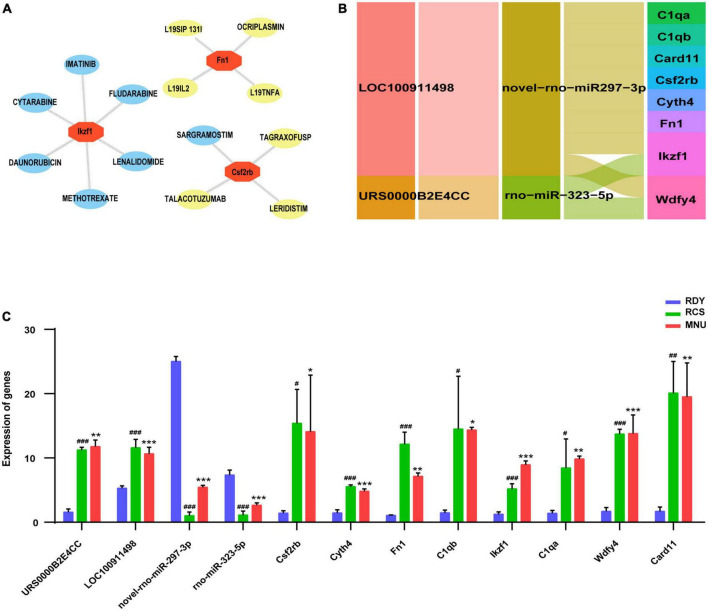
**(A)** Drug–gene interaction of hub genes. The immune therapeutical drugs were marked in yellow. **(B)** Construction of hub gene-related ceRNA network. **(C)** Validation of hub genes related-ceRNA, including 2 lncRNAs, 2 miRNAs and 8 mRNAs. Data are represented as means ± SEM and the experiments were repeated thrice. *P ≤ 0.05, **P ≤ 0.01 and ***P ≤ 0.001 according to the ANOVA test (MNU-treatment vs. the control). ^#^*P* ≤ 0.05, ^##^*P* < 0.01 and ^###^*P* ≤ 0.001 according to the ANOVA test (the RCS vs. the control).

Gene ontology (GO) enrichment analysis was performed on the 8 hub genes from the ceRNA network by Metascape. Eight biological processes with GO analysis were enriched and high enrichment was the immune system process (Figure 8A). Combined with the enrichment results of co-DEmRNA, we speculated that immune infiltration might participate in the occurrence of both primary and secondary photoreceptor degeneration. Thus, we evaluated the relative quantities of immune cells using the Immquant software and plotted a heatmap in Figure 8B. The correlation matrix showed that the strong correlation between Tgd cells and T4 cells was presented in primary photoreceptor degeneration and secondary photoreceptor degeneration (Figures 8C, D). In relative quantities analysis of immune cells, we found change fold values of T cell (including T4, T8 and Tgd cell) and NK cell were higher in both the MNU-treated and RCS groups than in the control group, but the change fold values of B cell and MO cell were lower (Figure 8E). Besides, the Spearman correlation between the hub gene and 11 type immune cell was evaluated, suggesting Csf2rb and Card11 have a strong correlation with relative quantities of B cells, and other hub genes have a strong correlation with relative quantities of T4 cell, T8 cell, NK cell and Tgd cell (Figure 8F). Finally, through drug–gene interaction analysis of the hub gene, we found 14 drug-targeting hub genes and 7 of those were immunotherapy drugs, including SARGRAMOSTIM, DAUNORUBICIN, LENALIDOMIDE, METHOTREXATE, CYTARABINE, IMATINIB, and FLUDARABINE (Figure 9A).

### Construction of key ceRNA network and validation of hub gene prediction and related ceRNA

Based on predictive 8 hub genes, key ceRNA networks, including 2 lncRNAs (LOC100911498 and URS0000B2E4CC) and 2 miRNAs (novel-rno-miR-297-3p and rno-miR-323-5p) were constructed and visualized by Cytoscape software (Figure 9B). Eight hub genes and 4 related ceRNAs were validated by RT-qPCR. Compared to the RDY control group, the expression of 8 hub genes and 2 lncRNAs were higher, but 2 miRNAs were lower in both the RCS and MNU-treated group (all *p* < 0.05). The RT-qPCR results were consistent with RNA-seq results indicating that the RNA-seq results are reliable and reproducible (Figure 9C).

## Discussion

Photoreceptor degeneration, including primary and secondary, leads to irreversible visual loss and even blindness without effective therapy (Gasparini et al., 2019). Several studies (Cehajic-Kapetanovic et al., 2020; Deng et al., 2021) reported that primary photoreceptor degeneration resulted from a gene mutation, whereas secondary photoreceptor degeneration was caused by chemical drugs or physical stimulation. Although the pathogenesis is distinguished from primary and secondary photoreceptor degeneration, the common mechanisms in both photoreceptor degeneration remain unclear. Therefore, searching crossed pathogenesis and therapy targets may exert a stronger effect on improving the treatment and prognosis of patients with photoreceptor degeneration. In the present study, primary and secondary photoreceptor degeneration animal models were established. Subsequently, we used high-throughput sequencing technologies and bioinformatics to screen common DElncRNAs, DEmiRNAs and DEmRNAs in photoreceptor degeneration and constructed a ceRNA network. We found that the ceRNA network was associated with the immune process in primary and secondary photoreceptor degeneration.

N-methyl-N-nitrosourea (MNU) damages DNA integrity via alkylation, considered a specific photoreceptor degeneration model (Deng et al., 2021). RDY rats treated with MNU and RCS rats displayed decreased ONL thickness and cell numbers, in agreement with previous studies (Kim K. A. et al., 2015; Koriyama and Furukawa, 2016). The result revealed that the same pathogenesis might exist in both photoreceptor degenerations. Additionally, in RNA-seq analysis, we identified common regulation ceRNA network including 3 DElncRNA (URS0000B28F2F, LOC100911498, URS0000B2E4CC), 5 DEmiRNA (novel-rno-miR-297-3p, rno-miR-425-3p, rno-miR-323-5p, rno-miR-3556a, rno-miR-181d-5p) and 109 DEmRNA between photoreceptor degenerations and control groups, suggesting the common ceRNA regulation network may facilitate the understanding of the mechanisms and benefit for the therapy in photoreceptor degeneration diseases. Several identified lncRNAs play crucial roles in neuron function and maintenance in neurodegenerative diseases, such as Tug1, participating in normal photoreceptor development and contributing to rod differentiation and survival (Carrella et al., 2020), and Meg3, regulating the process of light-induced retinal damage (Zhu et al., 2018). In our study, three lncRNAs (URS0000B28F2F, LOC100911498 and URS0000B2E4CC) we screened were significantly upregulated in photoreceptor degeneration models. The study reported that LOC100911498 is a novel regulator of neuropathic pain in rats by regulating the expression and function of the P2 × 4R (Tang et al., 2021). However, in ophthalmic diseases, these three lncRNAs have not been reported yet and the mechanism for regulating downstream miRNAs and influencing mRNA expression is still unclear. MiRNAs are a class of non-coding RNAs that regulate gene expression at the post-transcriptional level either by inhibiting mRNA translation or by promoting mRNA degradation (Correia de Sousa et al., 2019). Additionally, several key miRNAs have been identified to regulate retinal development in degenerative diseases, such as miR-182, miR-181, and miR-96 (Carrella et al., 2015; Fan et al., 2017). Among the five miRNAs we screened, miR-425-3p and miR-181d-5p stand out due to their established associations with neurologically related disorders, offering intriguing insights into their potential roles in the context of photoreceptor degeneration and retinal biology as a whole. In a notable study by Dakterzada et al. (2020), miR-425-3p was found to exhibit elevated expression levels in Alzheimer’s disease, a condition characterized by progressive cognitive decline and neurodegeneration. Remarkably, the researchers proposed miR-425-3p as a stable plasma endogenous control, suggesting its potential utility in disease monitoring and diagnostic applications. This finding underscores the multifaceted nature of miRNAs, which can serve not only as biomarkers but also as active players in the complex regulatory networks governing neuronal health and function. Equally compelling is the work of Shen et al. (2017), who shed light on the therapeutic potential of miR-181d-5p in the realm of neuroregeneration. Their study demonstrated that miR-181d-5p plays a pivotal role in promoting neurite outgrowth in PC12 cells through the activation of the PI3K/Akt signaling pathway. Furthermore, it was shown to alleviate spinal cord injury, emphasizing its significance in neural repair and recovery. This particular miRNA’s ability to stimulate neurite growth and enhance neuronal plasticity suggests a broader capacity to influence neuronal health, which could extend to the intricate processes involved in photoreceptor degeneration and retinal homeostasis. While their roles in neurologically related disorders have been well-documented, their potential relevance in the context of photoreceptor degeneration remains a promising avenue for further investigation. Understanding how miR-425-3p and miR-181d-5p function in the retina, particularly in the regulation of gene expression and cellular processes, may yield novel therapeutic strategies and a deeper appreciation of the complexities of retinal biology.

The GO terms of the co-DEmRNAs in the ceRNA network were enriched predominantly to “extracellular matrix organization” which modulates immune function (Boyd and Thomas, 2017) and facilitate nerve regeneration (Wang H. et al., 2022). In KEGG pathway analysis, the top enriched pathway was: “Complement and coagulation cascades.” Previous studies showed that “Complement and coagulation cascades” could be involved in the pathogenesis of EYS gene-deficient associated retinitis pigmentosa (RP) (Rai et al., 2022) and Age-related macular degeneration (Santos et al., 2023), which were in accordance with our results. These results of function enrichment analysis indicated that the immune system might play a prominent role in both primary and secondary photoreceptor degeneration. To further explore the potential interrelationship of co-DEGs from ceRNA network in both photoreceptor degeneration, PPI networks were constructed and the top 8 hub genes were selected. These hub genes were up-regulated and enriched in the immune system process. Herein, immune cell infiltration analysis was performed on hub genes. Previous studies (Murakami et al., 2012; Newton and Megaw, 2020) demonstrated that CD26 positive-T lymphocytes were elevated in the peripheral blood in most RP patients, especially in autosomal dominant RP. Williams et al. (1992) obtained a similar result: dual epitope increases for CD4+, CD26+ (activated helper), and CD8+, CD26+ (activated cytotoxic) cells in 10 laser immunostimulated RP patients. Additionally, the activated microglia and macrophage cell elevated before or during the early stages of photoreceptor degeneration in post-mortem samples from RP patients and several animal models of RP, such as in rd1 and rd10 mice (Zeng et al., 2005). It is worth noting that activated microglia play an important role in the neurodegeneration process by initiating the inflammatory response and facilitating cell apoptosis (Rathnasamy et al., 2019). Oka et al. (2008) have demonstrated that microglia cells become activated in response to the altered physiology of mutation-bearing photoreceptors. This activation prompts the production of proinflammatory cytokines and chemokines, including TNF-α and IL-1β, both of which are widely implicated in retinal degenerative diseases. Furthermore, the oxidative activation of microglial can initiate a detrimental cycle of non-resolved neuroinflammation and degeneration in RP, highlighting the pivotal role of microglia as a primary target of oxidative stress in RP (Conti et al., 2020). The above results revealed that the activated T lymphocytes and microglia cells increased in RP patients, which is in line with our immune analysis results, indicating that these two types of immune cells may be involved in the progression of photoreceptor degeneration. Other studies report CD8 T cells decreased, but CD4 T cells unchanged or decreased in peripheral blood in RP patients (Hendricks and Fishman, 1985; Newsome et al., 1988). The different results suggested that a range of immune system dynamics among patients of different mutated genes, various induced drugs and diverse stages of retinal degeneration would be expected.

CeRNAs can control gene expression by regulating post-transcription levels by competing for shared miRNAs. We can obtain more details about the impact of hub gene expression on two photoreceptor degenerations by studying the ceRNA network. These hub genes of this ceRNA network are related to various immune activities, including regulating M2 macrophage function or T/B cell late differentiation. For example, Cyth4, a member of the ARF-GEF family, can regulate protein sorting, membrane trafficking and various immunomodulatory activities (Sztul et al., 2019). Guan et al. (2023) presented that Cyth4 activated M2 macrophages’ protumor function which promoted tumor growth and metastasis by secreting protumor cytokines in glioma. Similarly, Fn1 expression has been closely linked to M2 macrophages which affects the immunological microenvironment and leads to the progression of gastric cancers (Wang H. et al., 2022). Besides, Huang S. et al. (2022) presented that LINC02381 might change the tumor immune microenvironment, exacerbating breast cancer through the miR-1271-5p/FN1 axis. IKZF1 is a key transcription factor in regulating leukocyte differentiation and its variants cause abnormal T/B cell late differentiation (Hoshino et al., 2022). The study reported that the miR-128 expression was significantly lower in patients with Ikzf1 deletion than those without Ikzf1 deletion in B-cell precursor acute lymphoblastic leukemia (Krzanowski et al., 2017). Importantly, these hub genes not only participate in immune regulation but also play important roles in neurodegenerative diseases through the ceRNA network regulation (Javed et al., 2023; Yi et al., 2023). Qin et al. (2023) revealed that miR-6734-3p and its target mRNA Cyth4 expression were correlated with patients’ age in Alzheimer’s disease. Xie et al. (2020) presented that miR-183 regulated lipopolysaccharide-induced oxidative stress in hippocampal neurons by targeting Fn1 expression to minimize oxidative damage, suggesting miR-183/Fn1 axis was important in oxidative stress-associated neurodegenerative diseases. Additionally, the other hub genes, C1qb, C1qa, Wdfy4, Card11, and Csf2rb are associated with the immune process through ceRNA network regulation, but no studies reported them in photoreceptor degeneration, indicating these hub genes might be potential and novel therapeutic targets (Li Y. et al., 2021; Zhu et al., 2022). Additionally, we found 7 immunotherapy drug-targeting hub genes. Most of them are anticancer drugs with a high activity against hematological malignancies including lymphoma and leukemia. Besides, several studies revealed that SARGRAMOSTIM, LENALIDOMIDE, METHOTREXATE, CYTARABINE and IMATINIB have crucial roles in the treatment of neurodegenerative disorders, such as Parkinson’s disease and Alzheimer’s disease (Mareček and Rudá-Kučerová, 2018; Kumar et al., 2019; Decourt et al., 2020; Olson et al., 2023). Potter et al. (2021) found that SARGRAMOSTIM therapy was safe and well-tolerated and provided memory-enhancing benefits to patients with mild-to-moderate Alzheimer’s disease. Additionally, IMATINIB has the potential to ameliorate the death of dopaminergic neurons in Parkinson’s disease by inhibiting the p38-MAPK/p53/α-synuclein signaling pathway (Alves Da Costa et al., 2002). While these drugs have yet to be explored in the context of photoreceptor degeneration, the promising findings in neurodegenerative disorders hint at a potential avenue for future research and therapeutic interventions in retinal diseases.

Finally, we combined hub genes and related miRNAs and lncRNAs to reconstruct the ceRNA network and performed RT-qPCR validation for these genes. Based on previous studies, the effect of most ceRNA networks was identified in ophthalmic physiopathological processes, suggesting our result might help understand the mechanism of primary and secondary photoreceptor degenerations (Chen et al., 2021; Huang Y. et al., 2022; Wang N. et al., 2022). However, there were several limitations in this study. The RNA-seq data were obtained from small samples, which may cause selected bias. CeRNA network regulations are needed for further verification by immunoprecipitation assays and dual-luciferase reporter assays. Furthermore, additional *in vitro* and *in vivo* experiments are required to identify the function of hub genes and further investigate the potential mechanisms underlying photoreceptor degeneration.

## Conclusion

We constructed a shared key ceRNA network with 2 lncRNAs, 2 miRNAs and 8 mRNAs in primary and secondary photoreceptor degeneration and revealed that both photoreceptor degenerations were related to immune infiltration. CeRNA-related-hub genes were identified as vital in immune regulation in various diseases. These findings provide some key clues for further understanding underlying mechanisms and exploring treatment targets in the future. We hope our results provide a new strategy to alleviate photoreceptor degeneration.

## Data availability statement

The datasets presented in this study can be found in online repositories. The names of the repository/repositories and accession number(s) can be found in this article/Supplementary material.

## Ethics statement

The animal studies were approved by the Animal Ethical Committee of Jinan University (Guangdong, China). The studies were conducted in accordance with the local legislation and institutional requirements. Written informed consent was obtained from the owners for the participation of their animals in this study.

## Author contributions

JL: Conceptualization, Data curation, Formal analysis, Investigation, Methodology, Project administration, Resources, Software, Supervision, Validation, Writing – original draft, Writing – review & editing. DF: Data curation, Formal analysis, Investigation, Methodology, Project administration, Resources, Software, Validation, Visualization, Writing – original draft, Writing – review & editing. FY: Conceptualization, Formal analysis, Methodology, Project administration, Software, Supervision, Writing – review & editing. LC: Conceptualization, Formal analysis, Investigation, Methodology, Project administration, Software, Writing – review & editing. ZZ: Conceptualization: Formal analysis, Resources, Supervision, Writing – review & editing. XT: Data curation, Formal analysis, Resources, Validation, Writing – review & editing. LF: Formal analysis, Investigation, Software, Supervision, Writing – review & editing. YZ: Data curation, Project administration, Resources, Visualization, Writing – review & editing. TX: Data curation, Investigation, Project administration, Software, Supervision, Writing – review & editing. PW: Investigation, Methodology, Resources, Validation, Writing – review & editing. PL: Conceptualization, Data curation, Validation, Writing – review & editing. HZ: Formal analysis, Supervision, Validation, Writing – review & editing. SZ: Conceptualization, Data curation, Formal analysis, Funding acquisition, Methodology, Project administration, Resources, Software, Validation, Visualization, Writing – original draft, Writing – review & editing.

## Footnotes

1
https://biosys.bgi.com

2
https://www.bioinformatics.com.cn

3
https://www.dgidb.org

4
https://international.biocloud.net/zh/software/tools/heatmap
