# Supplementary material for: Analysis of shared ceRNA networks and related-hub genes in rats with primary and secondary photoreceptor degeneration
Source: Front Neurosci. 2023 Sep 21;17:1259622. doi: 10.3389/fnins.2023.1259622 (PMC10552924; doi:10.3389/fnins.2023.1259622)
Supplement: Supplementary file 1 [file Data_Sheet_1.ZIP › Original data/The illustration of making figures.docx]

Dear Editors,

We performed RNA sequencing to analyze shared differentially expressed lncRNAs, miRNAs, and mRNAs in the MNU and RCS groups compared to the RDY group. RNA sequencing was performed using the DNBSEQ platform (BGI) (Huada Gene Technologies Ltd., Guangdong, China). The data were analyzed on the Dr. Tom II network platform of the BGI (https://biosys.bgi.com/). The raw data of RNA-seq has been submitted on NCBI (PRJNA989559). Besides, we have provided each figure's original data and manipulations. We have relied mainly on the Dr. Tom II network platform and several publicly available databases to analyze and make the figures, and we have described the specific methods below to make sure it repeatable.

In **Figure 1**, we provide the data（Table 1）for each graph and the statistical analysis results { SPSS 22.0 (IBM, Armonk, NY, USA)}. We also provide original microscopy scans and OCT images of the retina in RDY, MNU and RCS groups (four per group). We use CaseViewer software (https://www.3dhistech.com/solutions/caseviewer/) to analyze microscopy scans of HE-stained retinal tissues. We measured the thickness of the ONL at 800, 1600, 2400, 3200, and 4000μm away from the optic never head（Figure 1）, and the ONL cell numbers of the retina from the optic never head to about 2000μm away from the optic never head（Figure 2）.

In **Figure 2**, we analyzed the differentially expressed lncRNA, miRNA, and mRNA in the MNU group and the RCS group compared to the RDY group using the Dr. Tom II network platform of the BGI (https://biosys.bgi.com/). The volcano plots and Venn diagrams for each group's three types of differentially expressed RNAs were drawn. (setting the absolute value of lg FC to be larger than 1.5 for lncRNA and miRNA, and the absolute value of lg FC to be greater than 2 for mRNA, all Q values to be less than 0.05) (Table 1). Results 1-3 show the co-differentially expressed lncRNA, miRNA, and mRNA data in the two photoreceptor degeneration groups obtained from Venn diagrams.

In **Figure 3**, we utilize the target prediction function of the Dr. Tom II network platform (https://biosys.bgi.com/), which is also based on three databases (RNAhybrid/ MiRanda/ Targetscan) to achieve target prediction. Firstly, we performed miRNA target prediction by shared 2 up-regulated and 35 down-regulated LncRNAs (Table 1), and the system predicted 226 down-regulated miRNAs and 543 up-regulated miRNAs based on the reverse expression relationship between the LncRNAs and miRNAs (Table 2). Intersections were taken with shared expressed miRNAs in two photoreceptor degenerations (Table 3) to obtain a 4 lncRNA - 6 miRNA network. Finally, excluding the lncRNAs localized in the nucleus, a 3 lncRNA - 5 miRNA network was obtained. (Table 4) Similarly, 5 miRNAs were targeted to predict mRNAs (Table 5), and the obtained mRNAs were intersected with shared expressed mRNAs in two photoreceptor degenerations (Table 6) to obtain 109 mRNAs (Table 7). Finally, the co-regulated ceRNA network was obtained, which included 3 lncRNAs, 5 miRNAs and 109 mRNAs (Table 8).

In **Figure 4**, we use Cytoscape software (version 3.9.1) to visualize the previously obtained ceRNA network. We input the Table 1 and 2 in Cytoscape to get Figure 4A. Input the expression amount of each sample of the three groups (Table 3), and then we can get the expression heatmap (https://international.biocloud.net/zh/software/tools/heatmap)

In **Figure 5**, we analyzed the 109 shared DEmRNAs obtained above by using the Go enrichment function of the Dr. Tom II network platform (https://biosys.bgi.com/). We obtained the GO-CFP bar chart and 3 bubble charts with the top 20 significant biological processes (GO-P), cellular components (GO-C), and molecular functions (GO-F), respectively.

In **Figure 6**, we performed string mapping of 109 shared DEmRNAs. Firstly, we input 109 genes into Metascape for KEGG enrichment analysis to obtain 11 terms (Table 1), which were collated into Table 2. Table 2 is available on the bioinformatics Analytics platform (https://www.bioinformatics.com.cn/plot_basic_GOplot_).chord_plot_085) to get the KEGG chord plot.

In **Figure 7**, we used the STRING online database to analyze interactions of 109 proteins (Table 1), which was visualized by Cytoscape software (version 3.9.1). Next, we used the small plug-in MCODE in Cytoscape to identify the hub genes (Table 2). According to the node degree method, 14 hub genes were ranked by cytoHubba, and the top 8 hub genes (C1qbC1qb, C1qa, Cyth4, Wdfy4, Fn1, Ikzf1, Card11, Csf2rb) were selected for further analysis (Table 3).

In **Figure 8**, we performed enrichment analysis after inputting 8 hub gene names in the Metascape database and obtained the enrichment bar graph (Figure 1). Then we input Table 1 in Immquant software to analyze the quantities of the immune cell types of hub genes. We get the immune analysis result (Table 2), sorted into Table 3. Input Table 3 in the bioinformatics analysis platform ((https://www.bioinformatics.com.cn/plot_basic_circular_cluster_heatmap_plot_070) to draw the circular cluster heatmap. Then, we input Table 4 in the bioinformatics analysis platform (https://www.bioinformatics.com.cn/plot_basic_corrplot_corrlation_plot_082) to obtain the matrix plot of immune cell correlation coefficients for the MNU group and RCS group. Finally, we analyzed the relative quantities of 11 immune cells in the three groups, and the results are shown in Table 5 (SPSS 22.0 (IBM, Armonk, NY, USA)). Besides, we performed Spearman correlation analysis between the 8 hub genes and the quantities of 11 types of immune cells. The results are shown in Table 6 (SPSS 22.0 (IBM, Armonk, NY, USA)), and imported the data into the bioinformatics analysis platform (https://www.bioinformatics.com.cn/plot_basic_corrlation_dot_line_054) to obtain a lollipop plot of the correlation coefficients.

In **Figure 9**, we performed drug-gene interaction analysis for 8 hub genes. Firstly, we input the hub genes in the DGIDB database (https://www.dgidb.org) and got 14 drugs targeting the genes, of which 7 were immunotherapeutic drugs (https://www.dgidb.org/search_interactions). We imported the results (Table 2,3) into Cytoscape for visualization. Next, we reconstructed the CeRNA network containing 8 hub genes and imported the data (Table 4) into the bioinformatics analysis platform ( https://www.bioinformatics.com.cn/plot_basic_alluvial_plot_017) to get the alluvial plot. Finally, we performed RT-qPCR validation of all CeRNAs where hub genes are located and the results are shown in Table 5. The solubilization and amplification curves of each RNA are also shown in File 1.
