# Supplementary figures and images for: Analysis of shared ceRNA networks and related-hub genes in rats with primary and secondary photoreceptor degeneration
Source: Front Neurosci. 2023 Sep 21;17:1259622. doi: 10.3389/fnins.2023.1259622 (PMC10552924; doi:10.3389/fnins.2023.1259622)

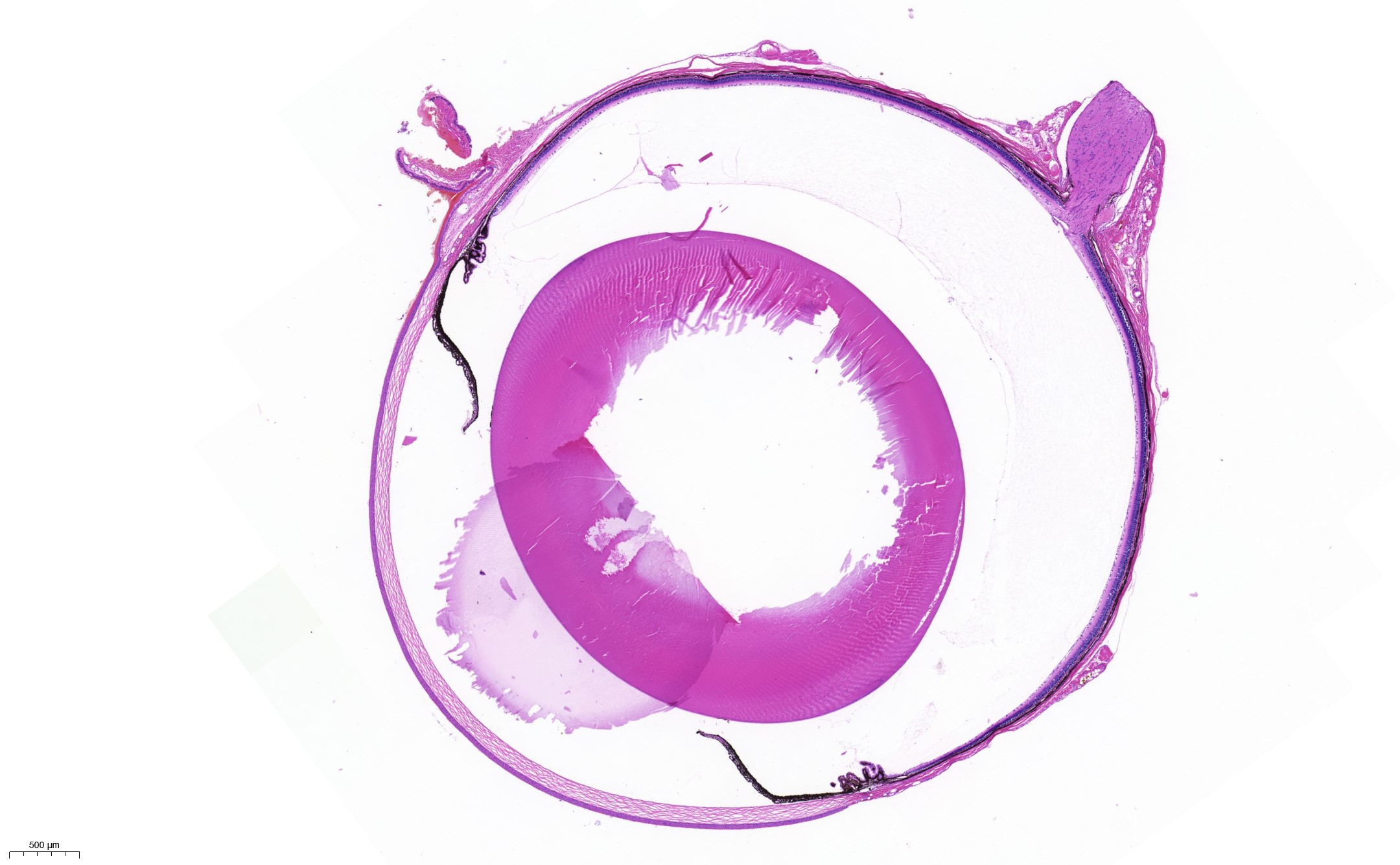

Supplement: Supplementary file 1 [file Data_Sheet_1.ZIP › Original data/Fig 1/HE-stained retina images/1.MNU/MNU-1 Day-1.jpg]

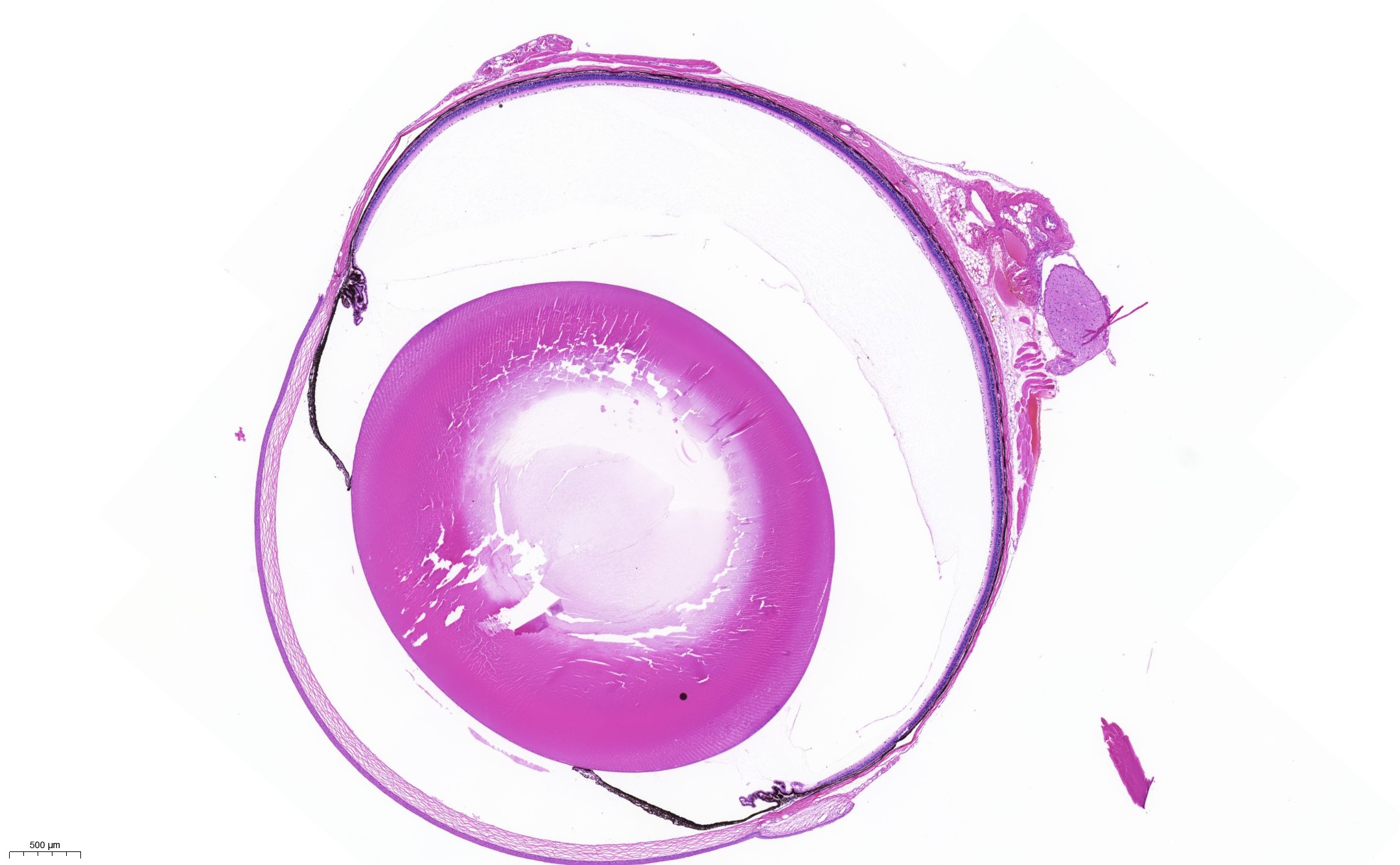

Supplement: Supplementary file 1 [file Data_Sheet_1.ZIP › Original data/Fig 1/HE-stained retina images/1.MNU/MNU-1 Day-2.jpg]

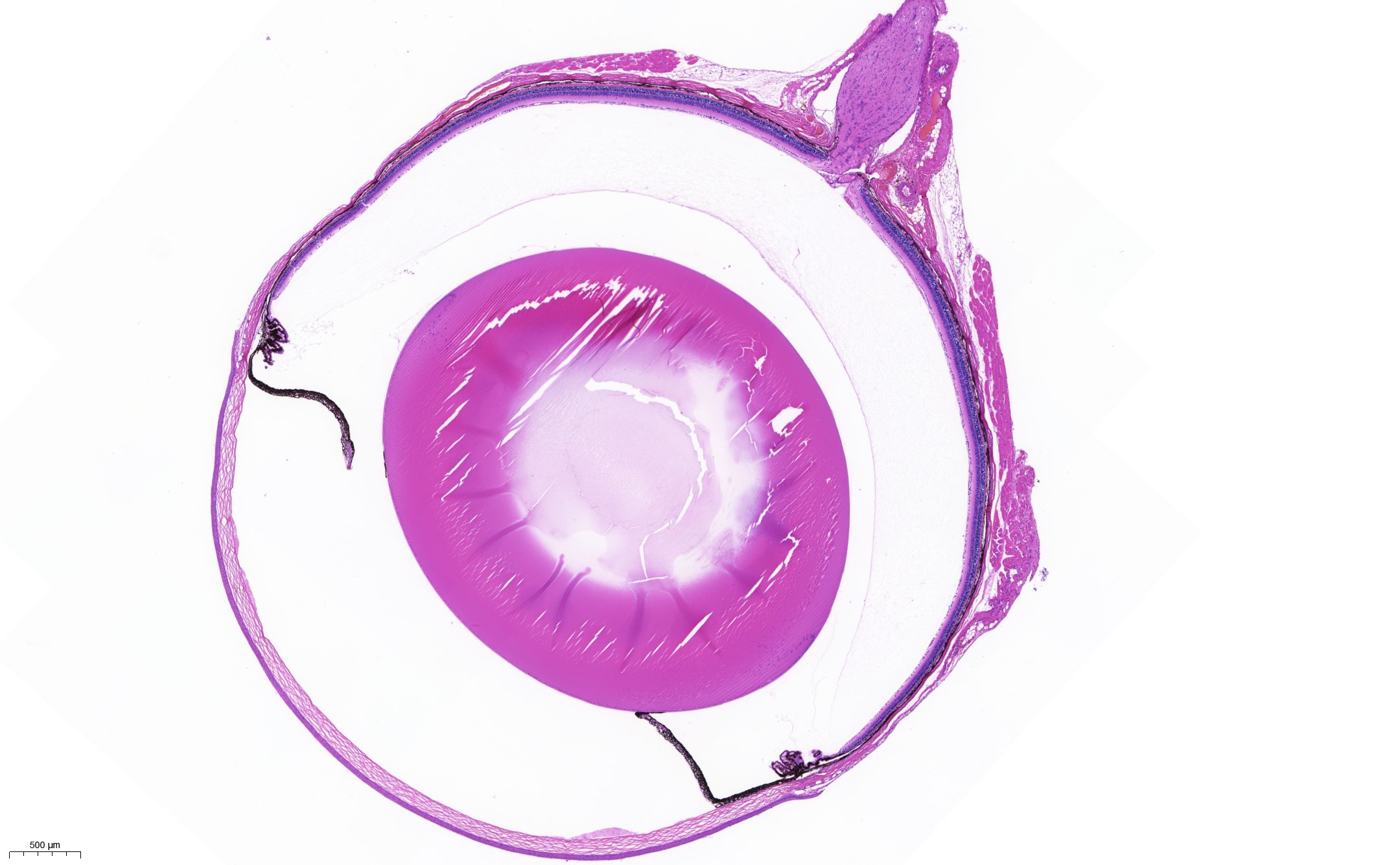

Supplement: Supplementary file 1 [file Data_Sheet_1.ZIP › Original data/Fig 1/HE-stained retina images/1.MNU/MNU-1 Day-3.jpg]

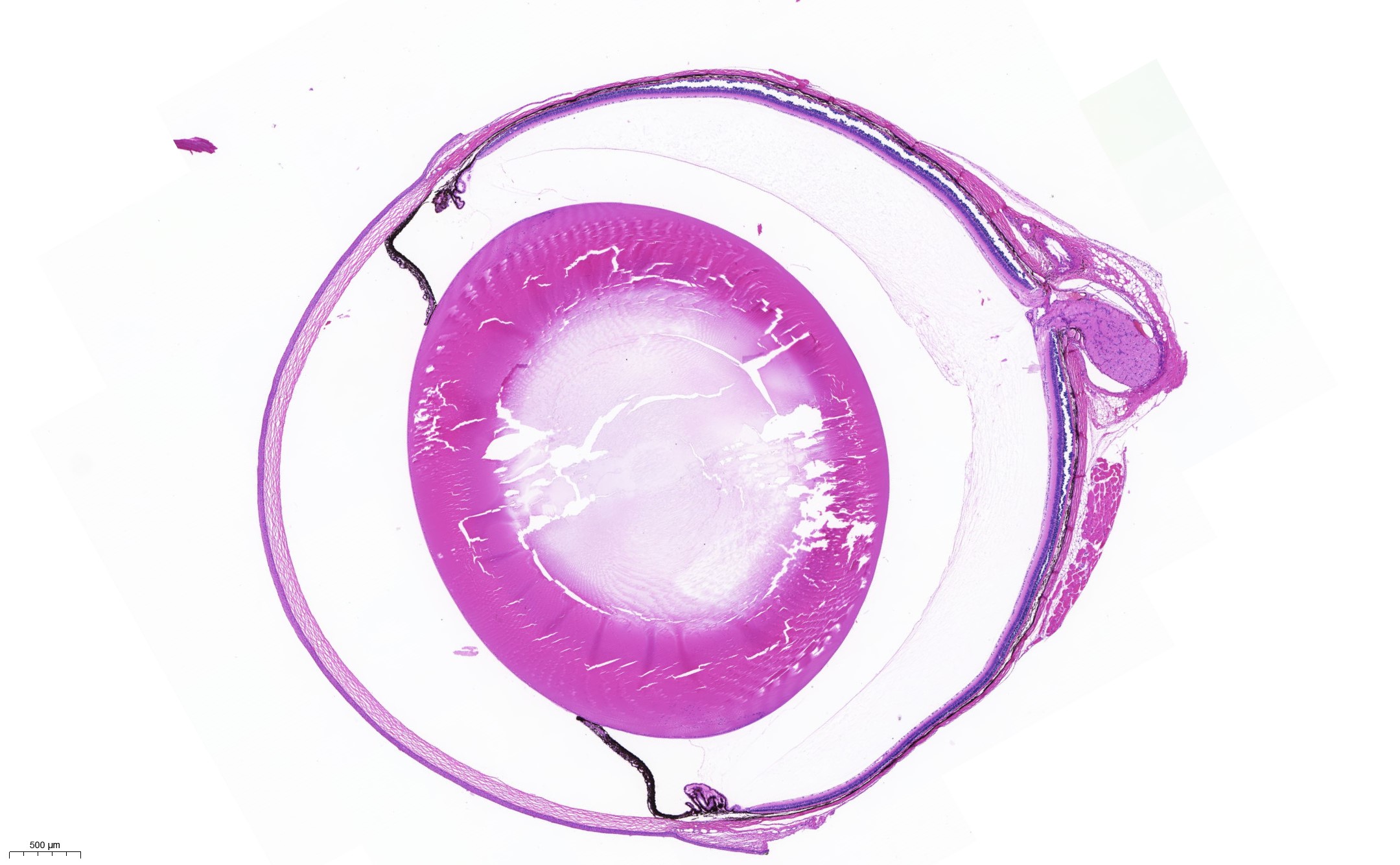

Supplement: Supplementary file 1 [file Data_Sheet_1.ZIP › Original data/Fig 1/HE-stained retina images/1.MNU/MNU-1 Day-4.jpg]

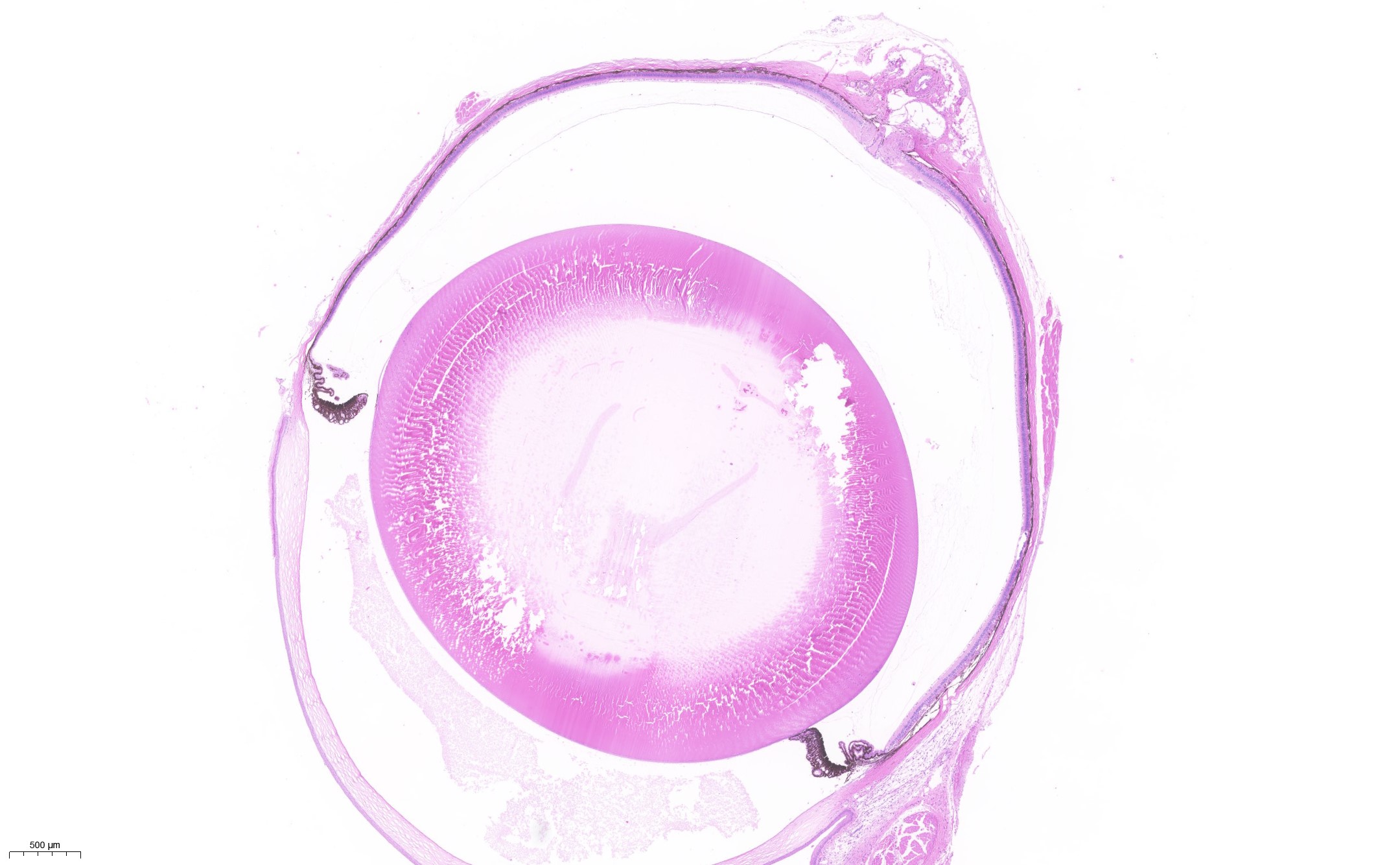

Supplement: Supplementary file 1 [file Data_Sheet_1.ZIP › Original data/Fig 1/HE-stained retina images/1.MNU/MNU-100 mg-1.jpg]

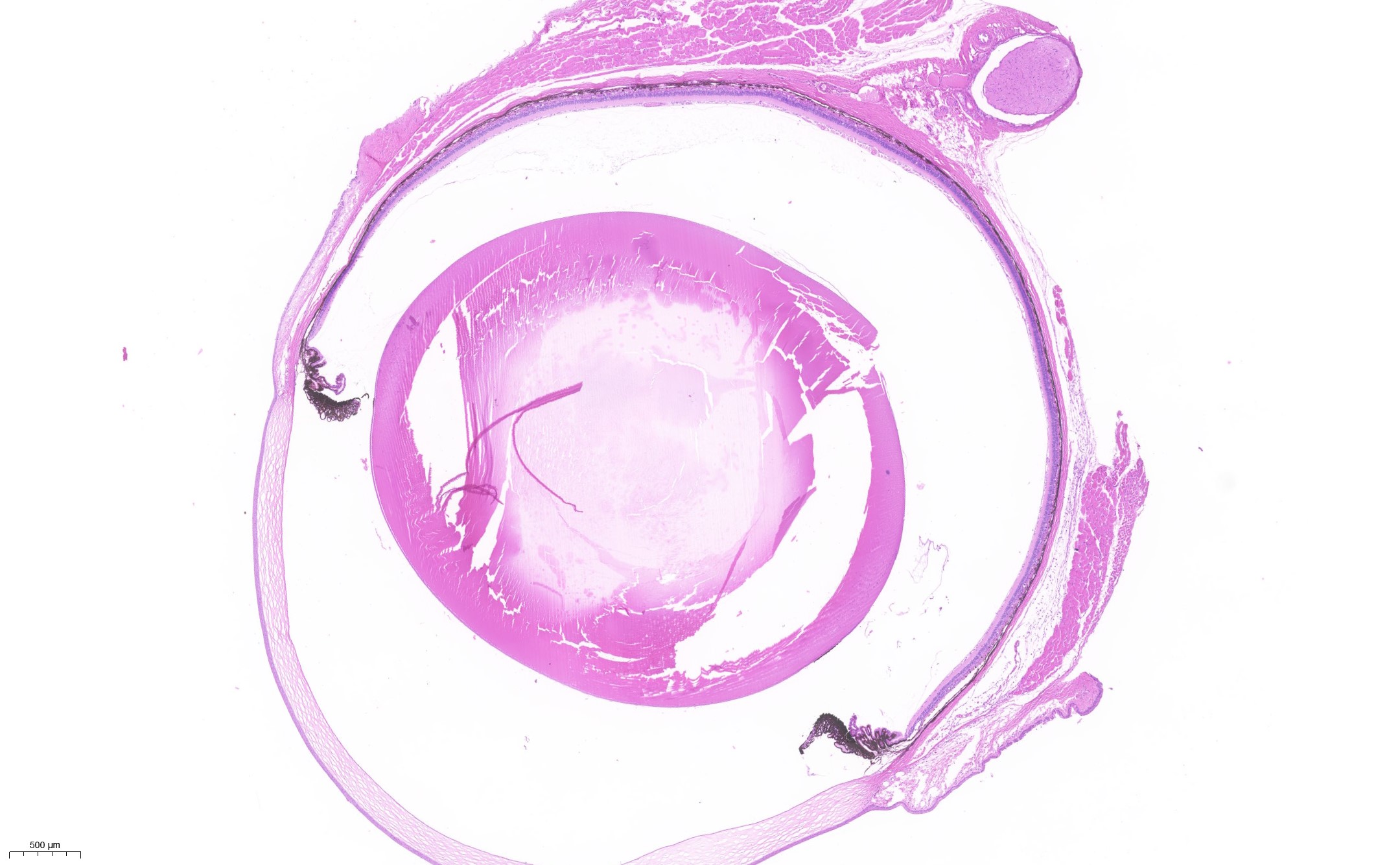

Supplement: Supplementary file 1 [file Data_Sheet_1.ZIP › Original data/Fig 1/HE-stained retina images/1.MNU/MNU-100 mg-2.jpg]

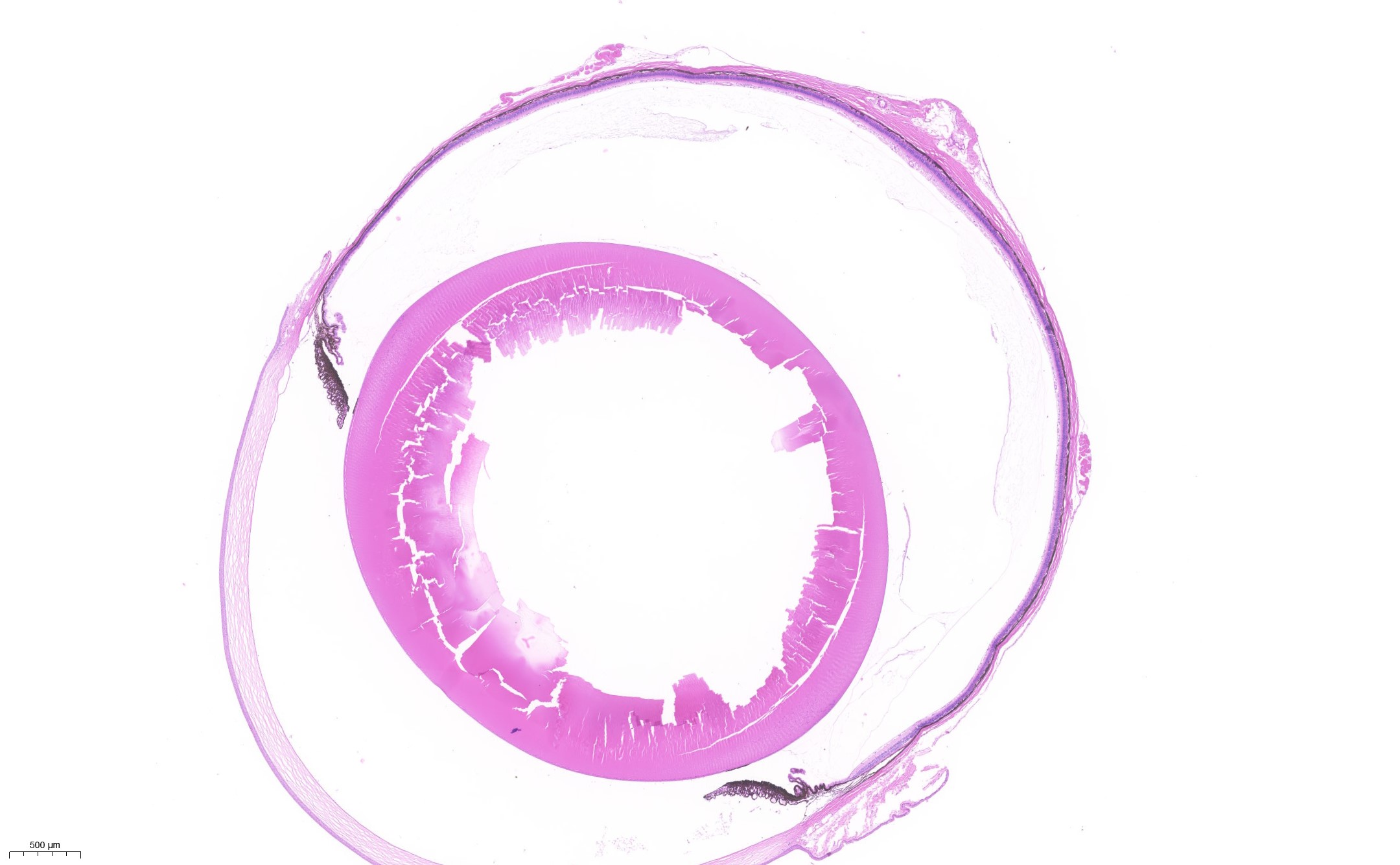

Supplement: Supplementary file 1 [file Data_Sheet_1.ZIP › Original data/Fig 1/HE-stained retina images/1.MNU/MNU-100 mg-3.jpg]

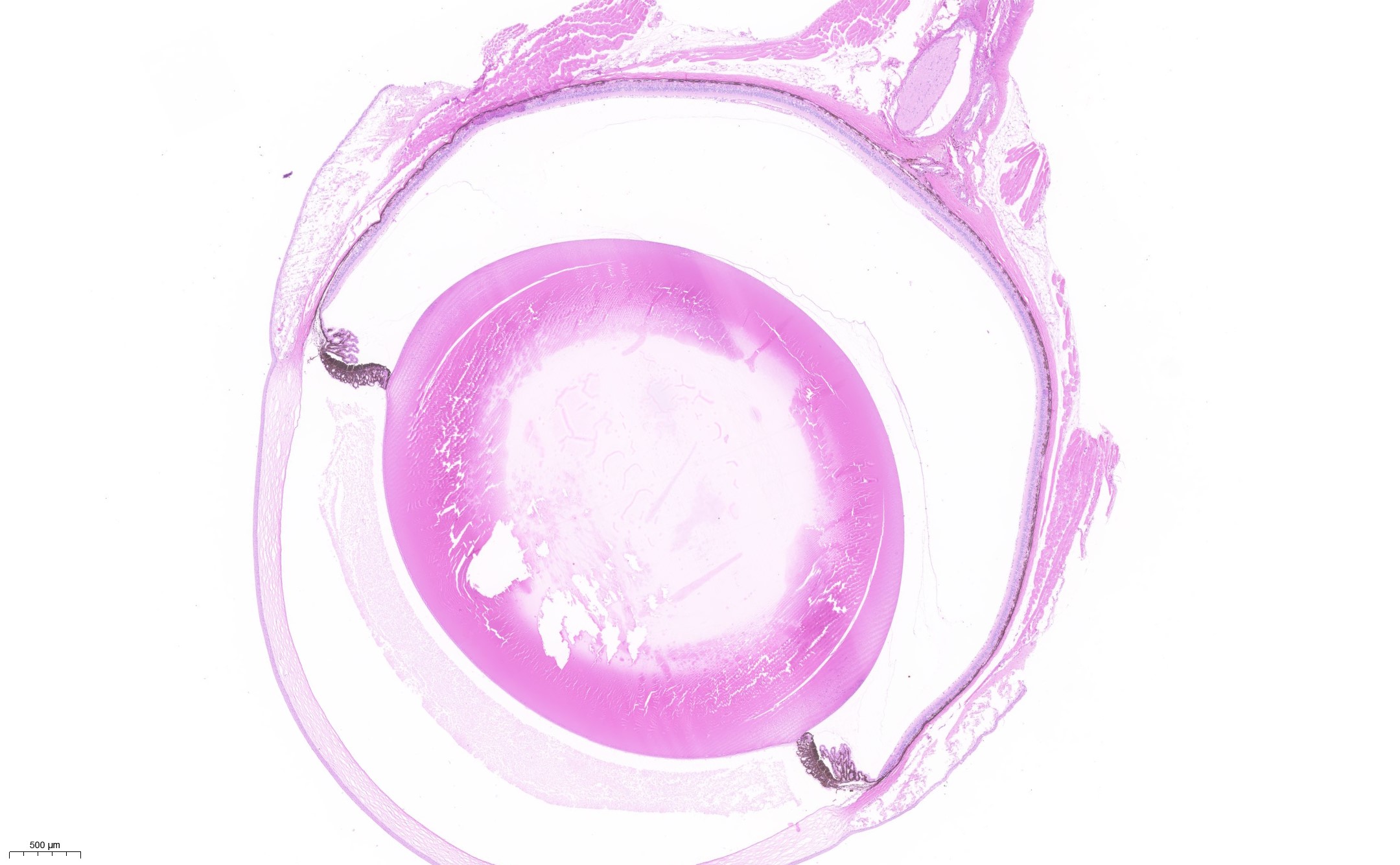

Supplement: Supplementary file 1 [file Data_Sheet_1.ZIP › Original data/Fig 1/HE-stained retina images/1.MNU/MNU-100 mg-4.jpg]

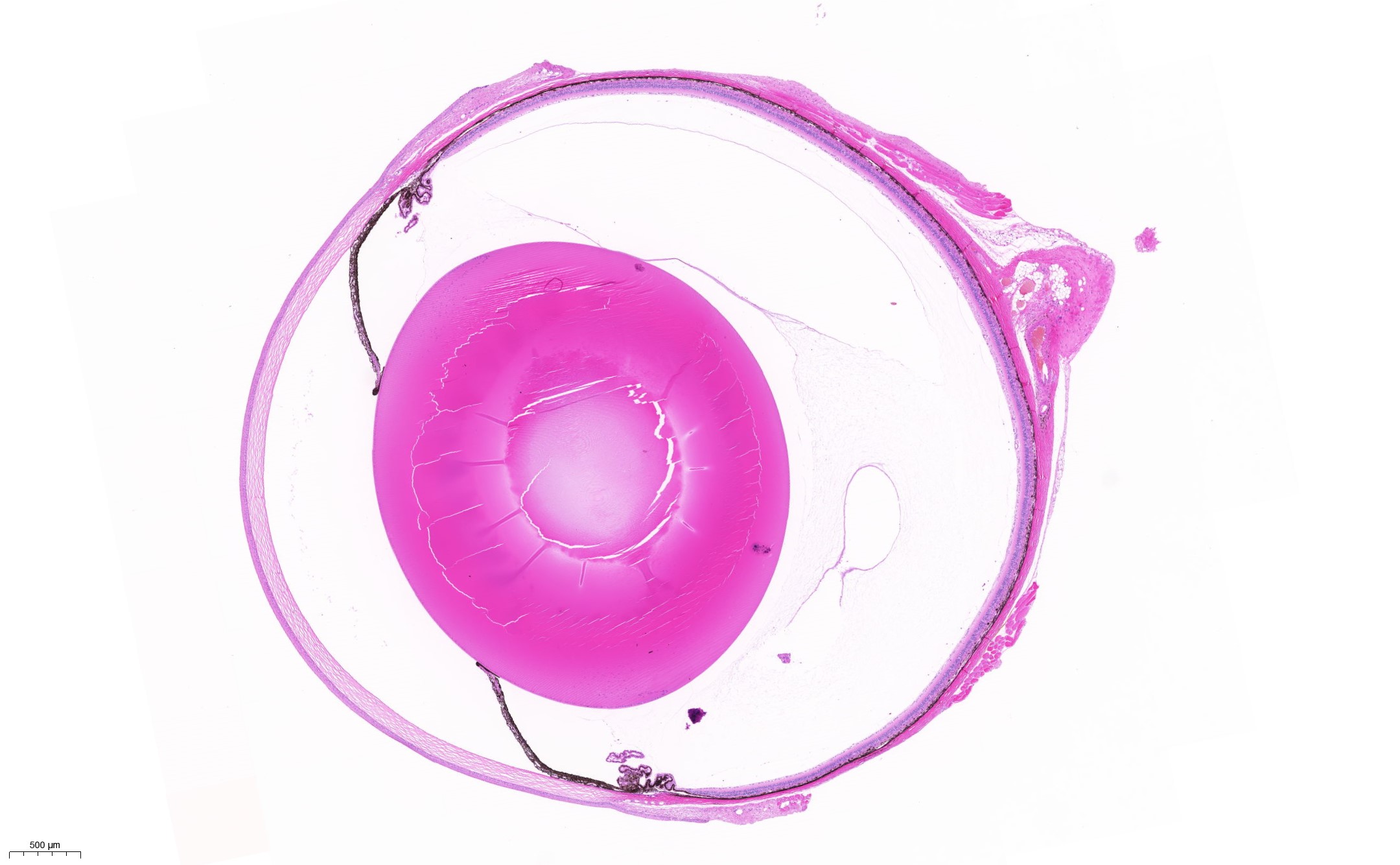

Supplement: Supplementary file 1 [file Data_Sheet_1.ZIP › Original data/Fig 1/HE-stained retina images/1.MNU/MNU-3 Day-1.jpg]

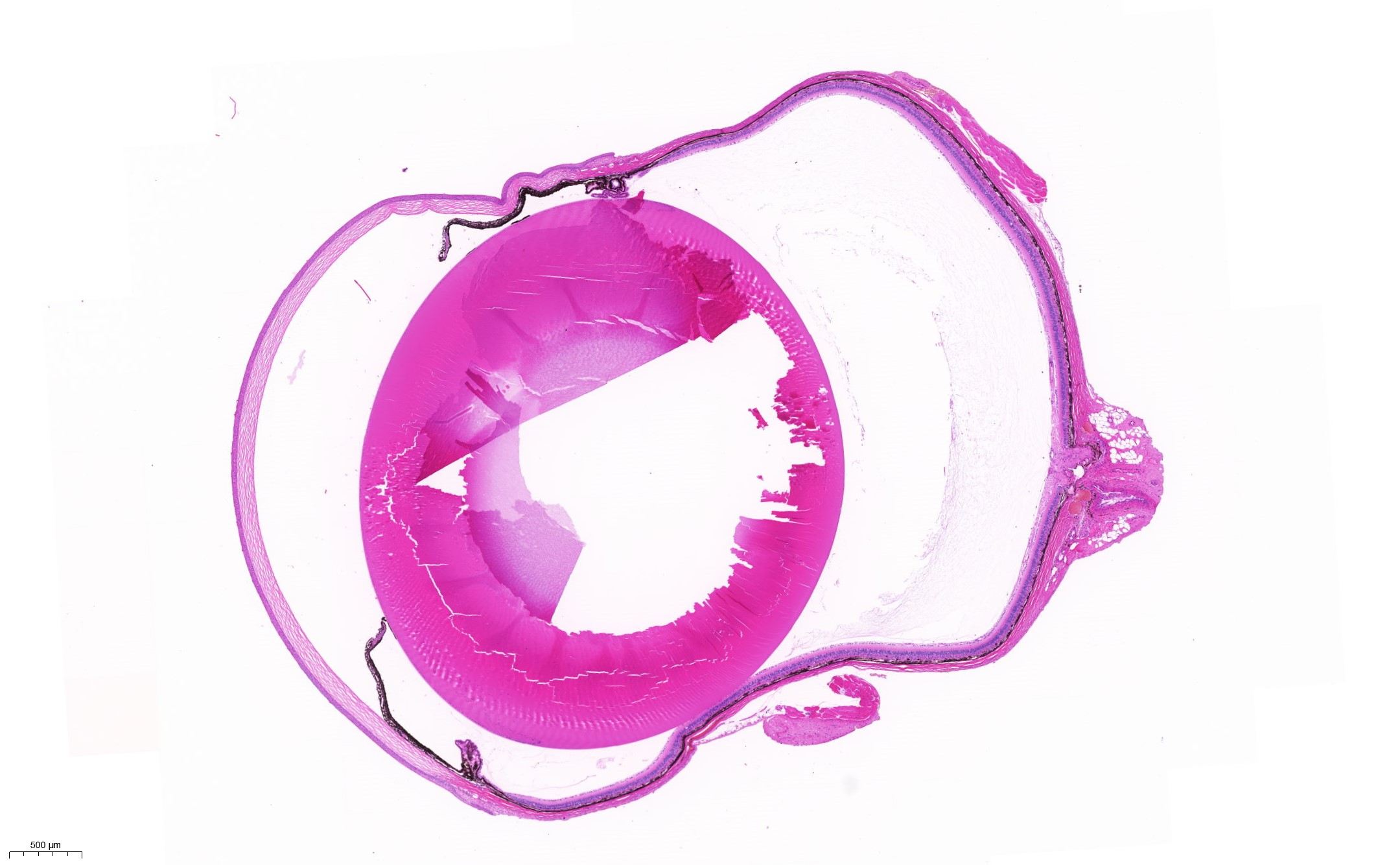

Supplement: Supplementary file 1 [file Data_Sheet_1.ZIP › Original data/Fig 1/HE-stained retina images/1.MNU/MNU-3 Day-2.jpg]

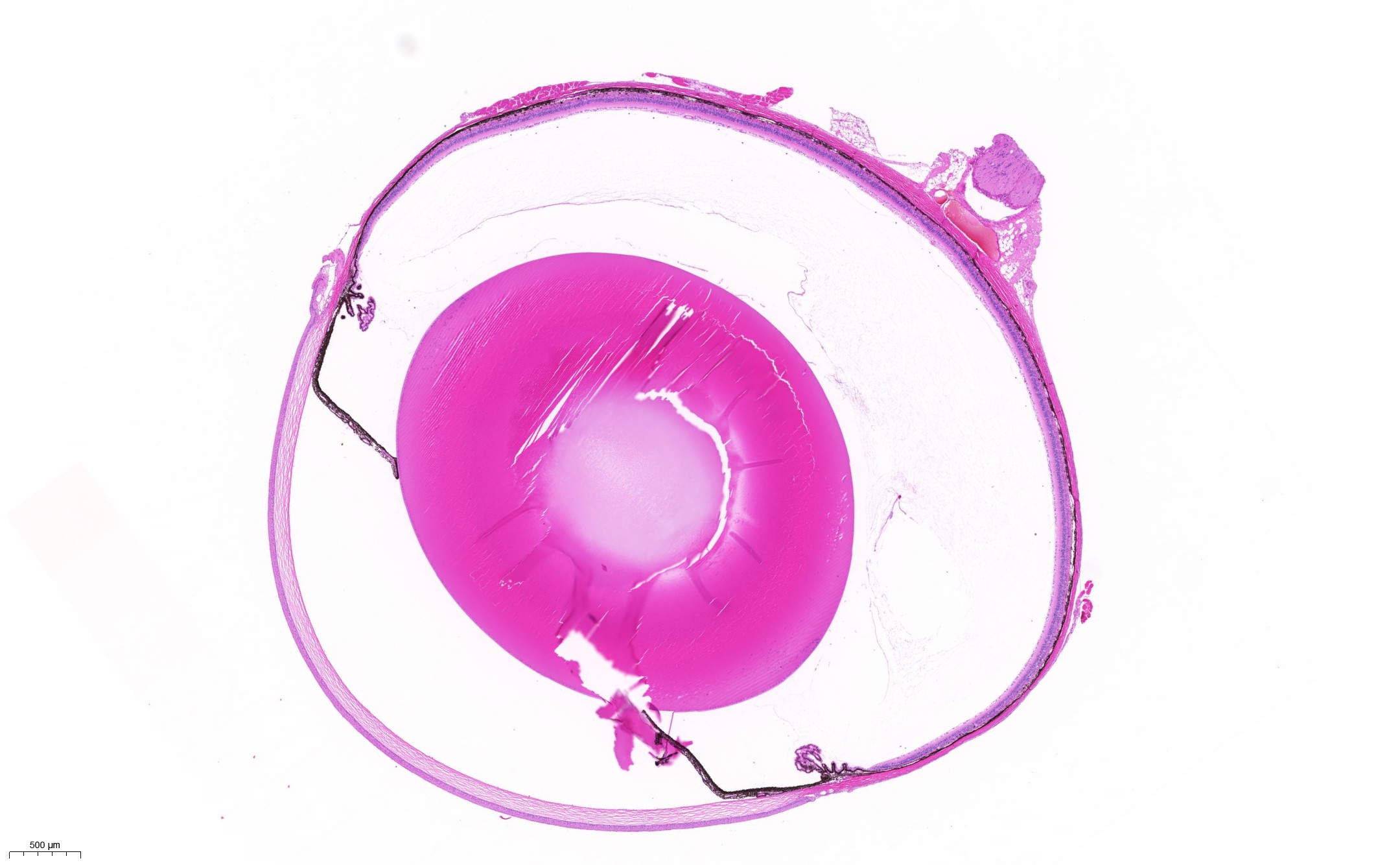

Supplement: Supplementary file 1 [file Data_Sheet_1.ZIP › Original data/Fig 1/HE-stained retina images/1.MNU/MNU-3 Day-3.jpg]

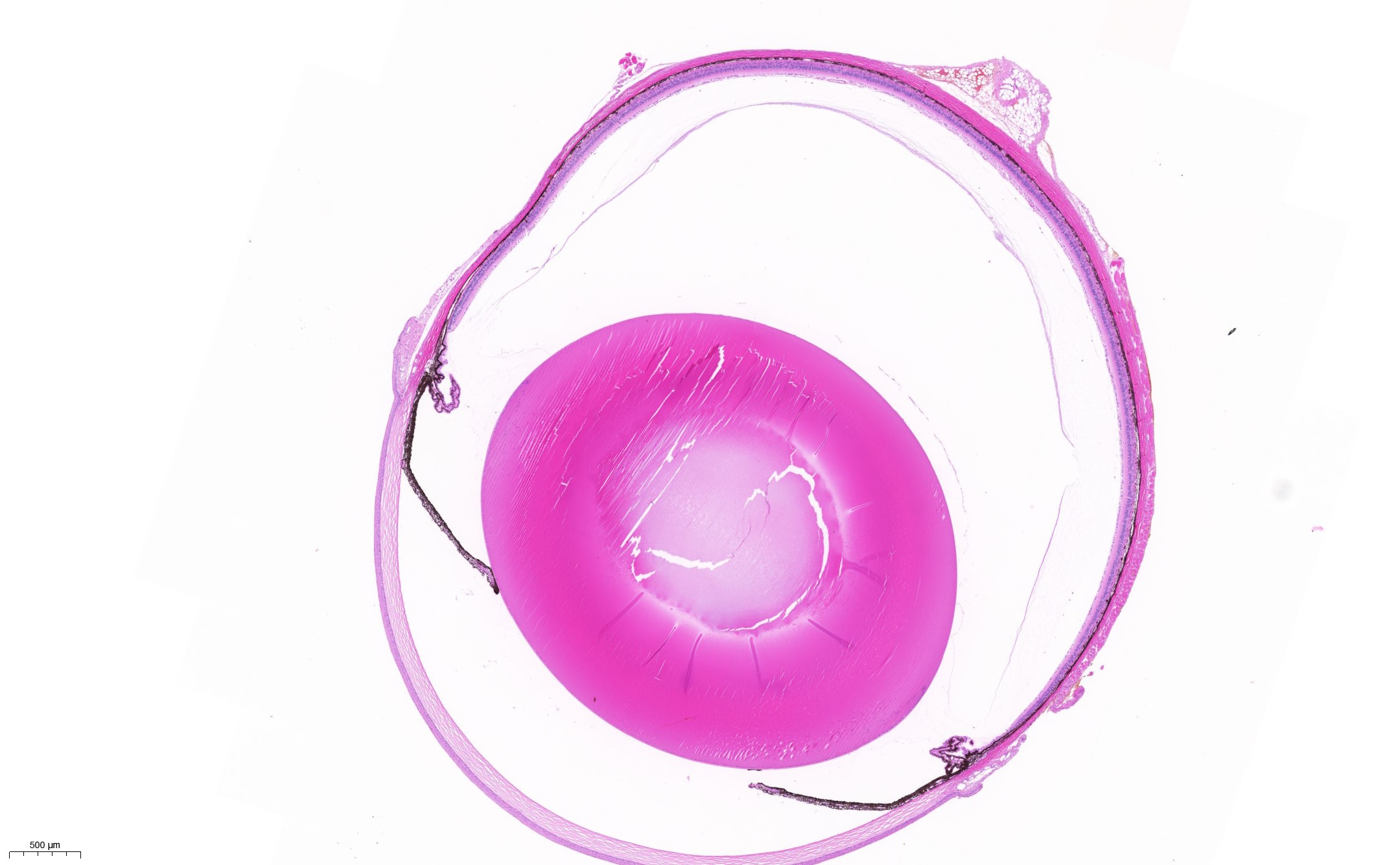

Supplement: Supplementary file 1 [file Data_Sheet_1.ZIP › Original data/Fig 1/HE-stained retina images/1.MNU/MNU-3 Day-4.jpg]

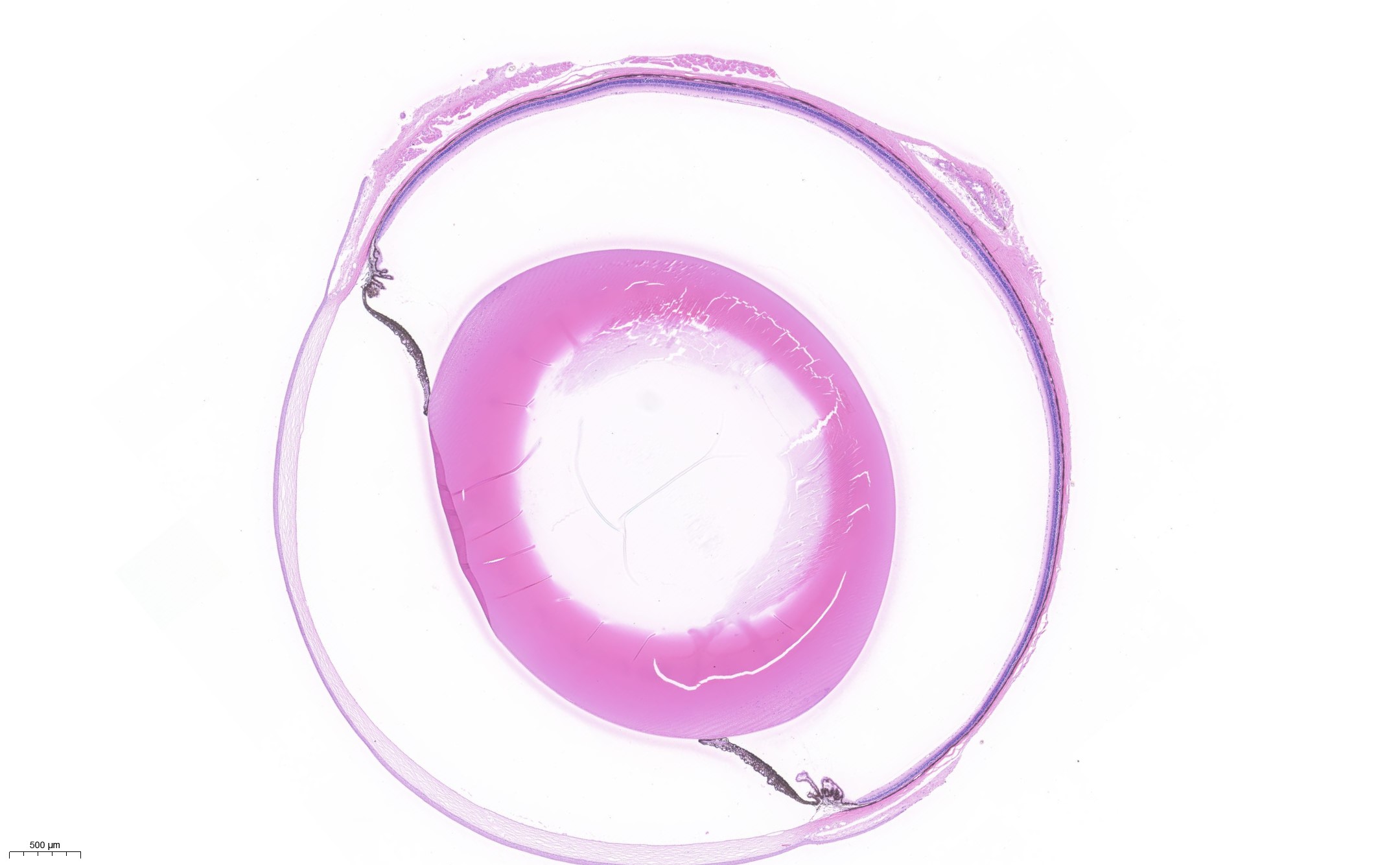

Supplement: Supplementary file 1 [file Data_Sheet_1.ZIP › Original data/Fig 1/HE-stained retina images/1.MNU/MNU-40 mg-1.jpg]

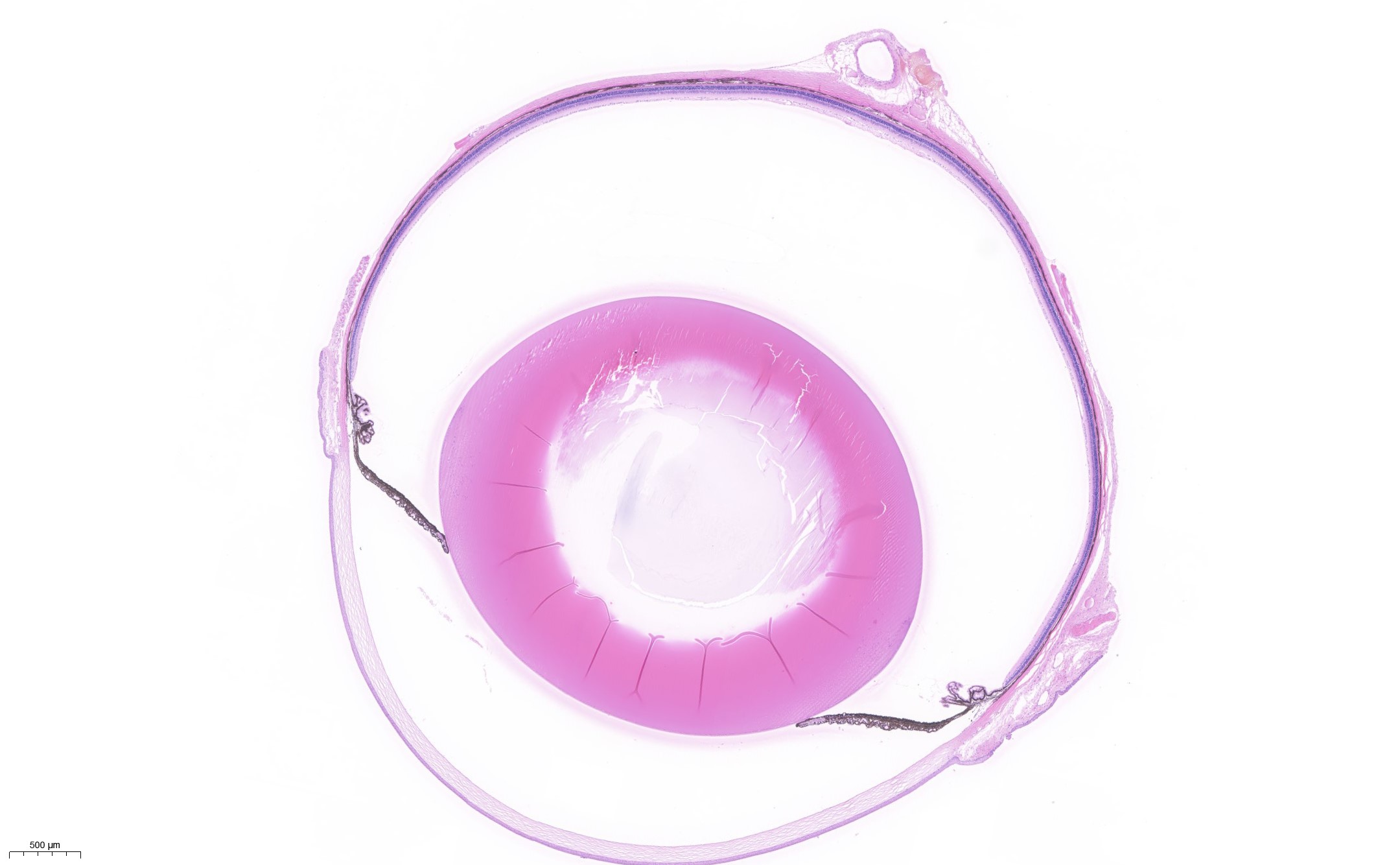

Supplement: Supplementary file 1 [file Data_Sheet_1.ZIP › Original data/Fig 1/HE-stained retina images/1.MNU/MNU-40 mg-2.jpg]

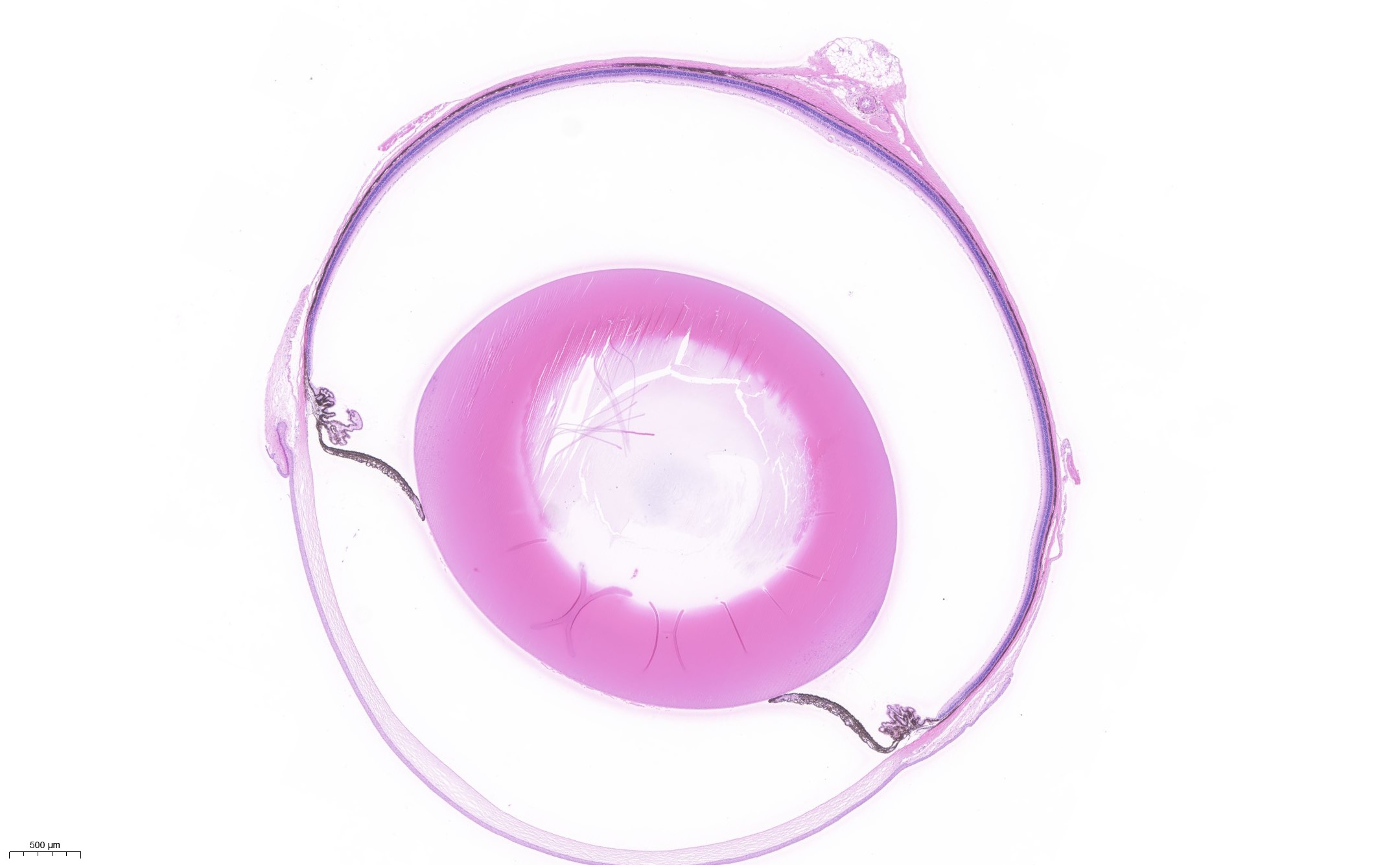

Supplement: Supplementary file 1 [file Data_Sheet_1.ZIP › Original data/Fig 1/HE-stained retina images/1.MNU/MNU-40 mg-3.jpg]

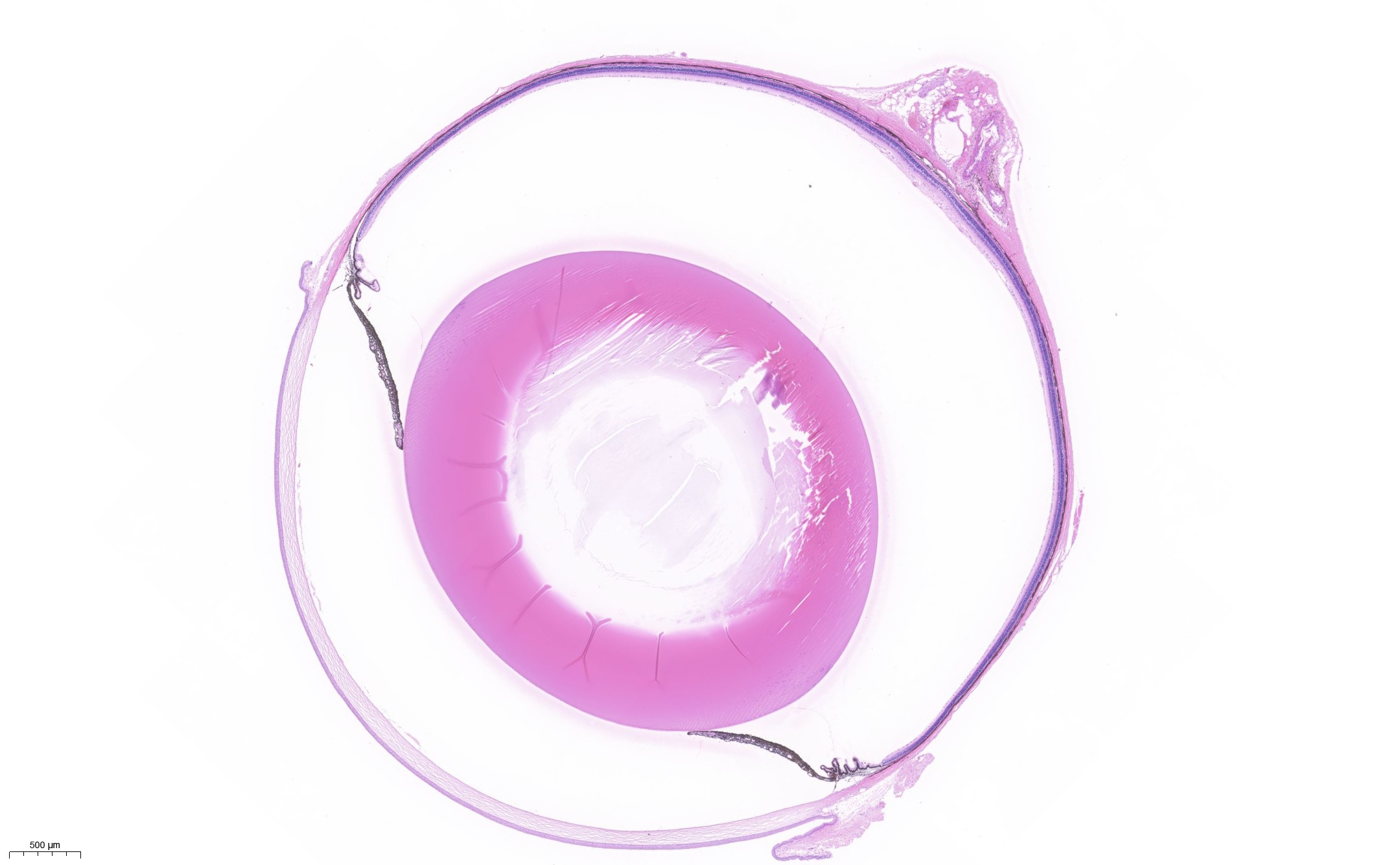

Supplement: Supplementary file 1 [file Data_Sheet_1.ZIP › Original data/Fig 1/HE-stained retina images/1.MNU/MNU-40 mg-4.jpg]

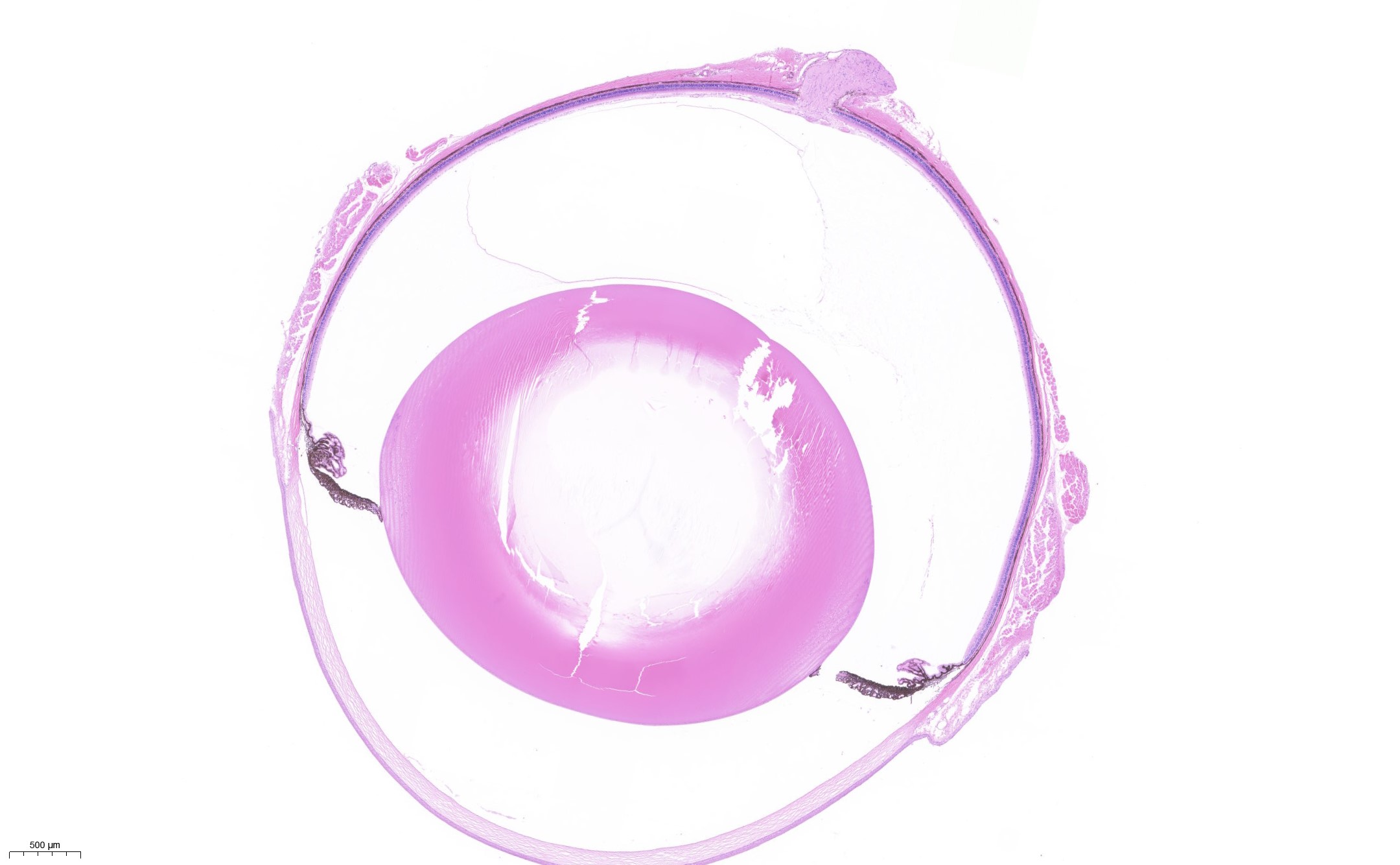

Supplement: Supplementary file 1 [file Data_Sheet_1.ZIP › Original data/Fig 1/HE-stained retina images/1.MNU/MNU-60 mg-1.jpg]

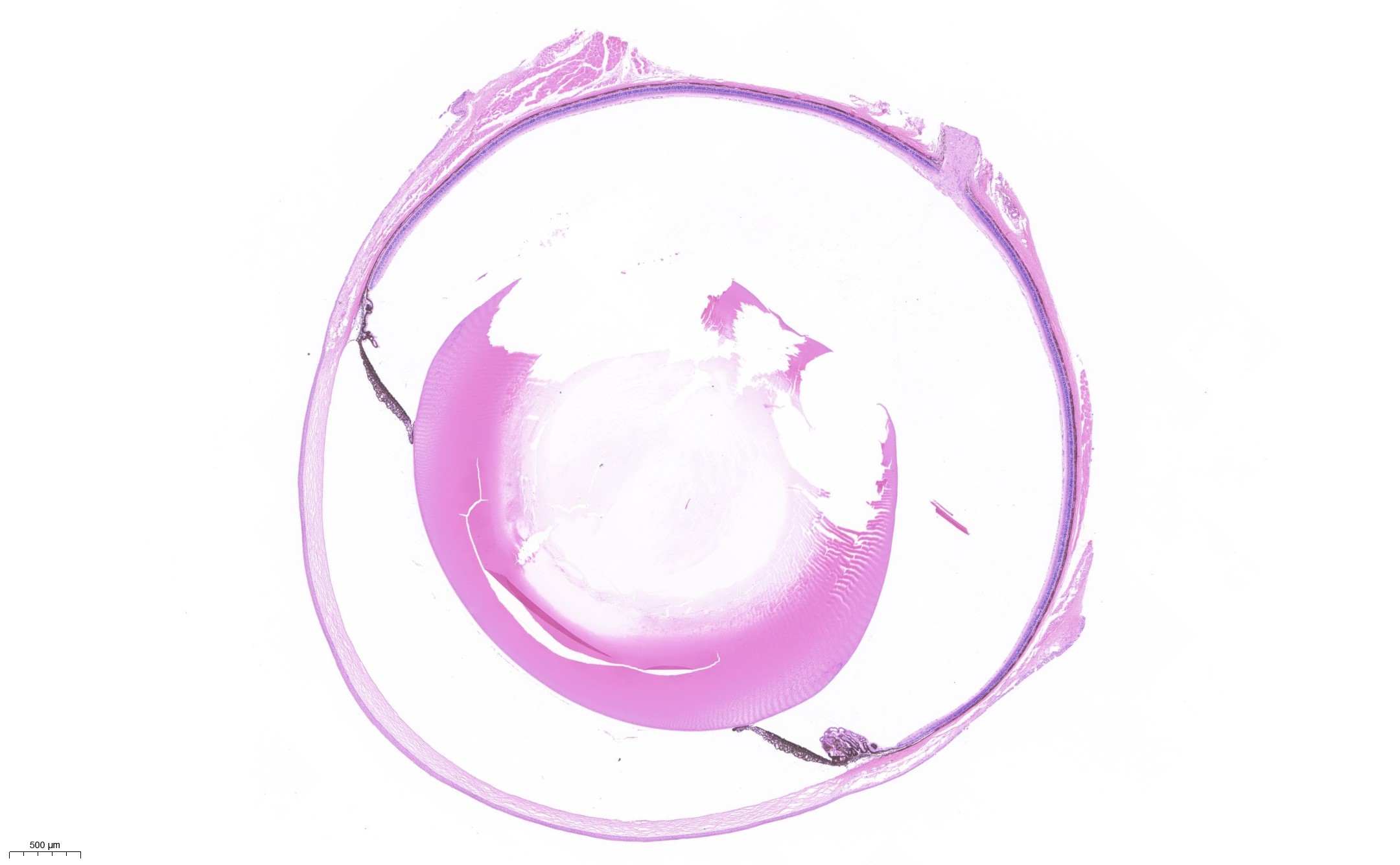

Supplement: Supplementary file 1 [file Data_Sheet_1.ZIP › Original data/Fig 1/HE-stained retina images/1.MNU/MNU-60 mg-2.jpg]

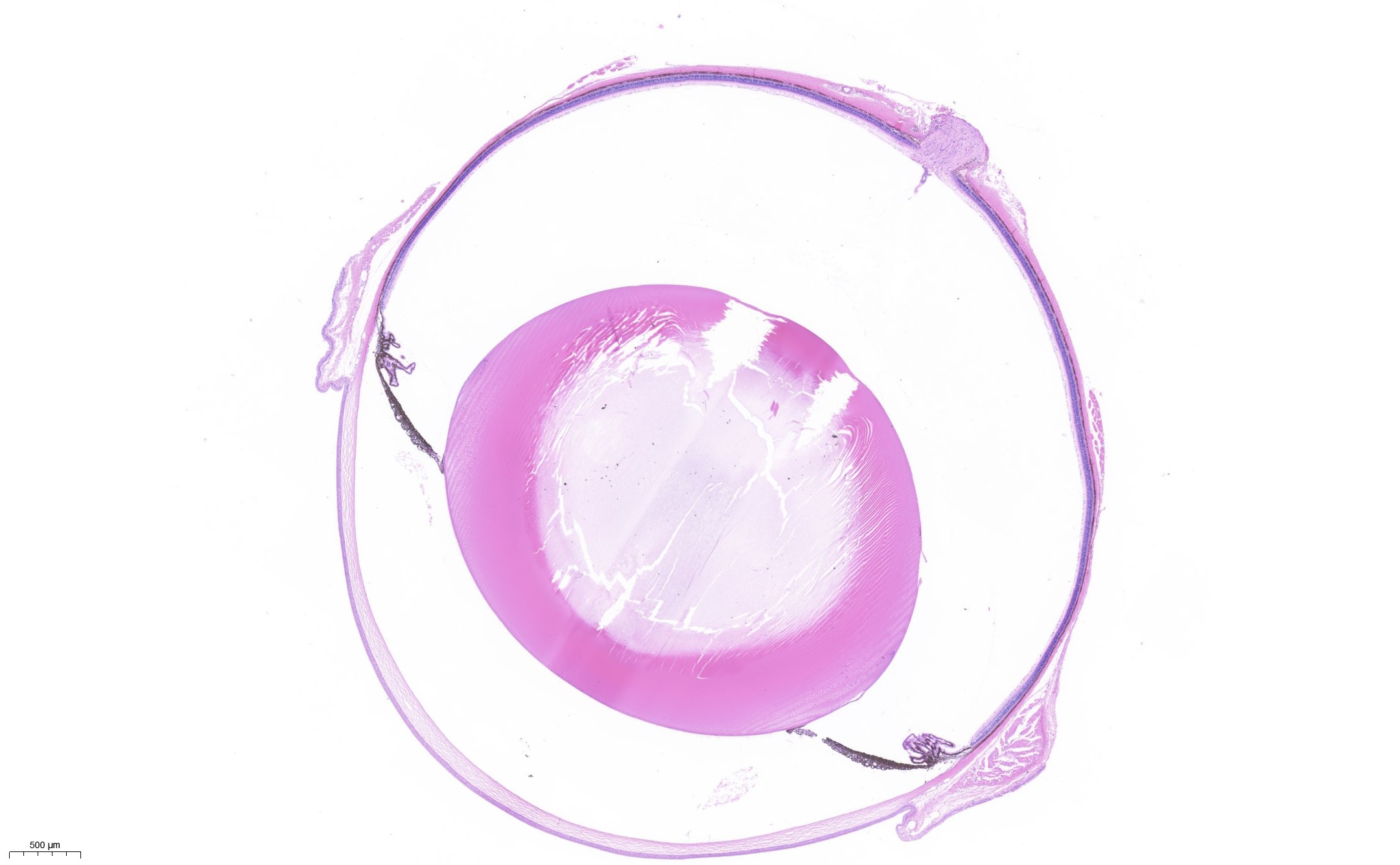

Supplement: Supplementary file 1 [file Data_Sheet_1.ZIP › Original data/Fig 1/HE-stained retina images/1.MNU/MNU-60 mg-3.jpg]

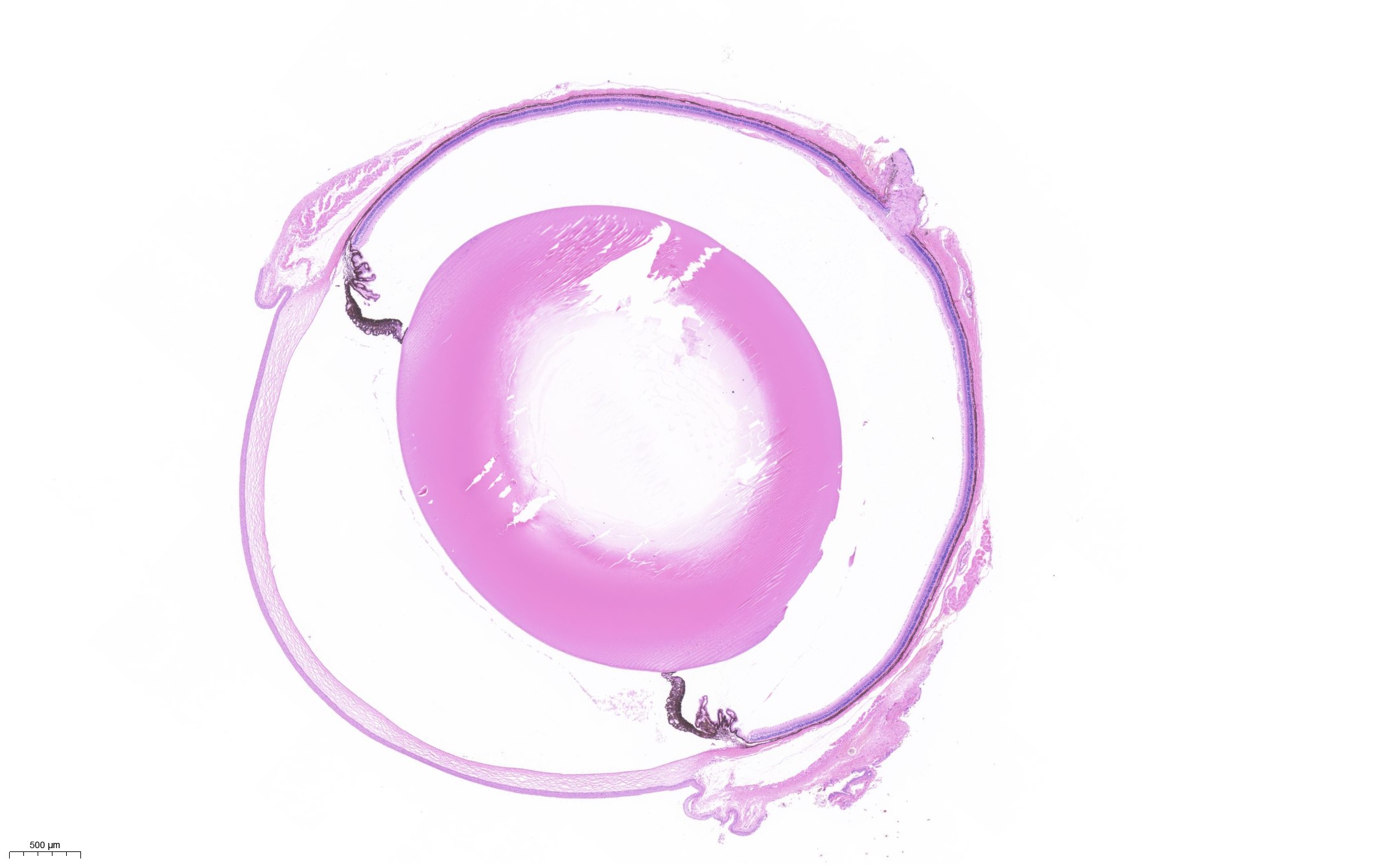

Supplement: Supplementary file 1 [file Data_Sheet_1.ZIP › Original data/Fig 1/HE-stained retina images/1.MNU/MNU-60 mg-4.jpg]

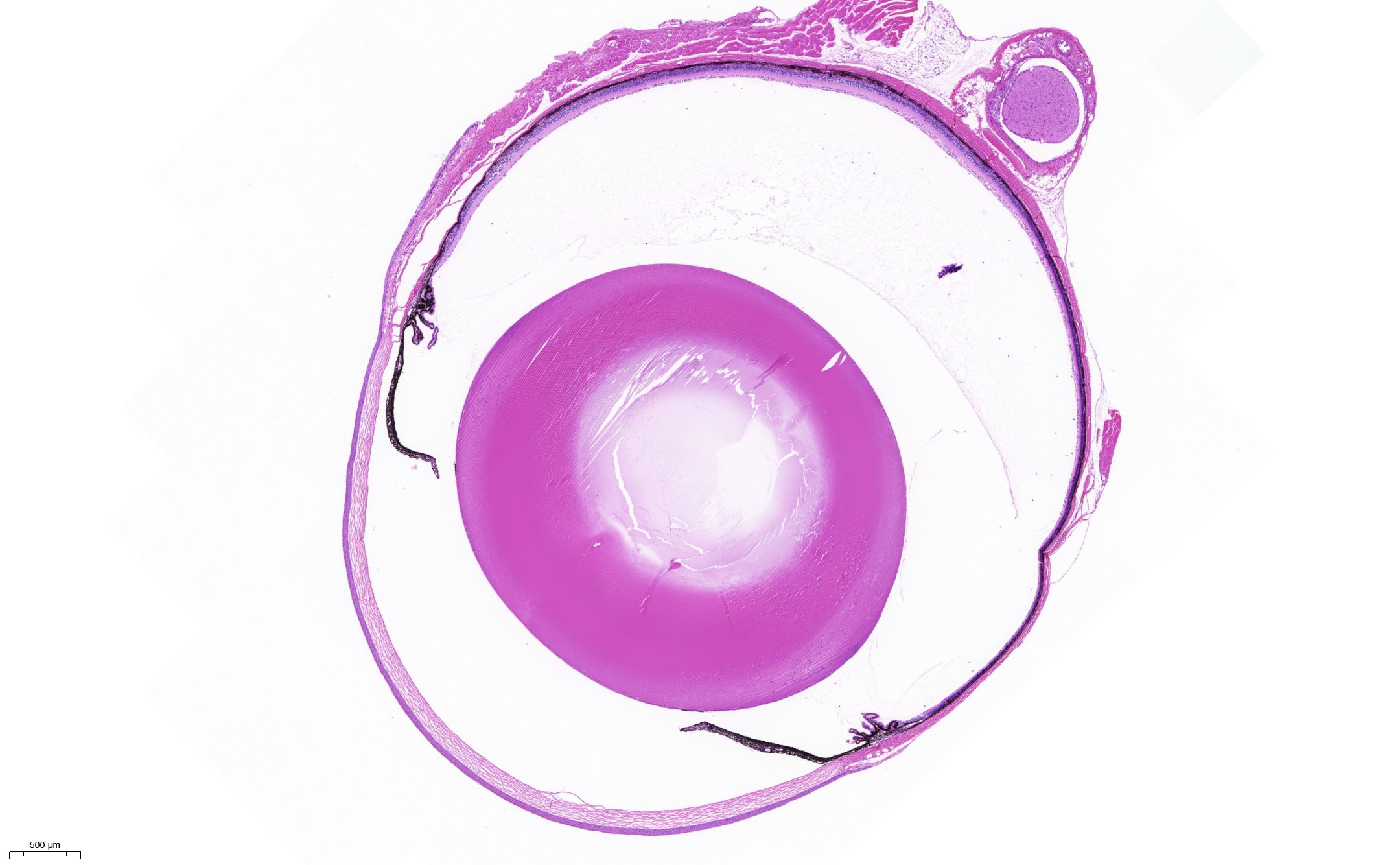

Supplement: Supplementary file 1 [file Data_Sheet_1.ZIP › Original data/Fig 1/HE-stained retina images/1.MNU/MNU-7 Day-1.jpg]

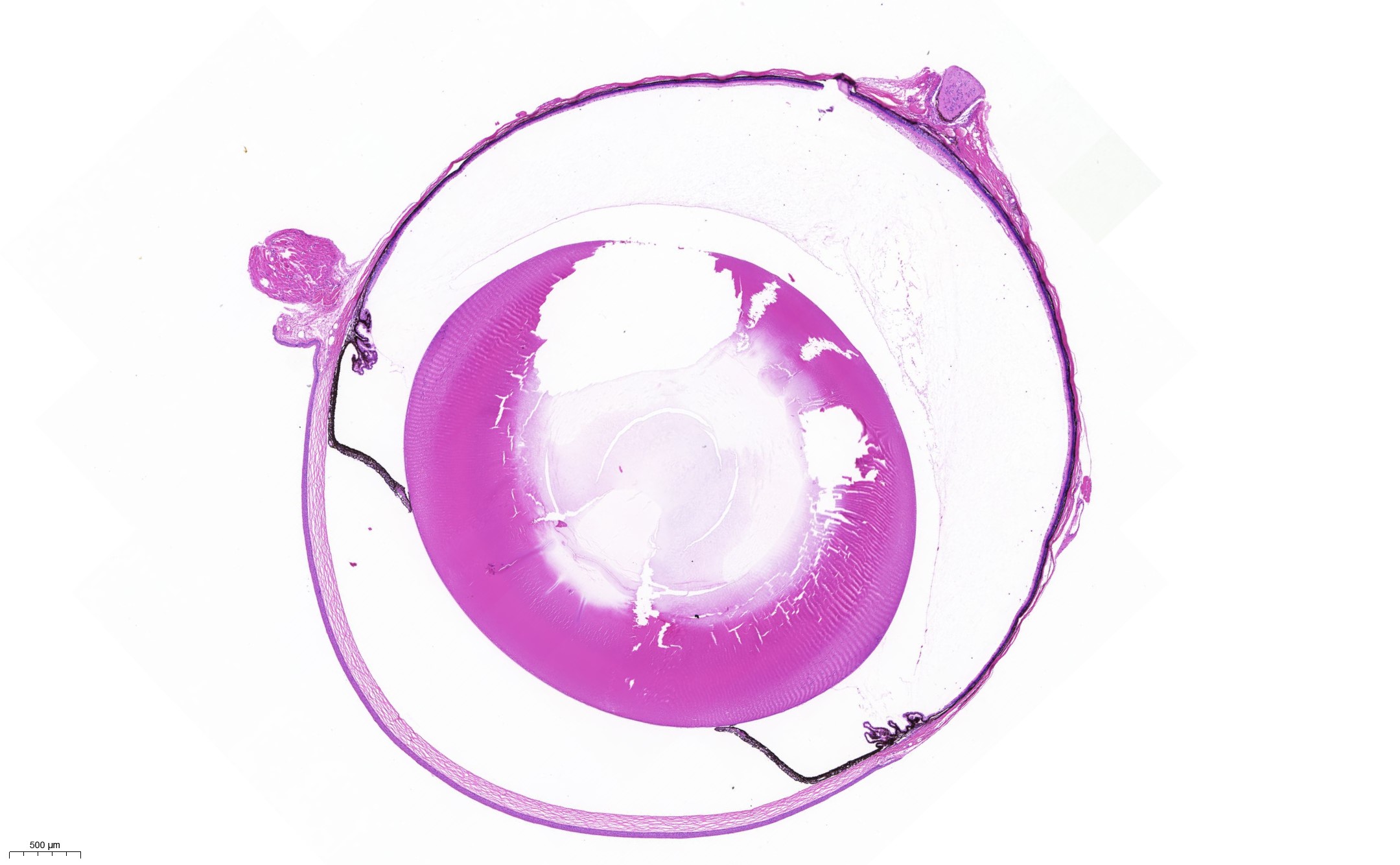

Supplement: Supplementary file 1 [file Data_Sheet_1.ZIP › Original data/Fig 1/HE-stained retina images/1.MNU/MNU-7 Day-2.jpg]

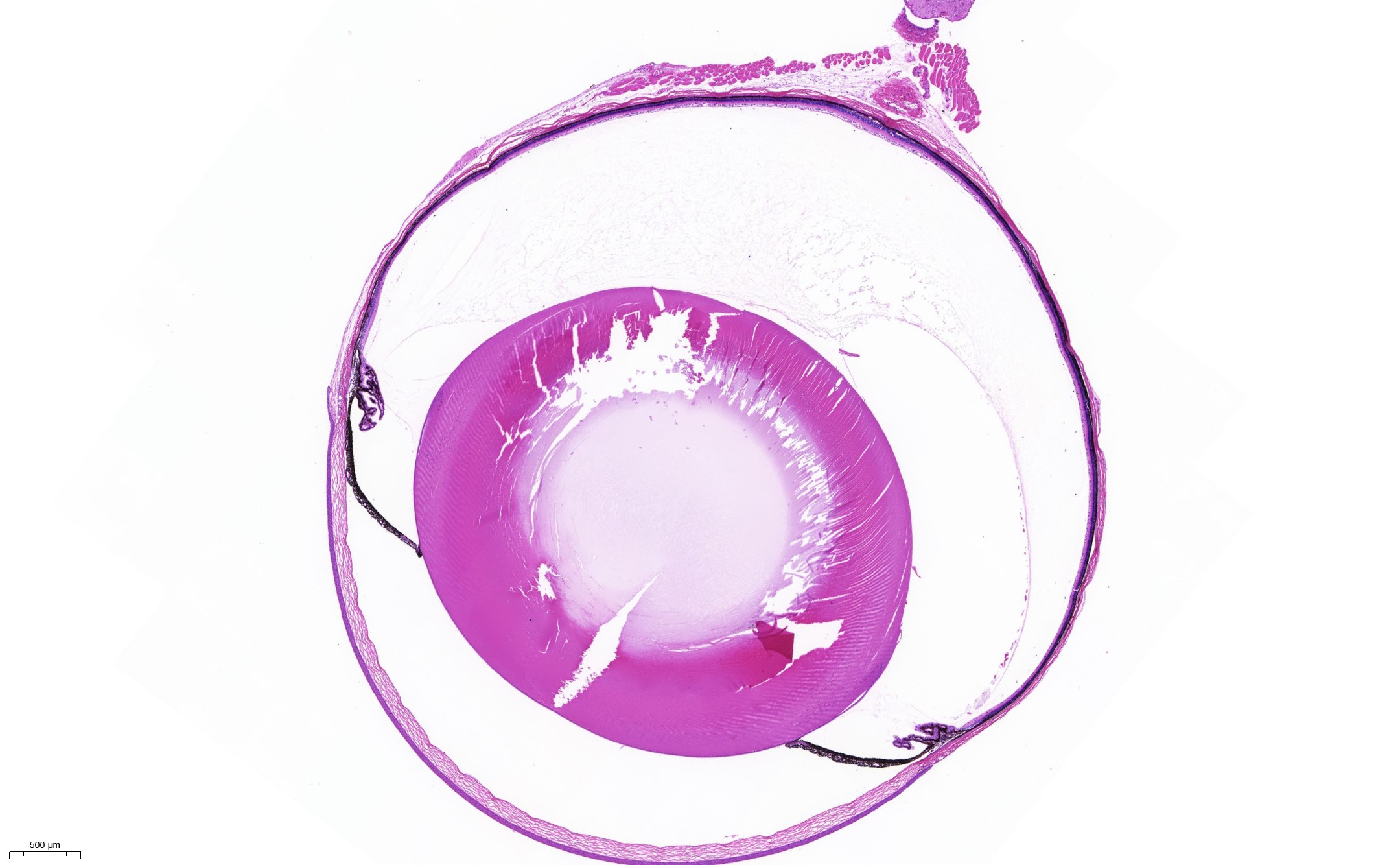

Supplement: Supplementary file 1 [file Data_Sheet_1.ZIP › Original data/Fig 1/HE-stained retina images/1.MNU/MNU-7 Day-3.jpg]

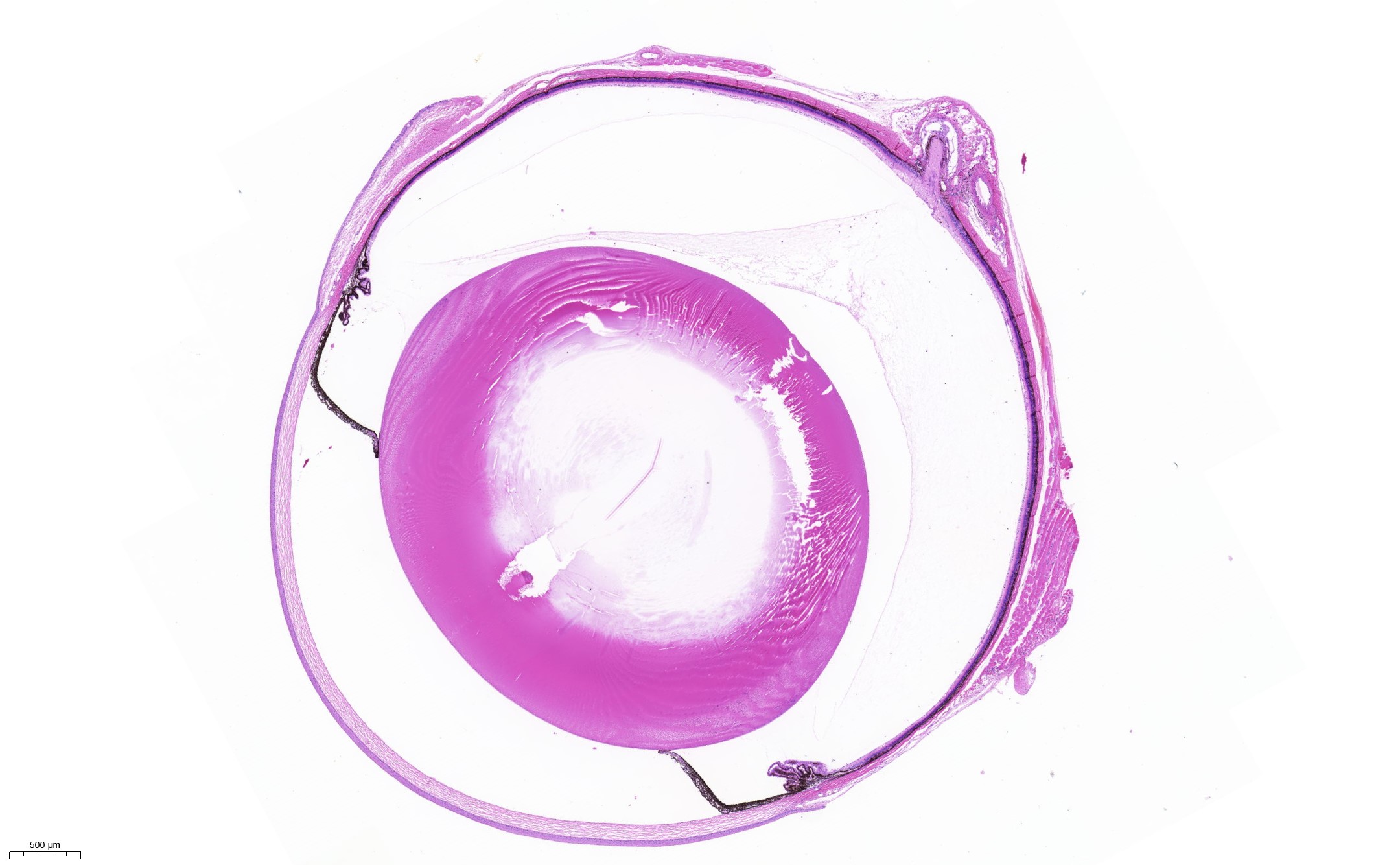

Supplement: Supplementary file 1 [file Data_Sheet_1.ZIP › Original data/Fig 1/HE-stained retina images/1.MNU/MNU-7 Day-4.jpg]

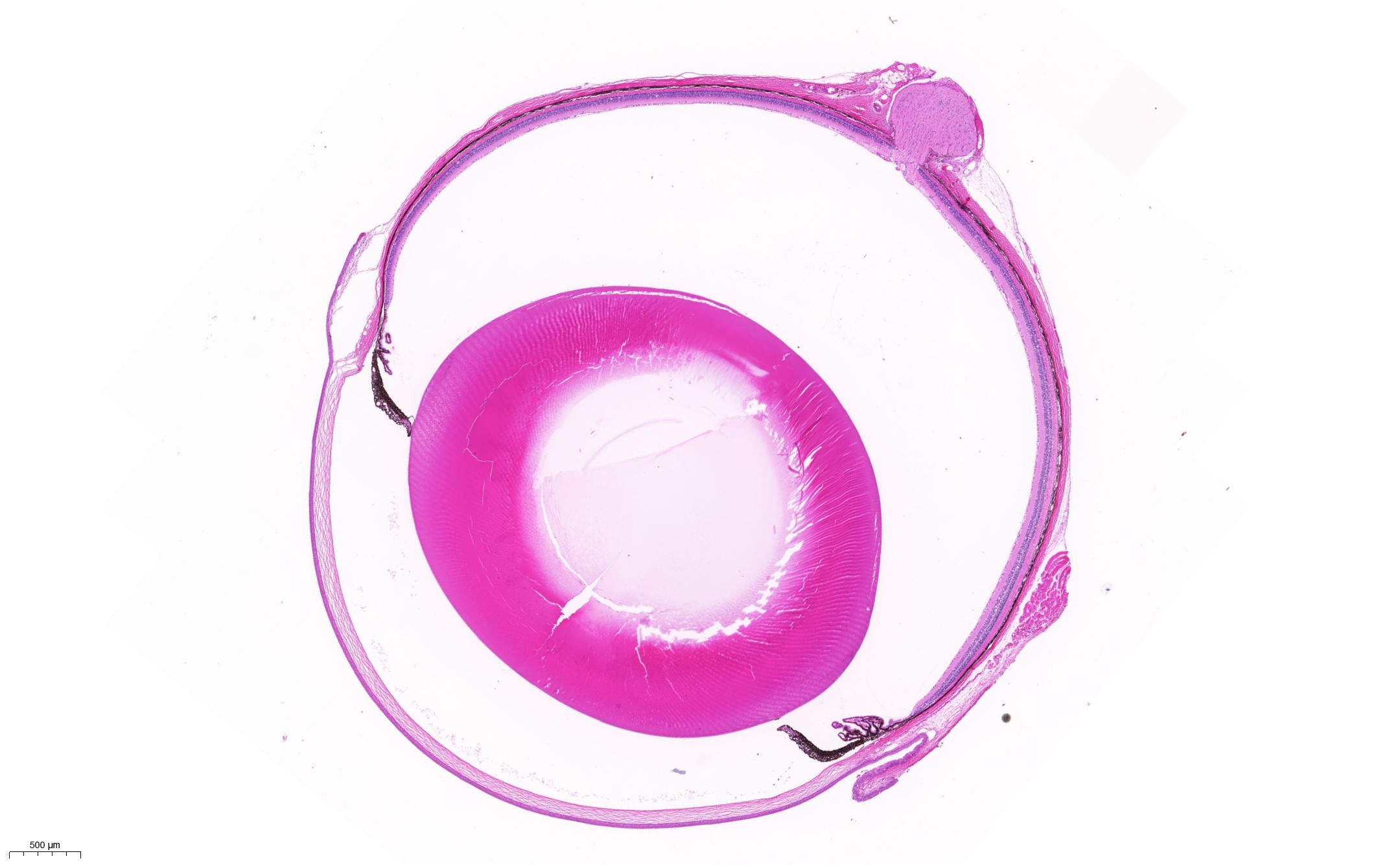

Supplement: Supplementary file 1 [file Data_Sheet_1.ZIP › Original data/Fig 1/HE-stained retina images/2.RCS/RCS-1.jpg]

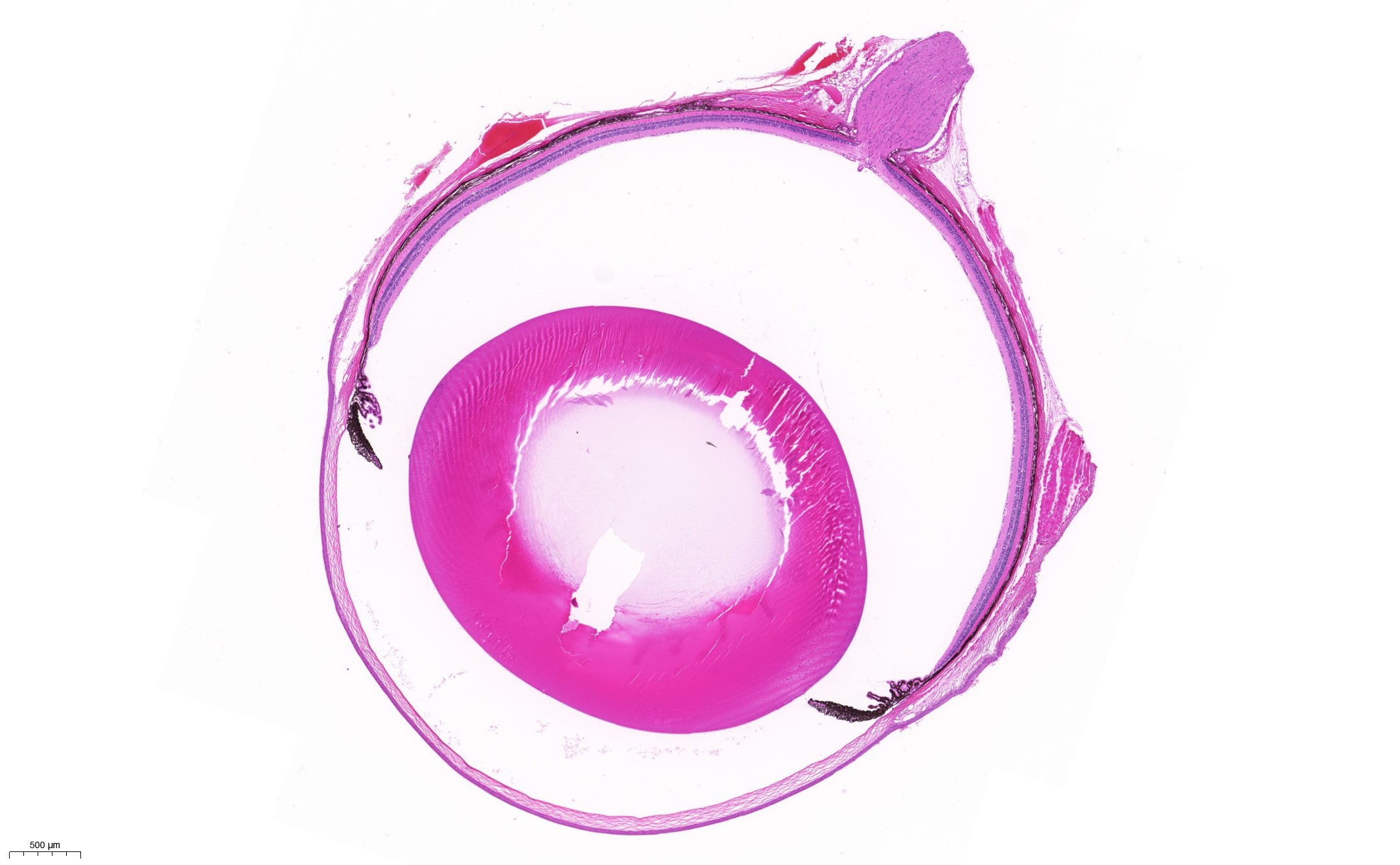

Supplement: Supplementary file 1 [file Data_Sheet_1.ZIP › Original data/Fig 1/HE-stained retina images/2.RCS/RCS-2.jpg]

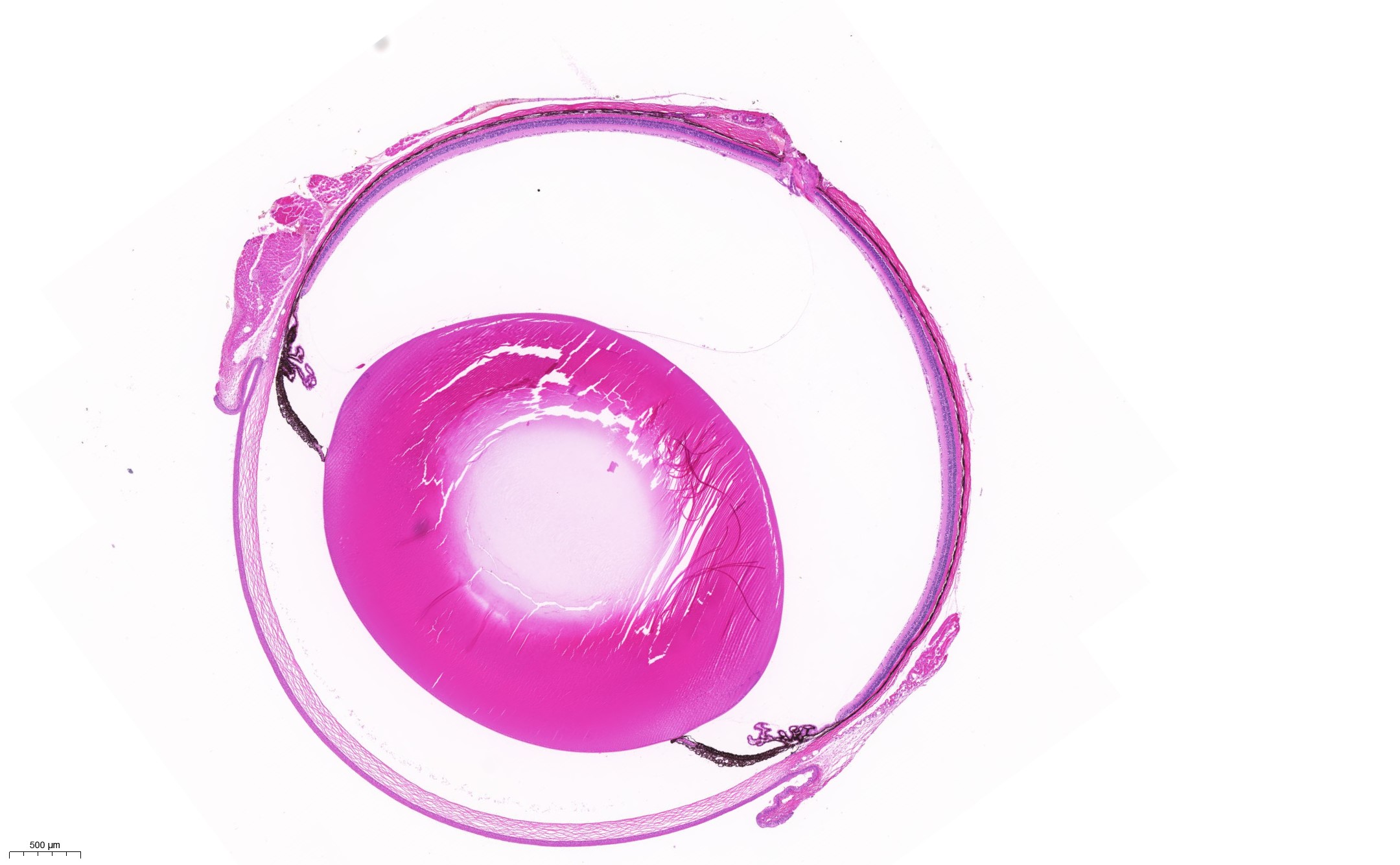

Supplement: Supplementary file 1 [file Data_Sheet_1.ZIP › Original data/Fig 1/HE-stained retina images/2.RCS/RCS-3.jpg]

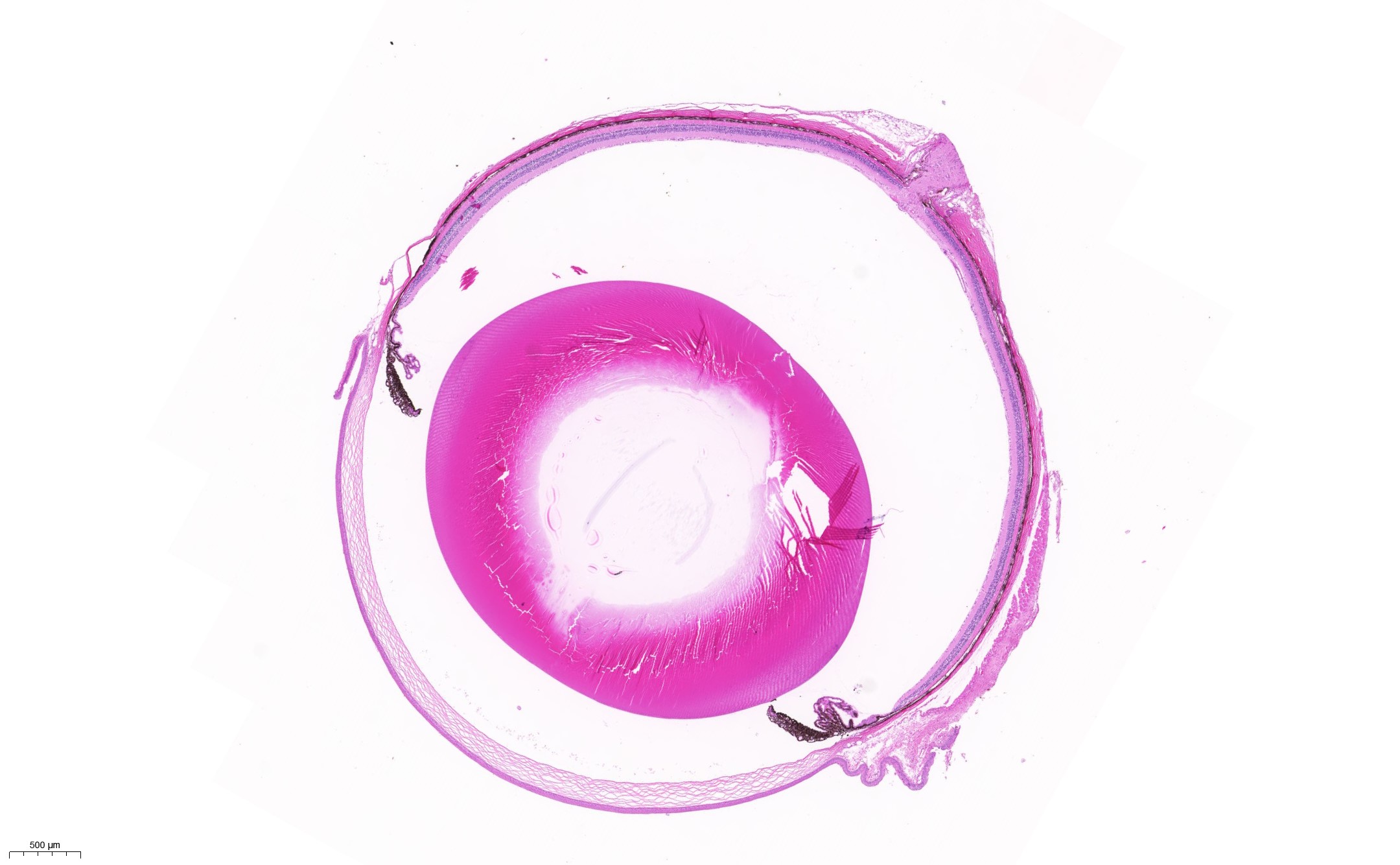

Supplement: Supplementary file 1 [file Data_Sheet_1.ZIP › Original data/Fig 1/HE-stained retina images/2.RCS/RCS-4.jpg]

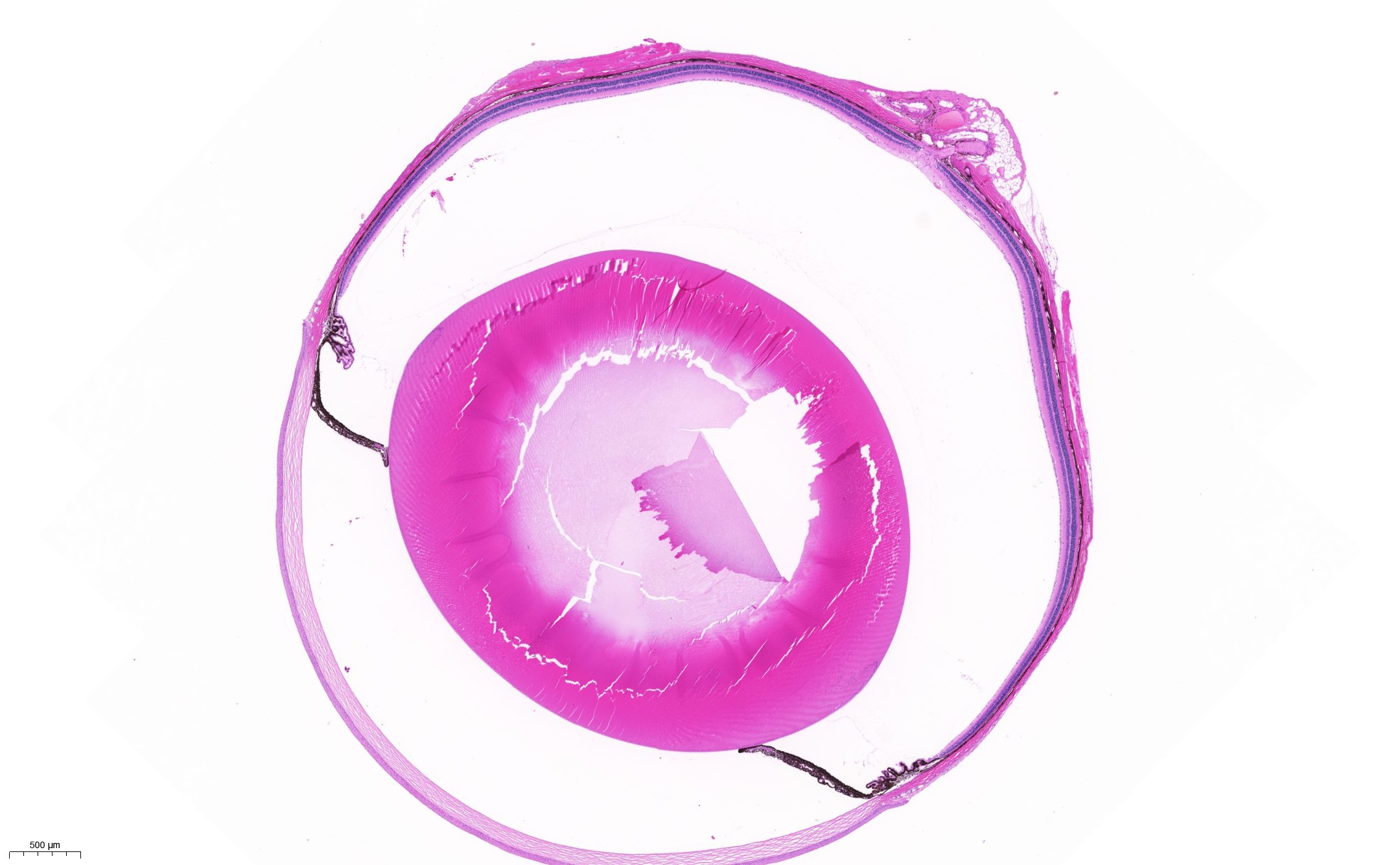

Supplement: Supplementary file 1 [file Data_Sheet_1.ZIP › Original data/Fig 1/HE-stained retina images/3.RDY/RDY-1.jpg]

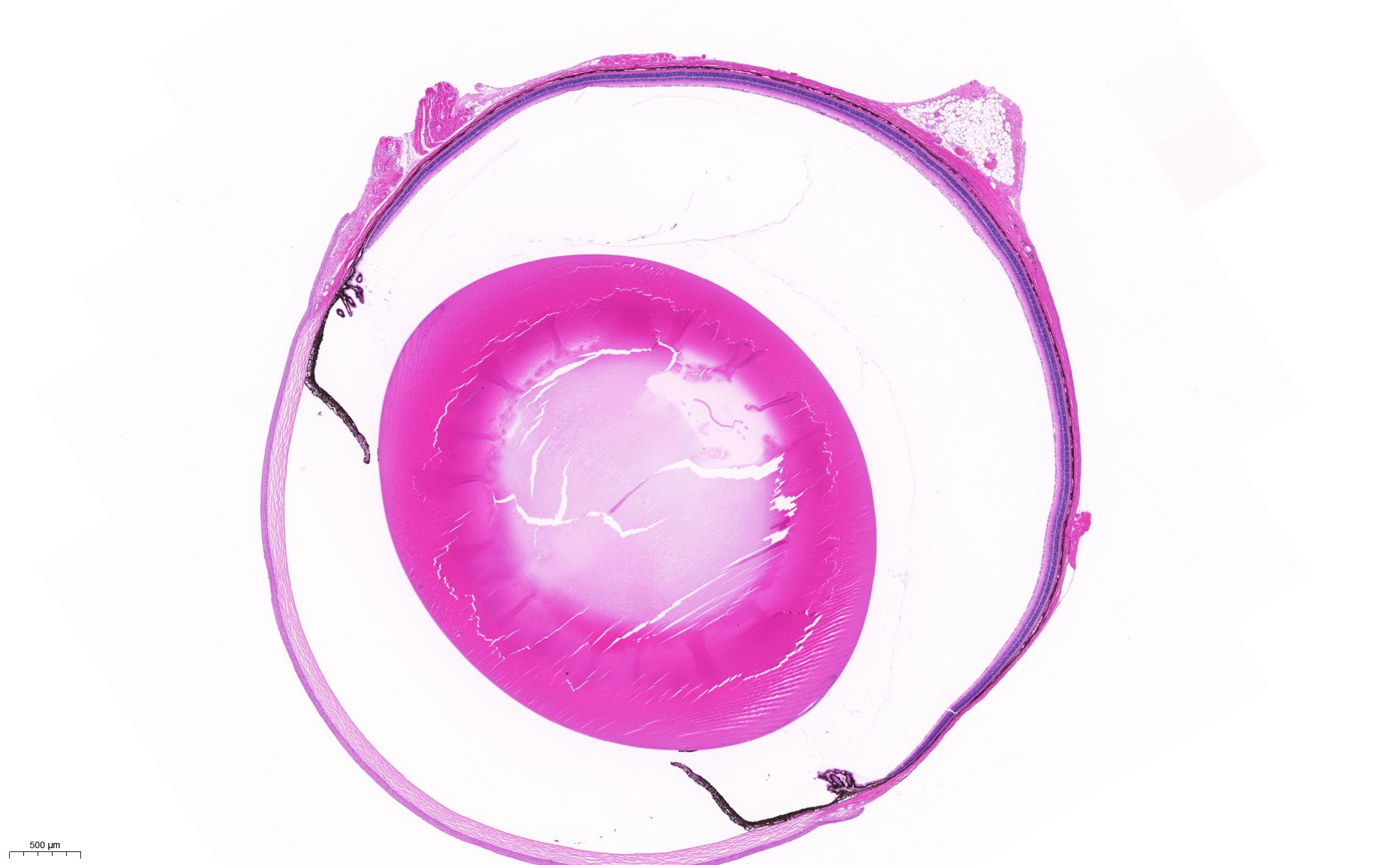

Supplement: Supplementary file 1 [file Data_Sheet_1.ZIP › Original data/Fig 1/HE-stained retina images/3.RDY/RDY-2.jpg]

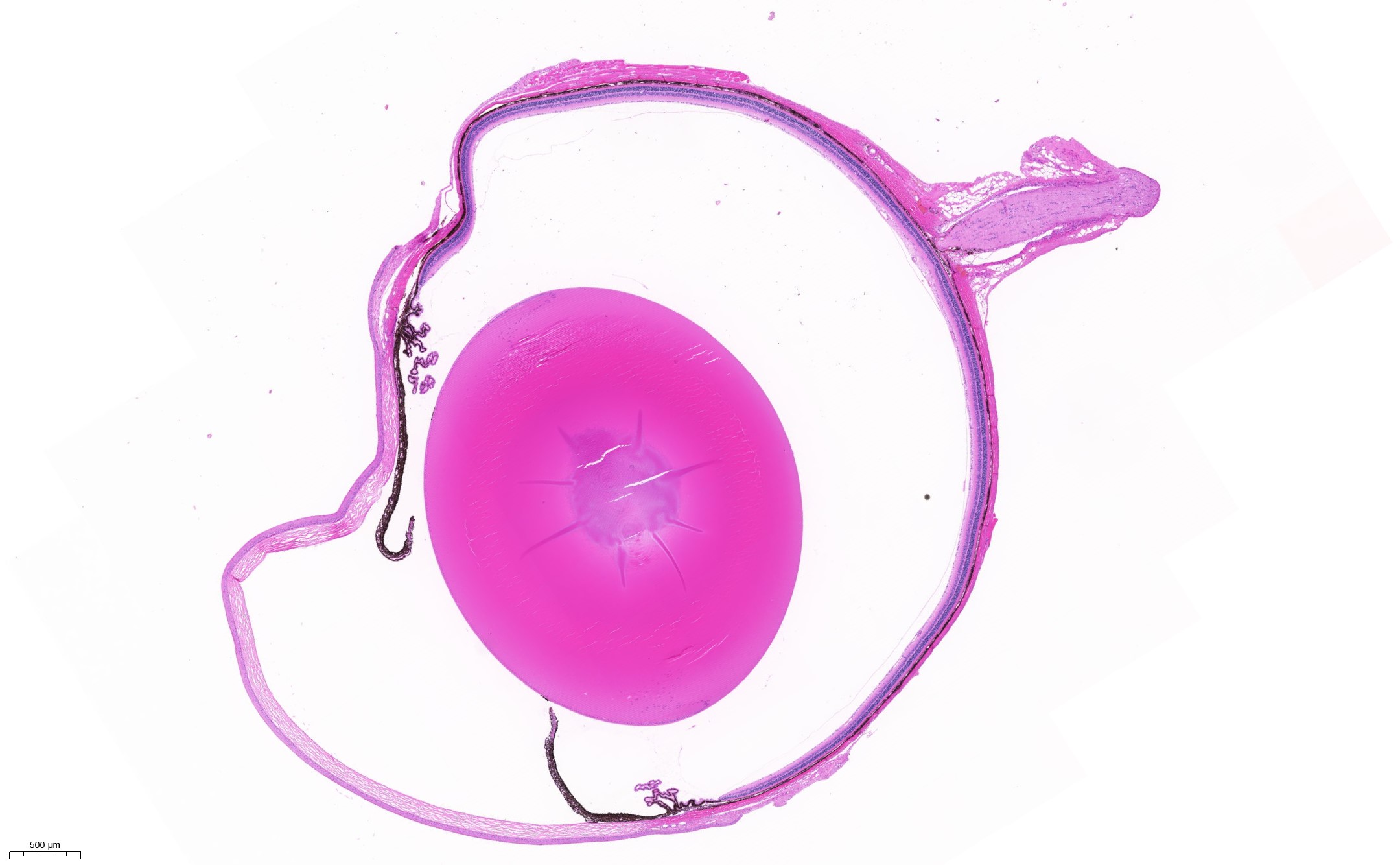

Supplement: Supplementary file 1 [file Data_Sheet_1.ZIP › Original data/Fig 1/HE-stained retina images/3.RDY/RDY-3.jpg]

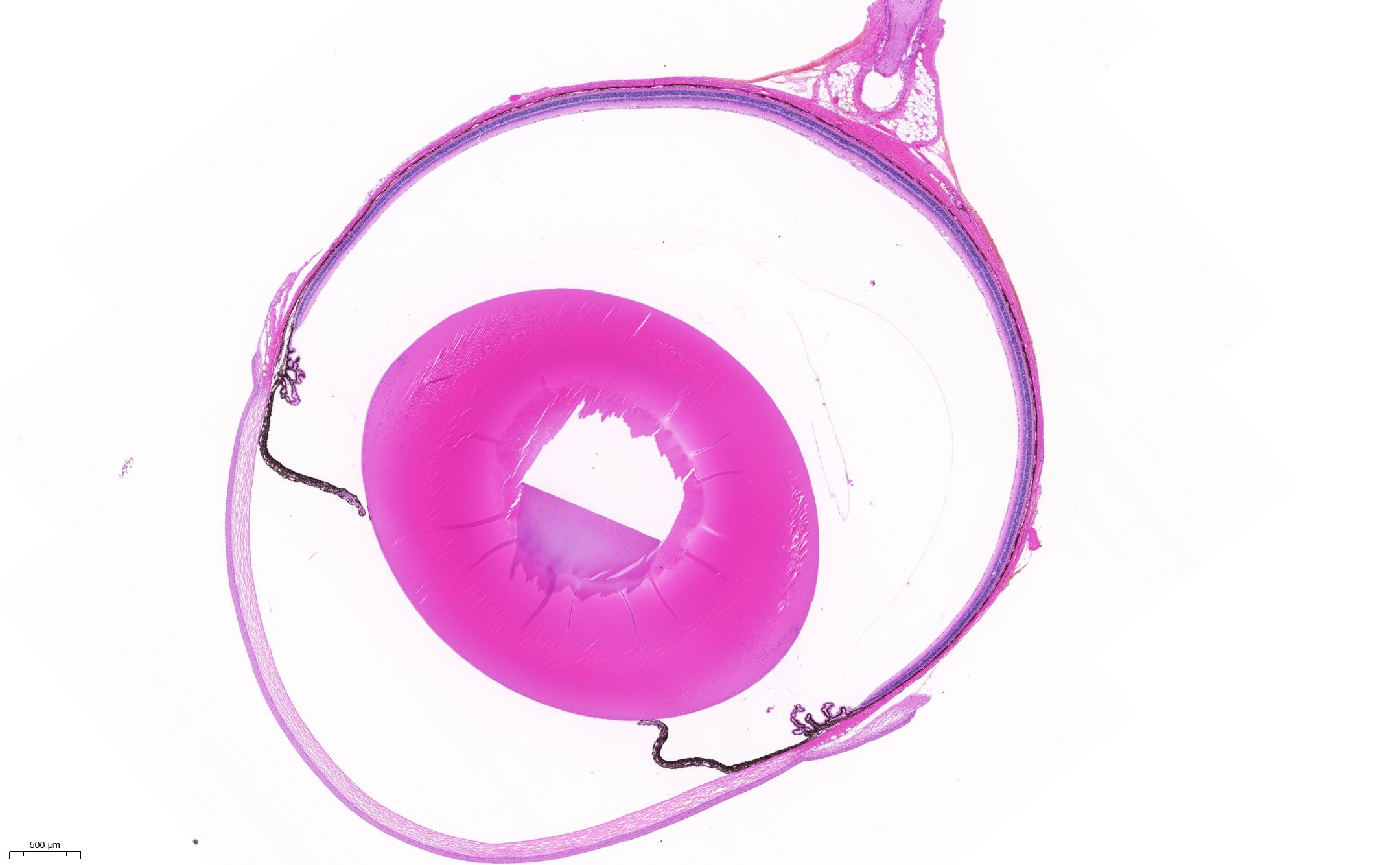

Supplement: Supplementary file 1 [file Data_Sheet_1.ZIP › Original data/Fig 1/HE-stained retina images/3.RDY/RDY-4.jpg]

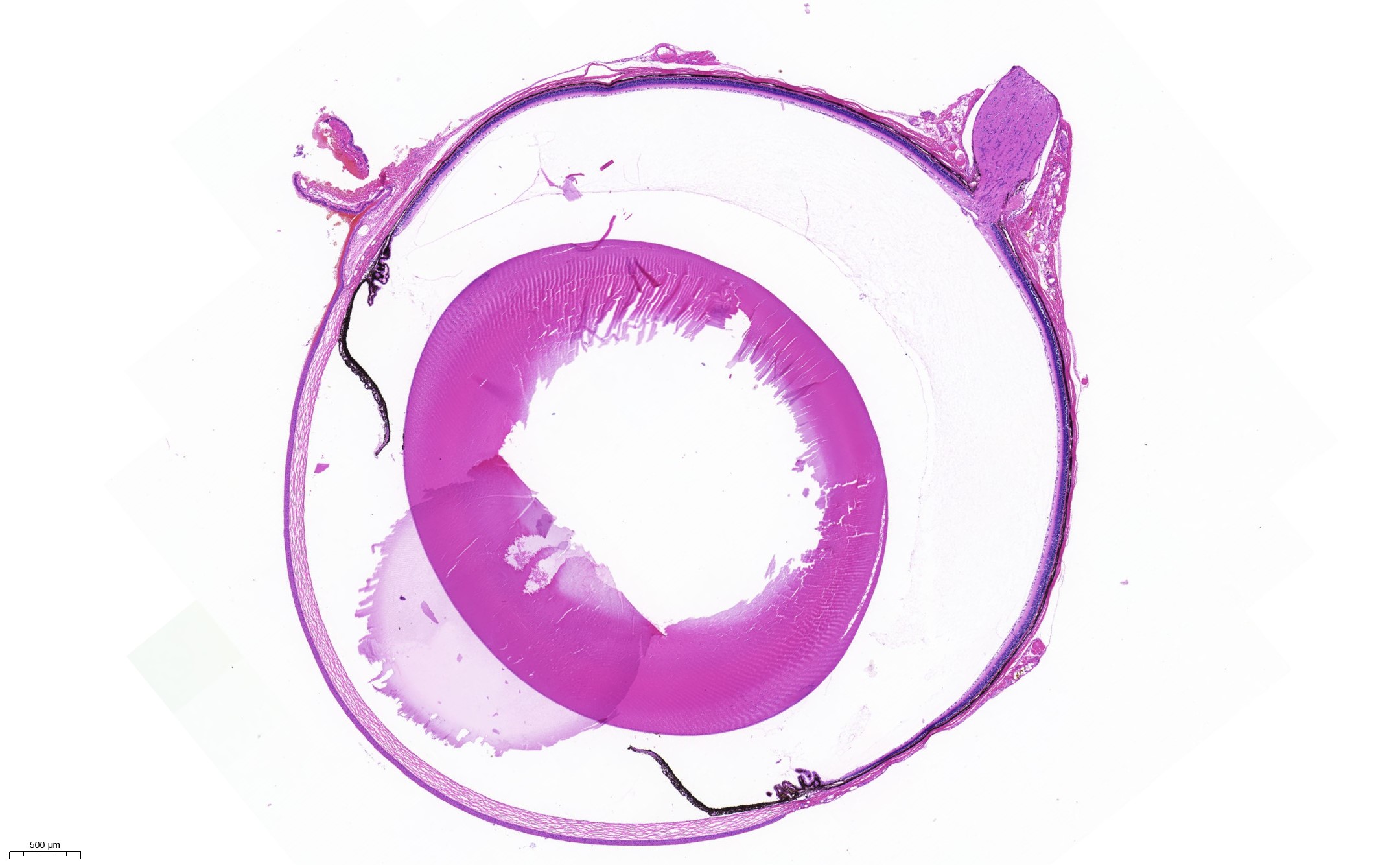

Supplement: Supplementary file 1 [file Data_Sheet_1.ZIP › Original data/Fig 1/HE-stained retina images/4.Representative figure/MNU.jpg]

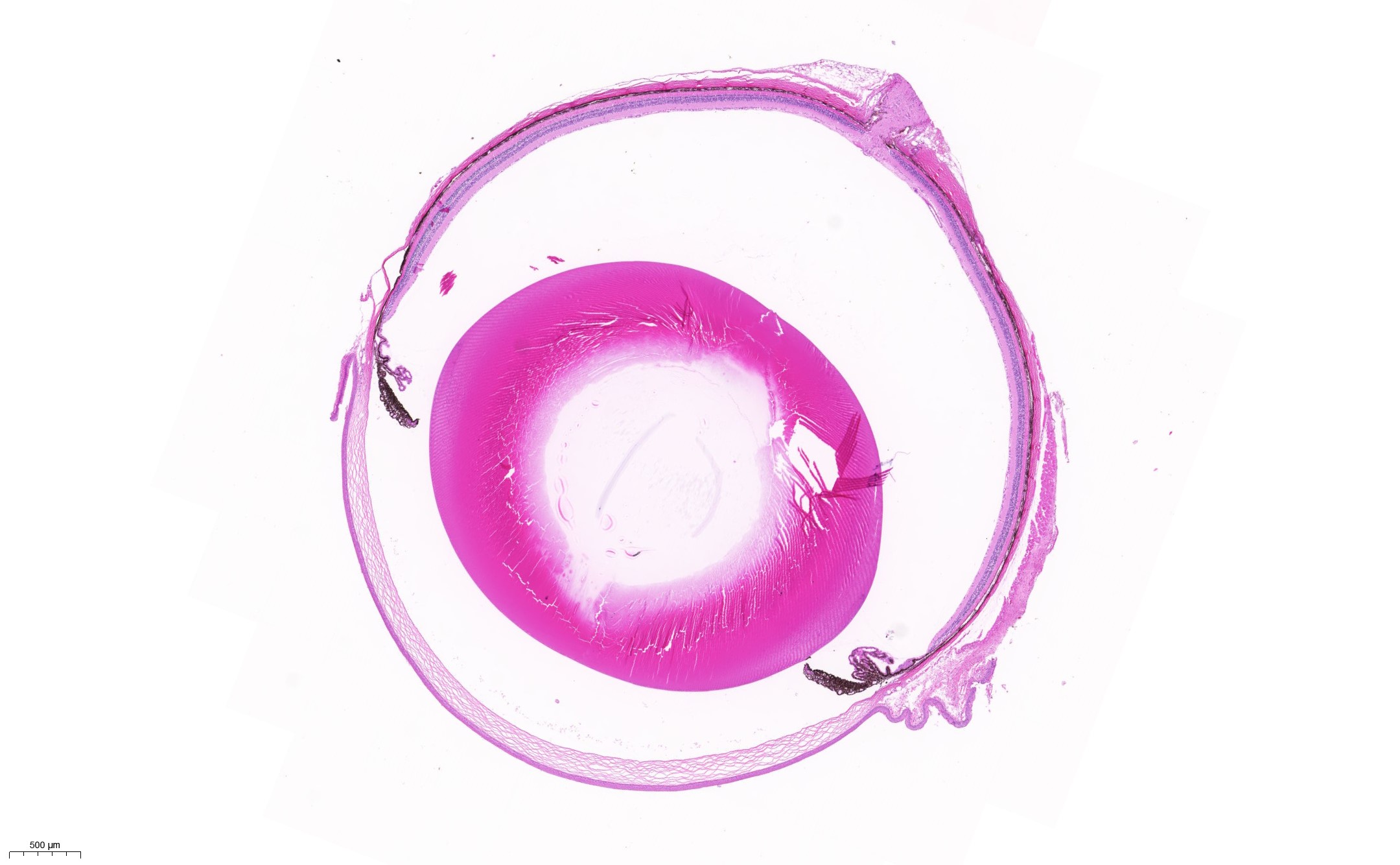

Supplement: Supplementary file 1 [file Data_Sheet_1.ZIP › Original data/Fig 1/HE-stained retina images/4.Representative figure/RCS.jpg]

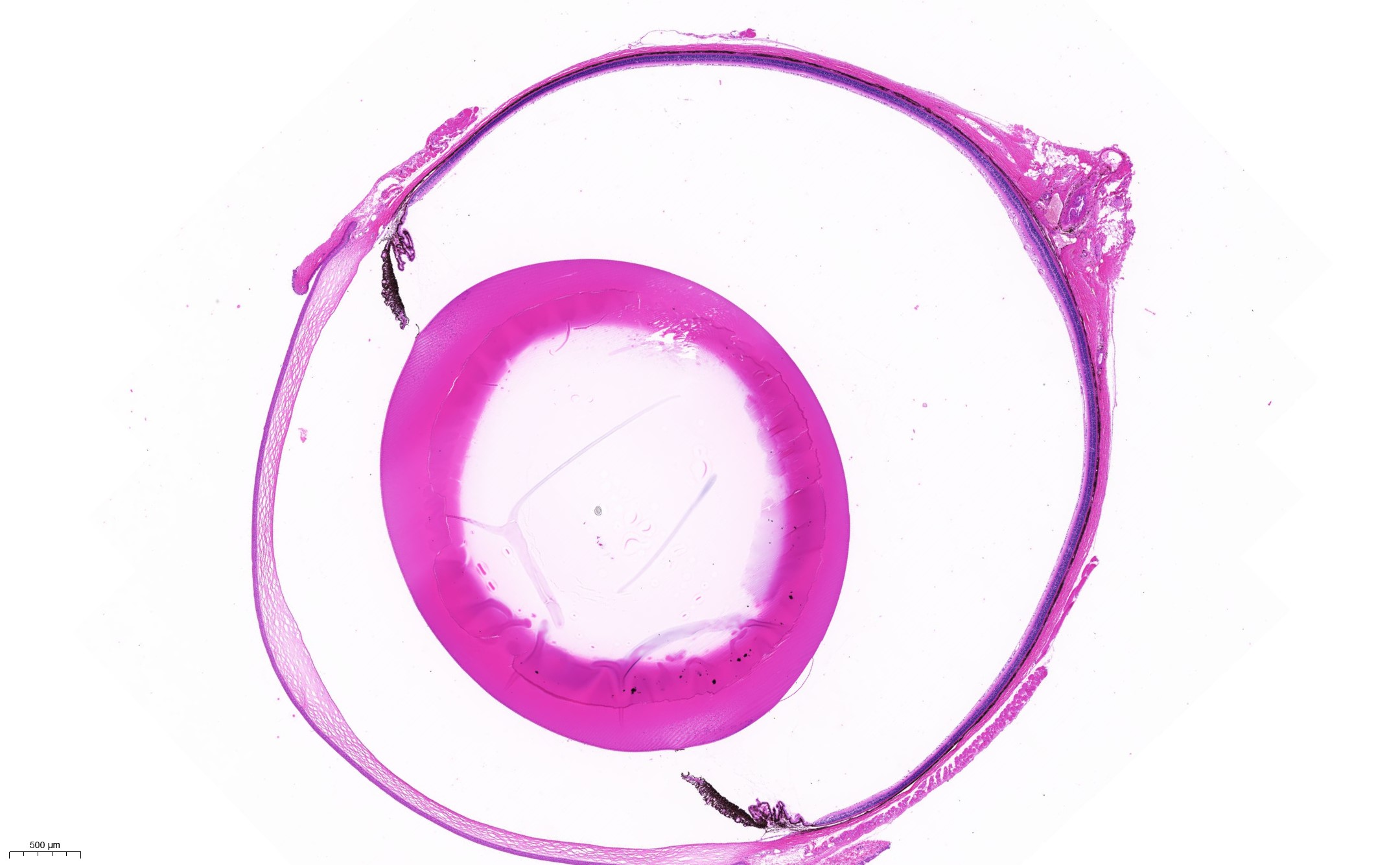

Supplement: Supplementary file 1 [file Data_Sheet_1.ZIP › Original data/Fig 1/HE-stained retina images/4.Representative figure/RDY.jpg]

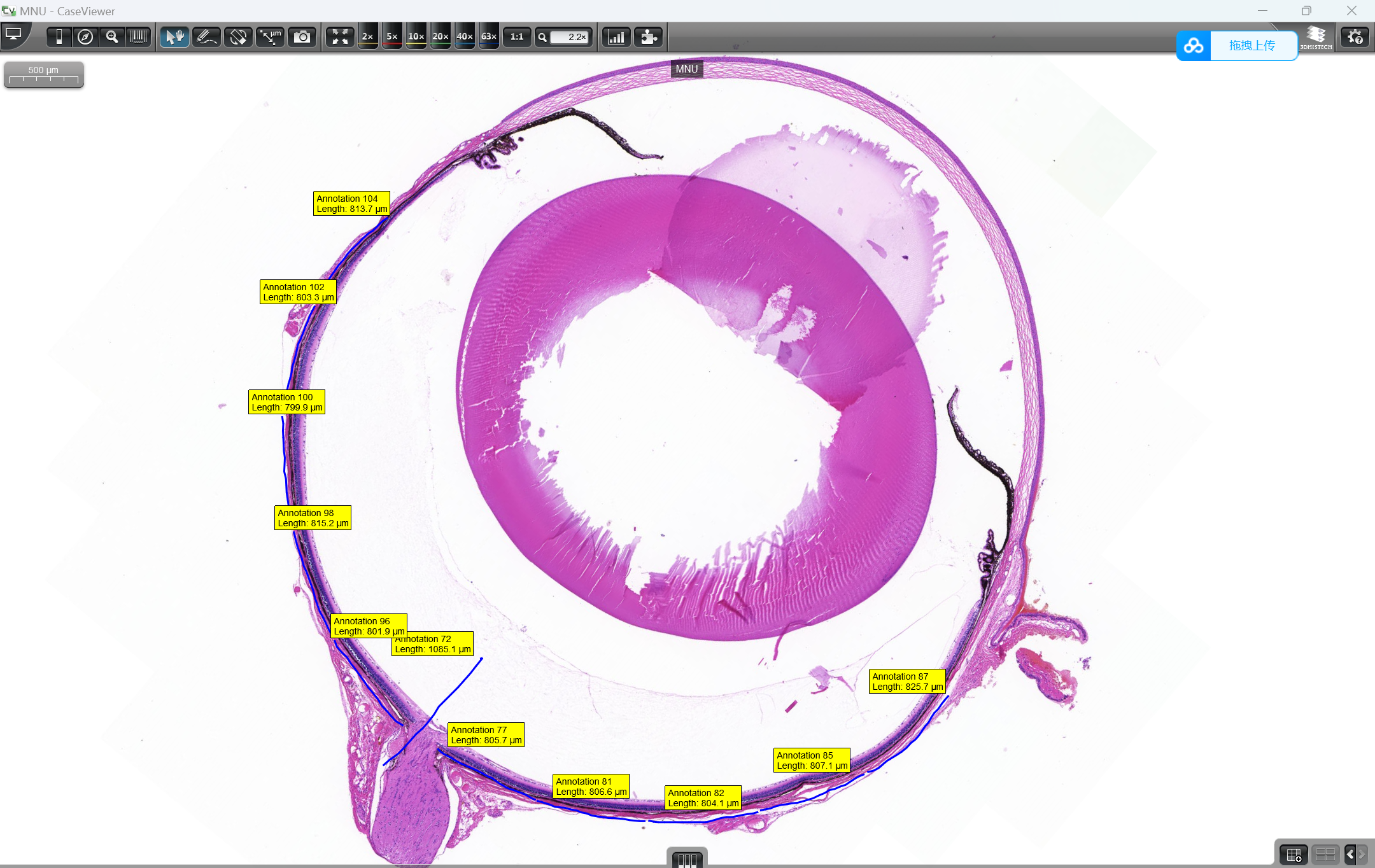

Supplement: Supplementary file 1 [file Data_Sheet_1.ZIP › Original data/Fig 1/HE-stained retina images/Figure 1. ONL thinckness measurement method.png]

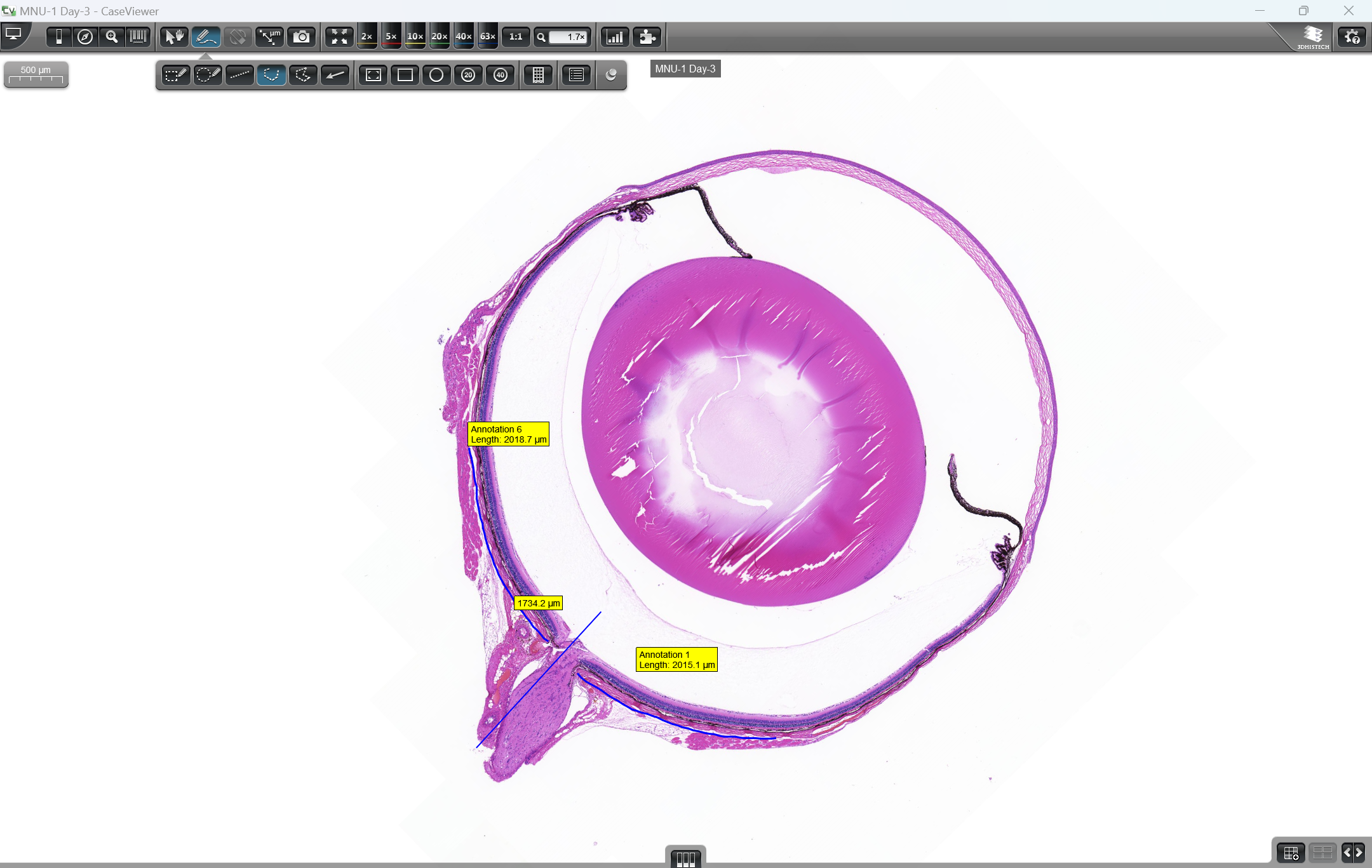

Supplement: Supplementary file 1 [file Data_Sheet_1.ZIP › Original data/Fig 1/HE-stained retina images/Figure 2. ONL cell number measurement method.png]

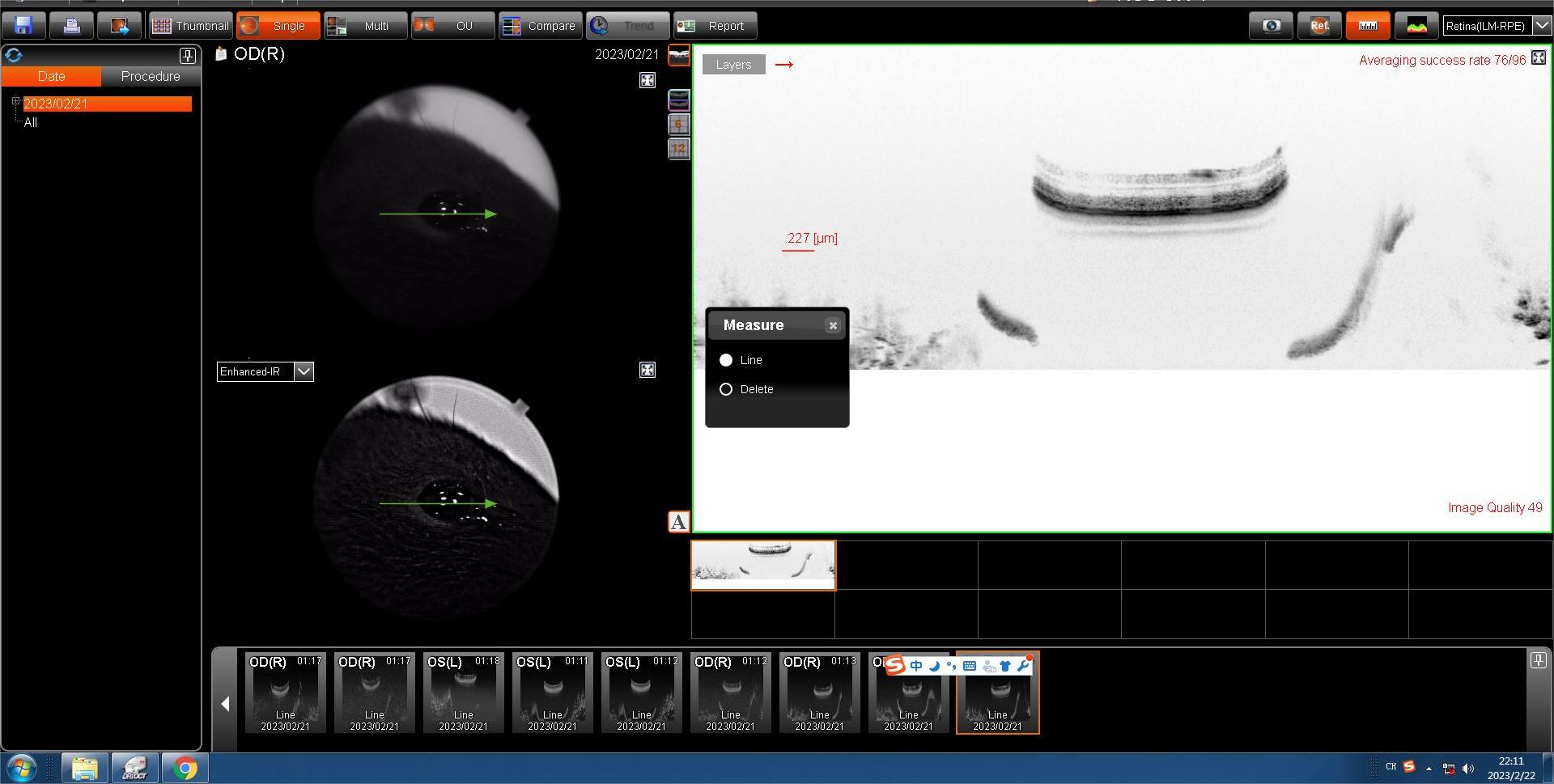

Supplement: Supplementary file 1 [file Data_Sheet_1.ZIP › Original data/Fig 1/OCT images/OCT images/1.MNU/MNU-1.JPG]

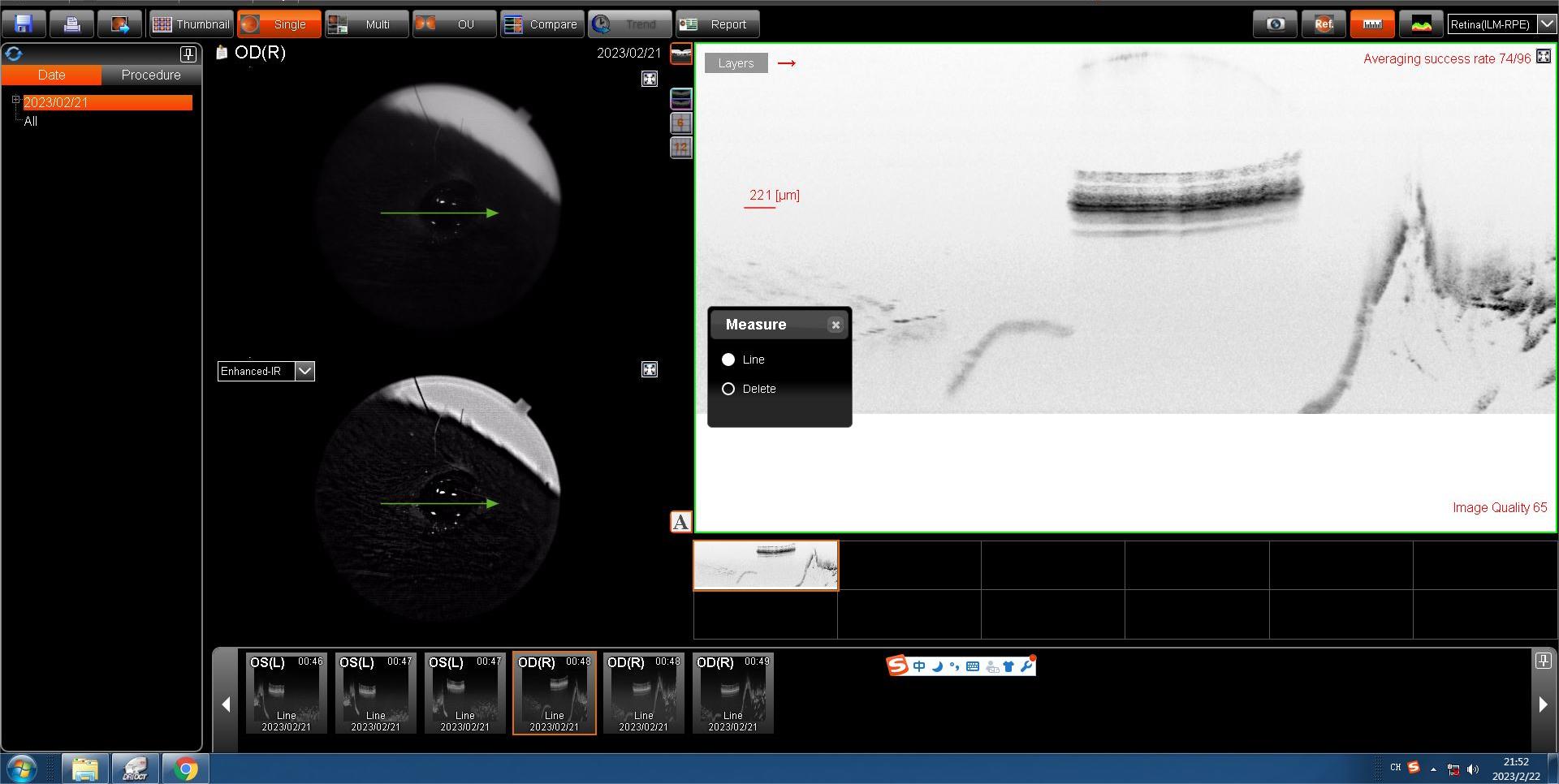

Supplement: Supplementary file 1 [file Data_Sheet_1.ZIP › Original data/Fig 1/OCT images/OCT images/1.MNU/MNU-2.JPG]

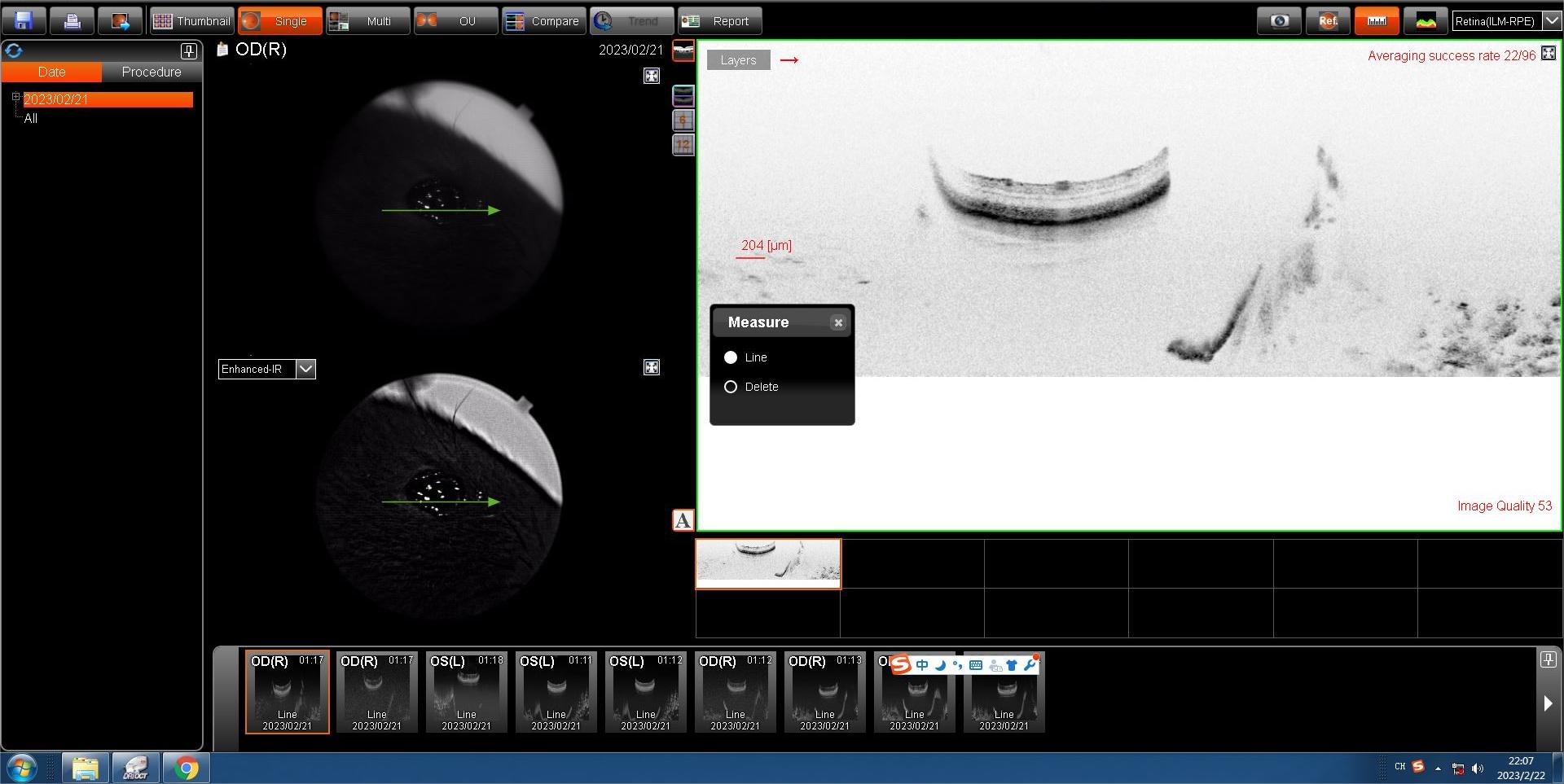

Supplement: Supplementary file 1 [file Data_Sheet_1.ZIP › Original data/Fig 1/OCT images/OCT images/1.MNU/MNU-3.JPG]

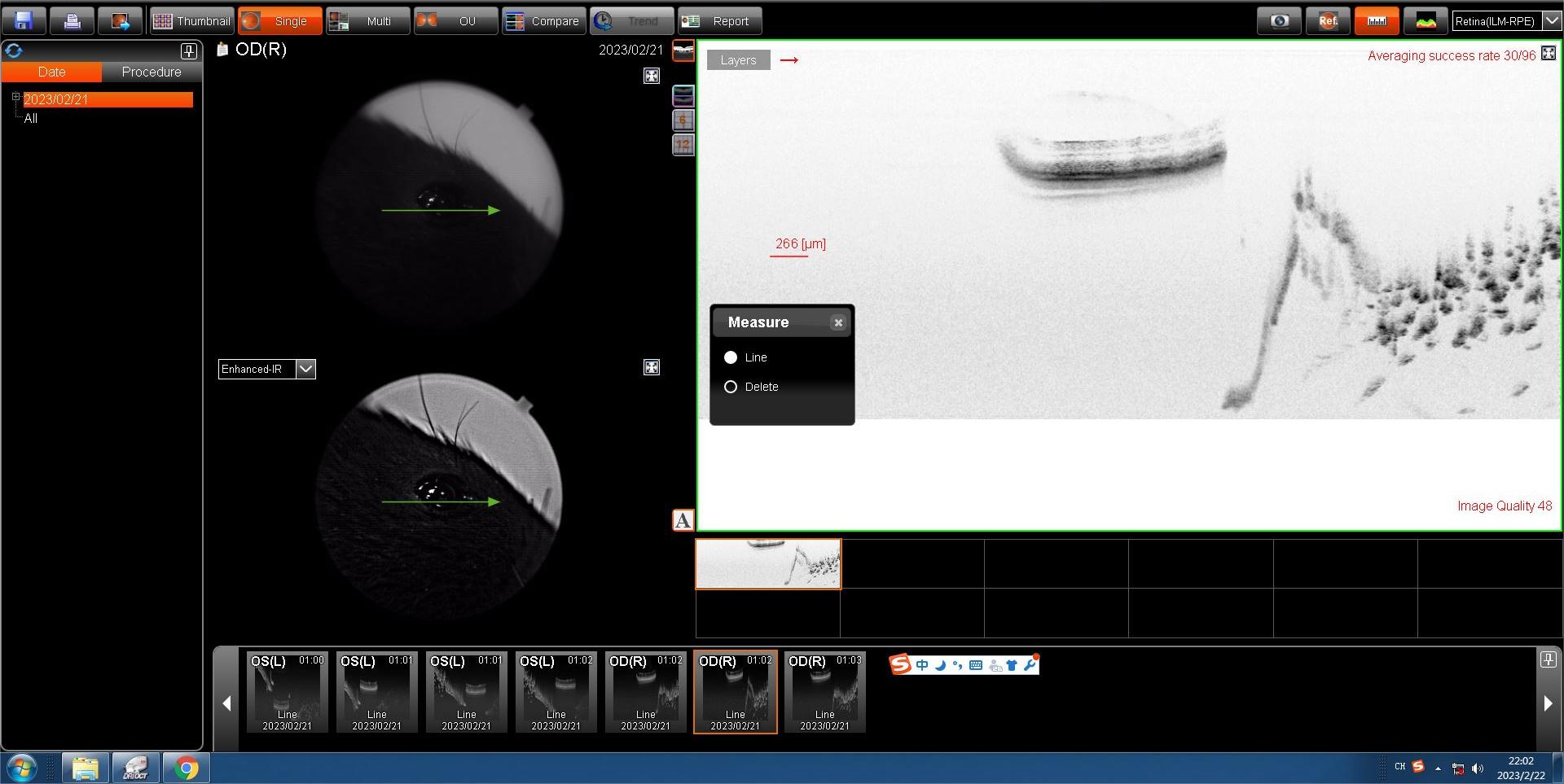

Supplement: Supplementary file 1 [file Data_Sheet_1.ZIP › Original data/Fig 1/OCT images/OCT images/1.MNU/MNU-4.JPG]

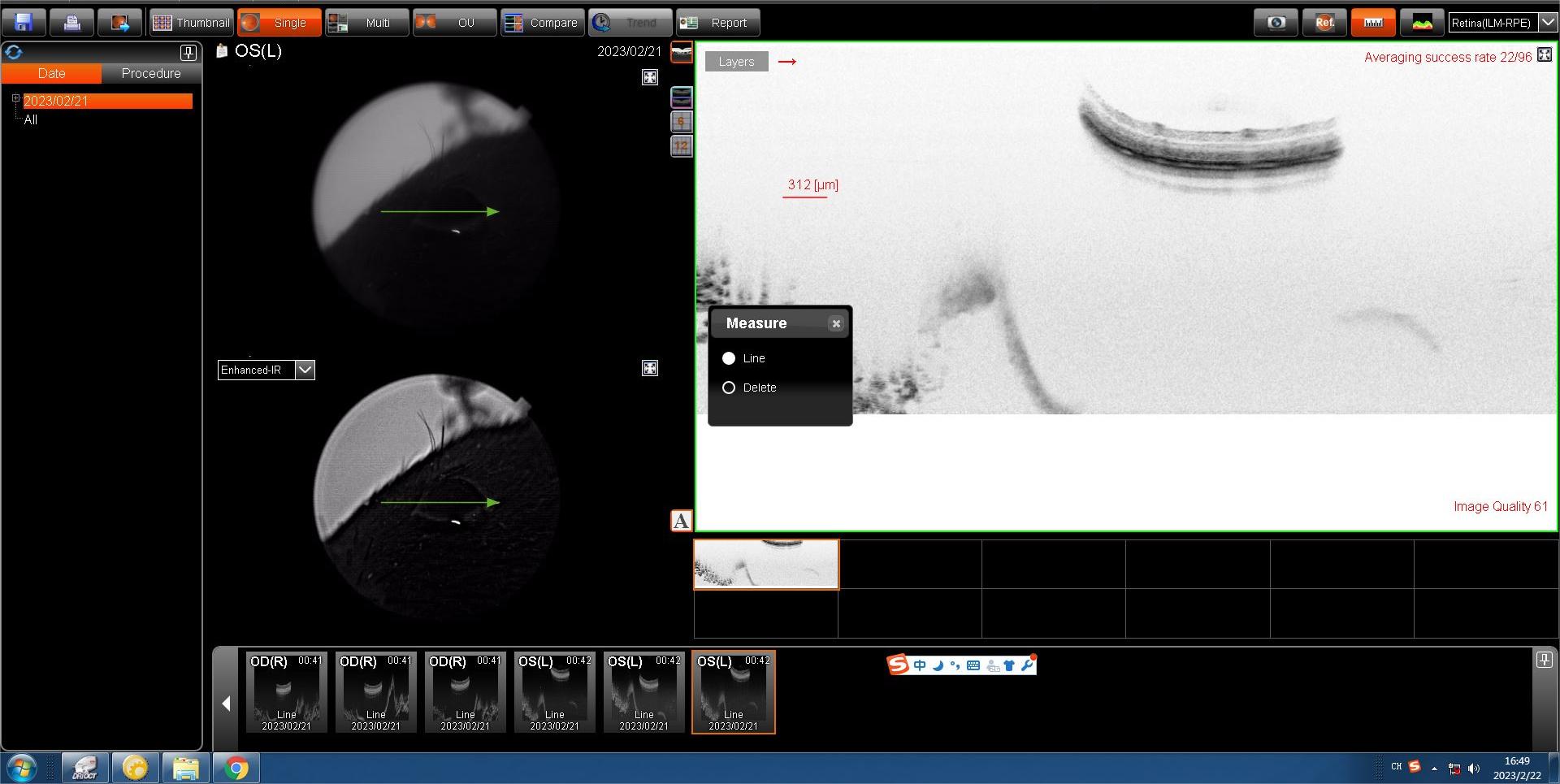

Supplement: Supplementary file 1 [file Data_Sheet_1.ZIP › Original data/Fig 1/OCT images/OCT images/2.RCS/RCS-1.JPG]

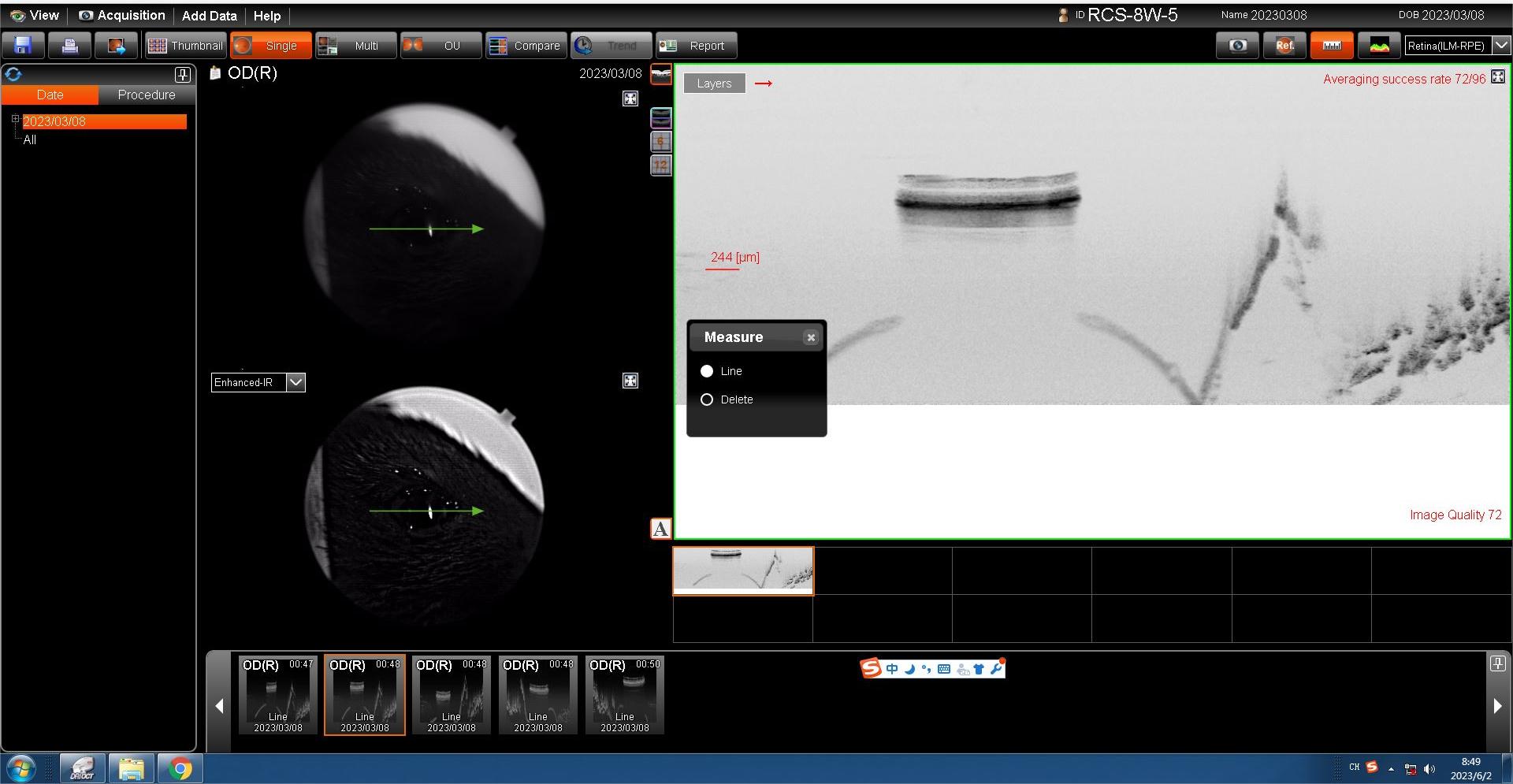

Supplement: Supplementary file 1 [file Data_Sheet_1.ZIP › Original data/Fig 1/OCT images/OCT images/2.RCS/RCS-2.JPG]

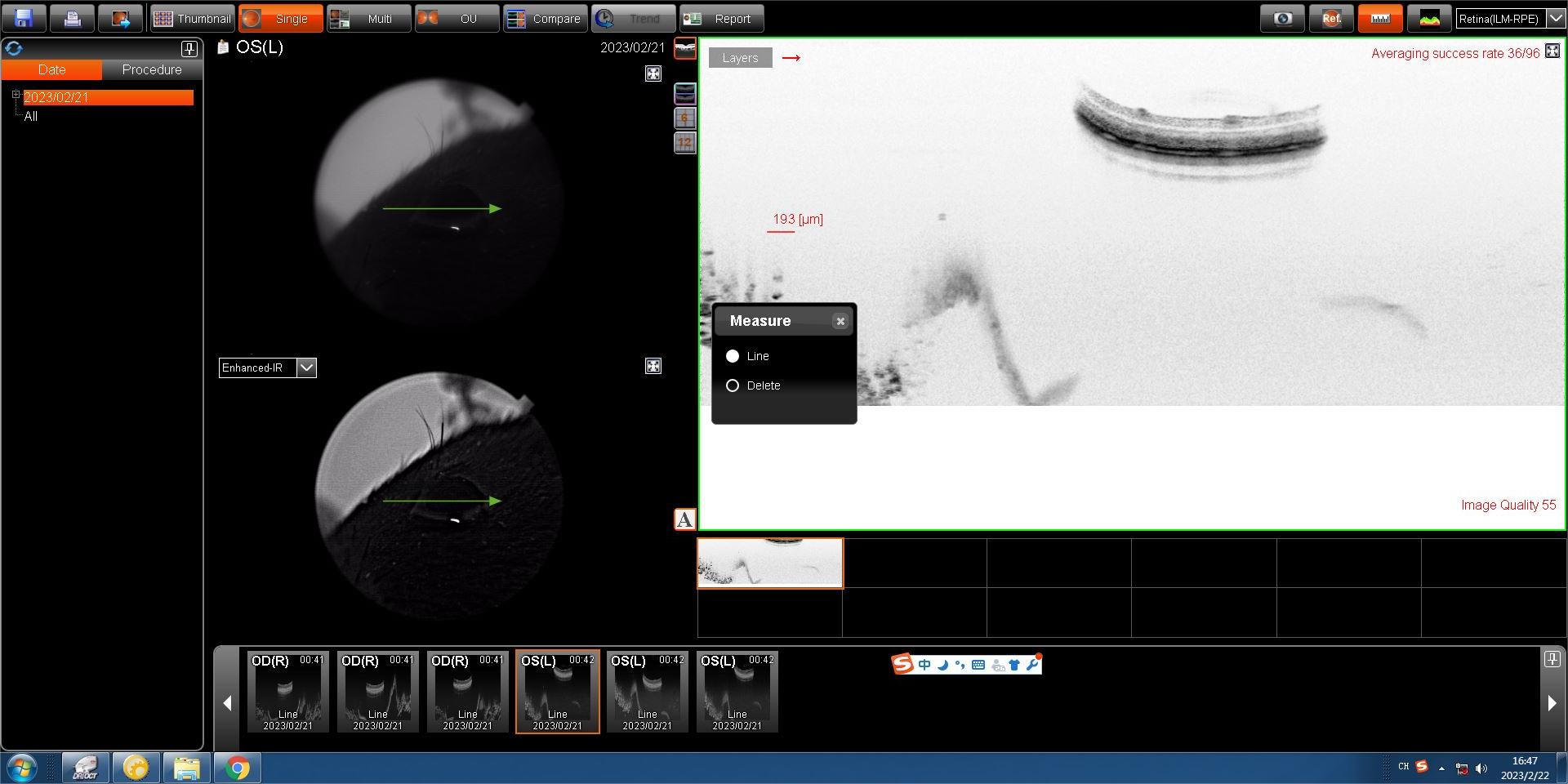

Supplement: Supplementary file 1 [file Data_Sheet_1.ZIP › Original data/Fig 1/OCT images/OCT images/2.RCS/RCS-3.JPG]

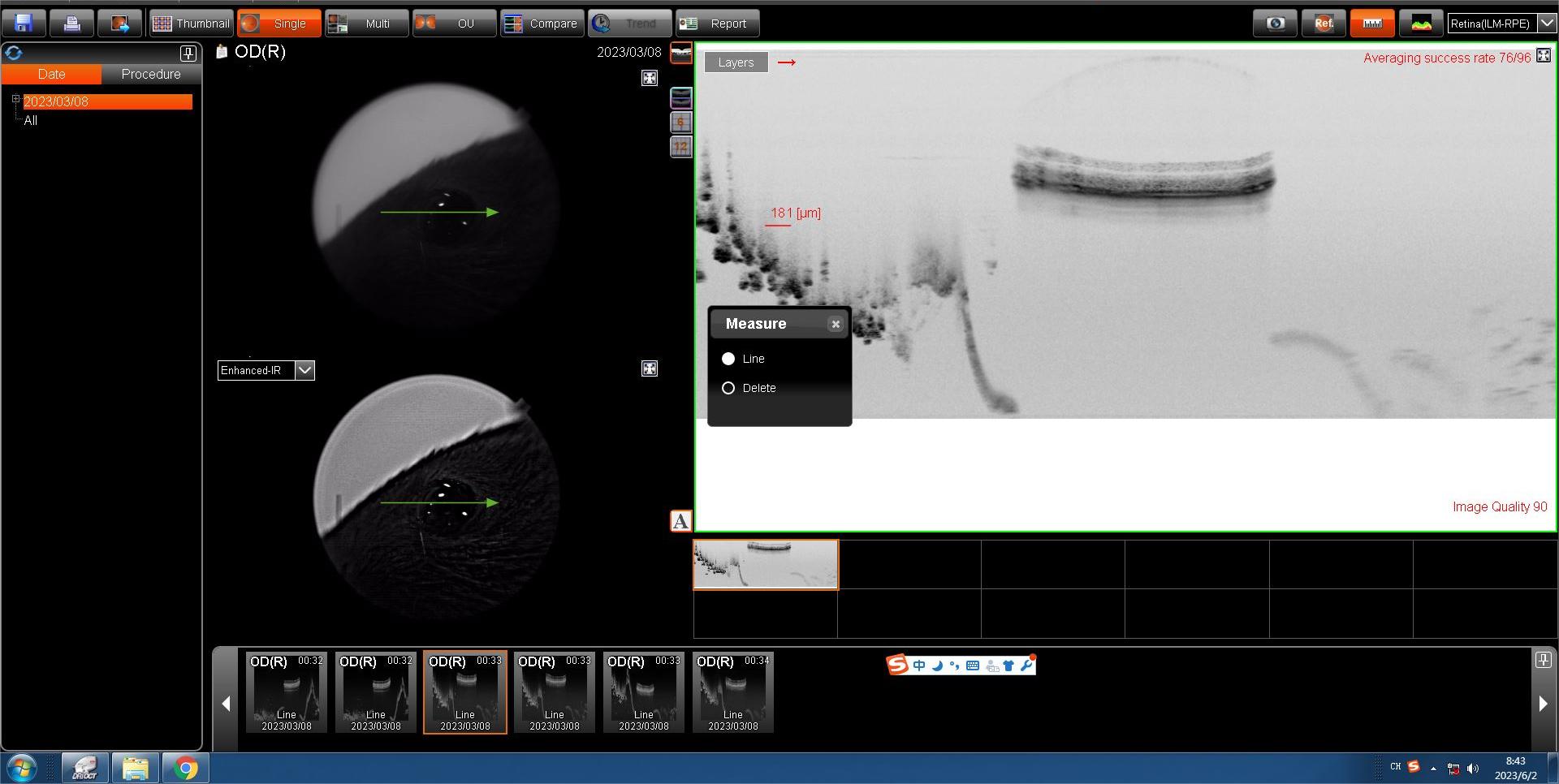

Supplement: Supplementary file 1 [file Data_Sheet_1.ZIP › Original data/Fig 1/OCT images/OCT images/2.RCS/RCS-4.JPG]

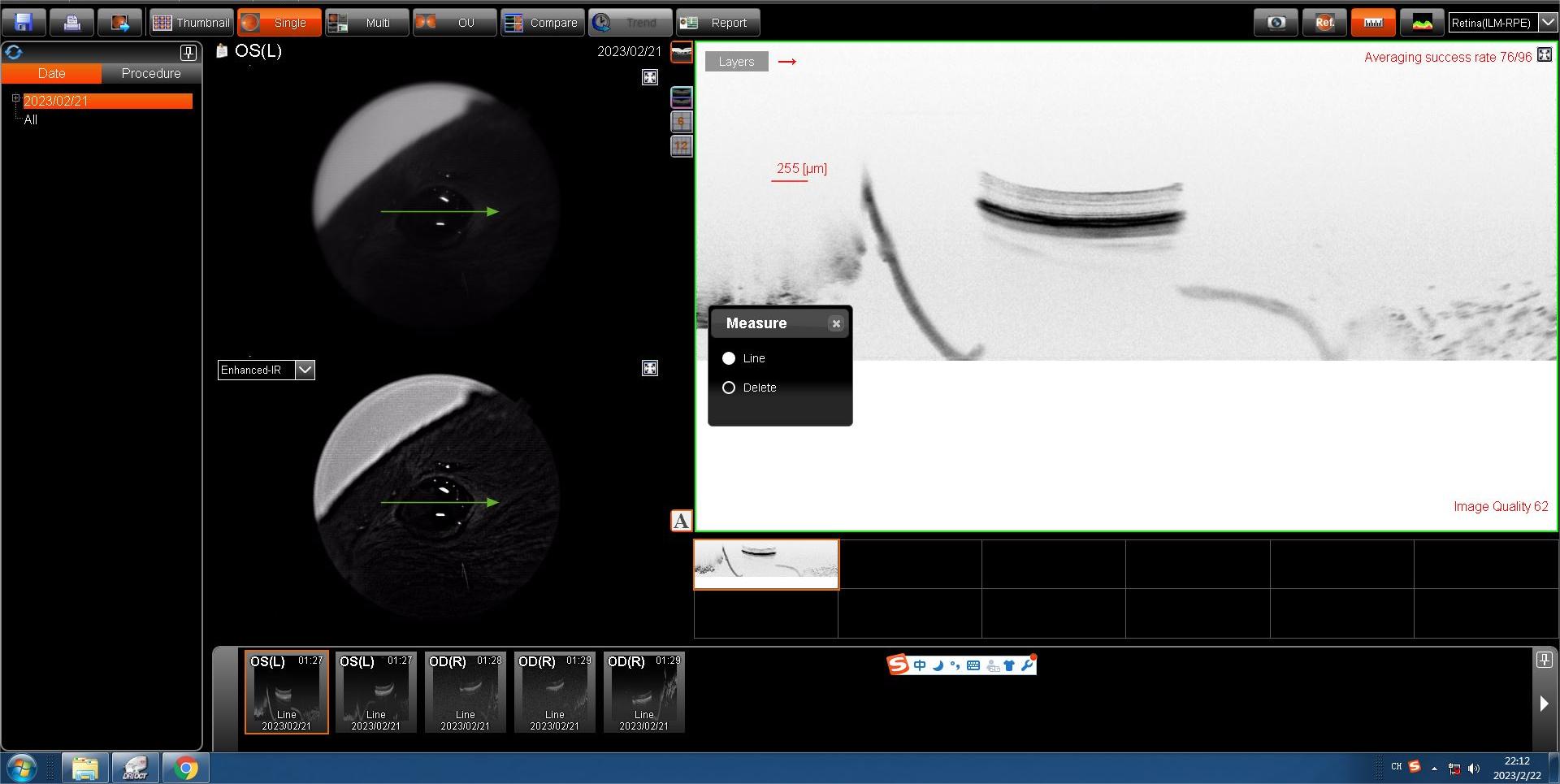

Supplement: Supplementary file 1 [file Data_Sheet_1.ZIP › Original data/Fig 1/OCT images/OCT images/3.RDY/RDY-1.JPG]

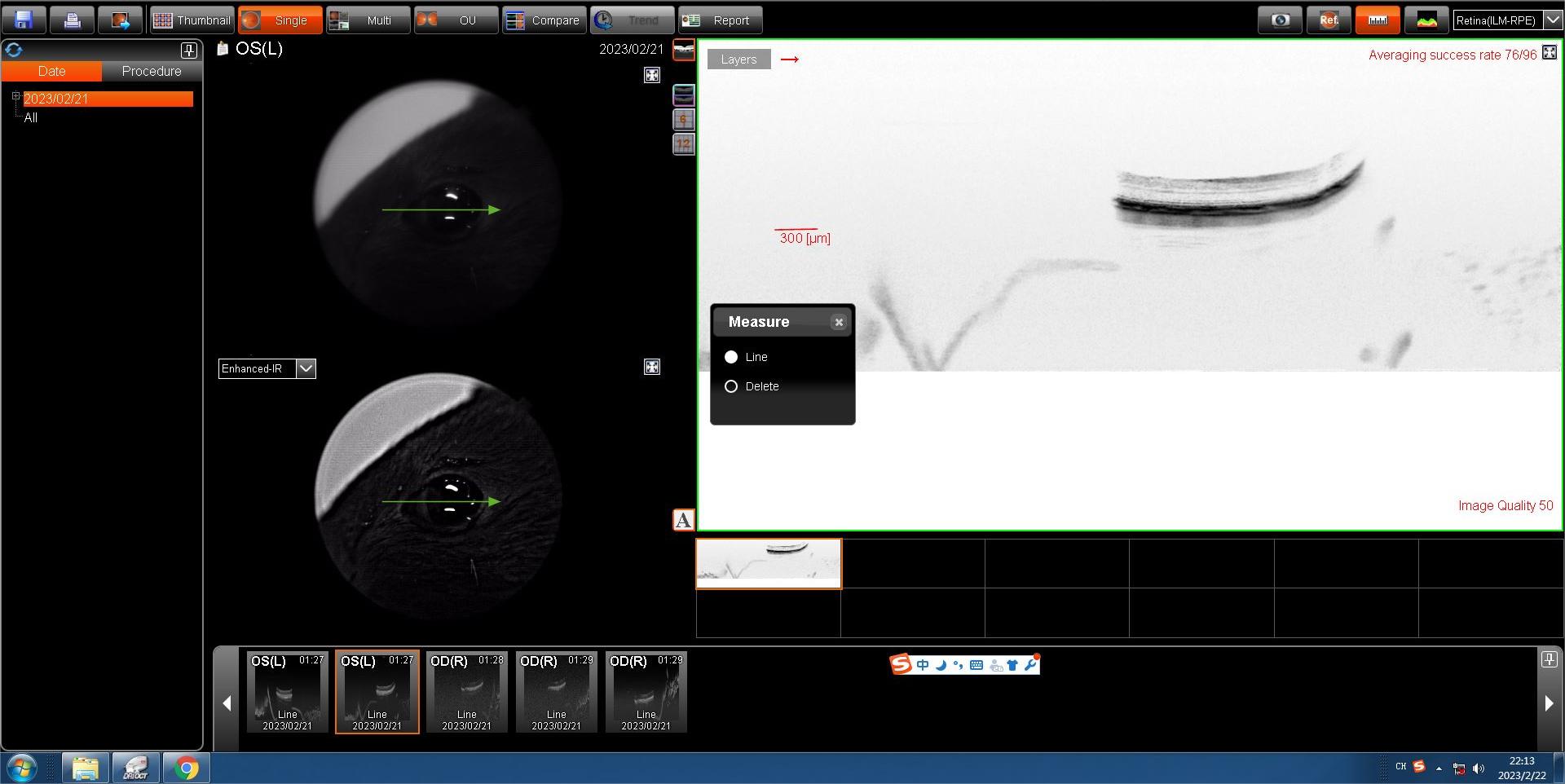

Supplement: Supplementary file 1 [file Data_Sheet_1.ZIP › Original data/Fig 1/OCT images/OCT images/3.RDY/RDY-2.JPG]

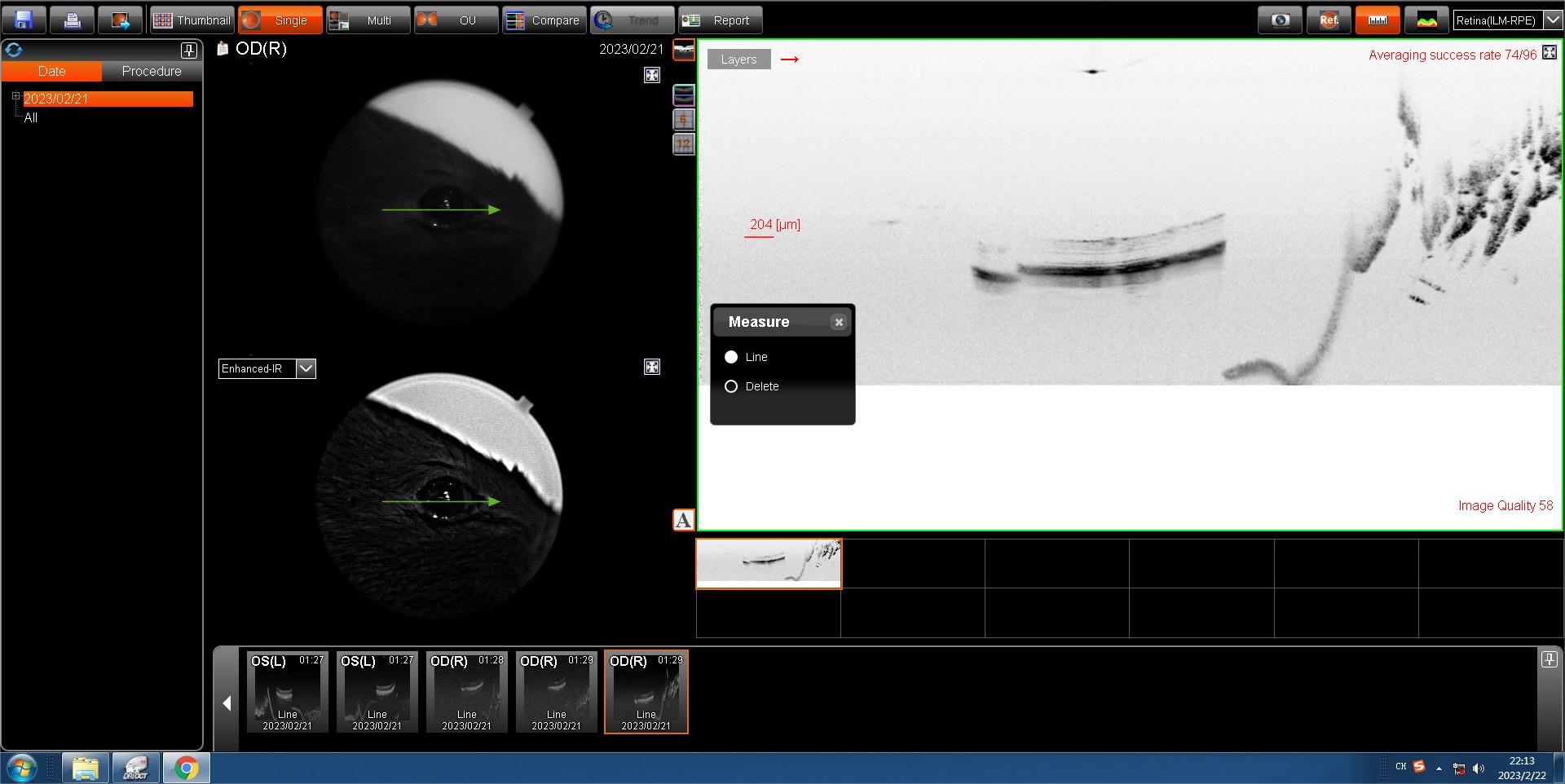

Supplement: Supplementary file 1 [file Data_Sheet_1.ZIP › Original data/Fig 1/OCT images/OCT images/3.RDY/RDY-3.JPG]

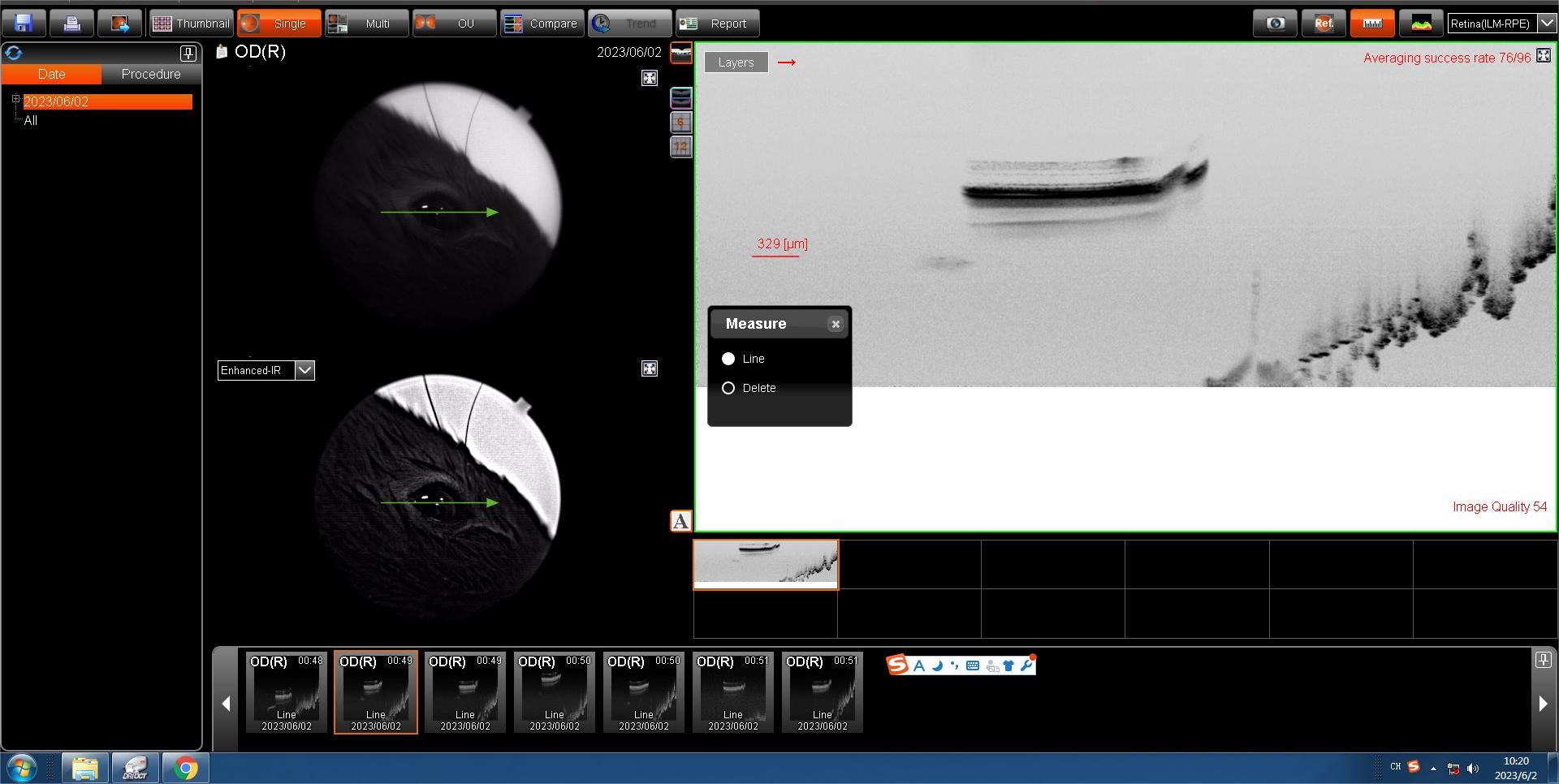

Supplement: Supplementary file 1 [file Data_Sheet_1.ZIP › Original data/Fig 1/OCT images/OCT images/3.RDY/RDY-4.JPG]

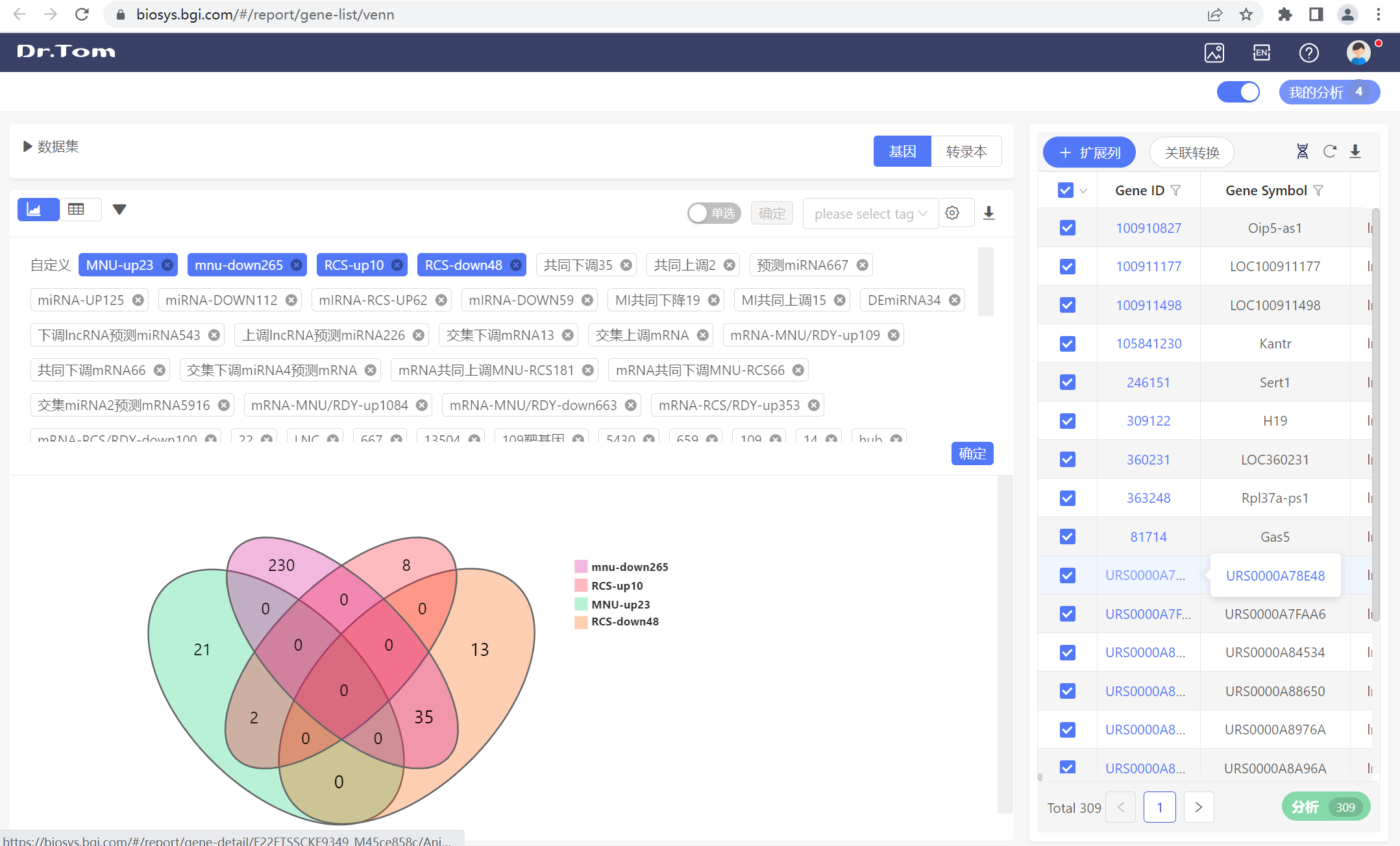

Supplement: Supplementary file 1 [file Data_Sheet_1.ZIP › Original data/Fig 2/Evidence of Volcano plot and Venn plot/Venn-lncRNA.png]

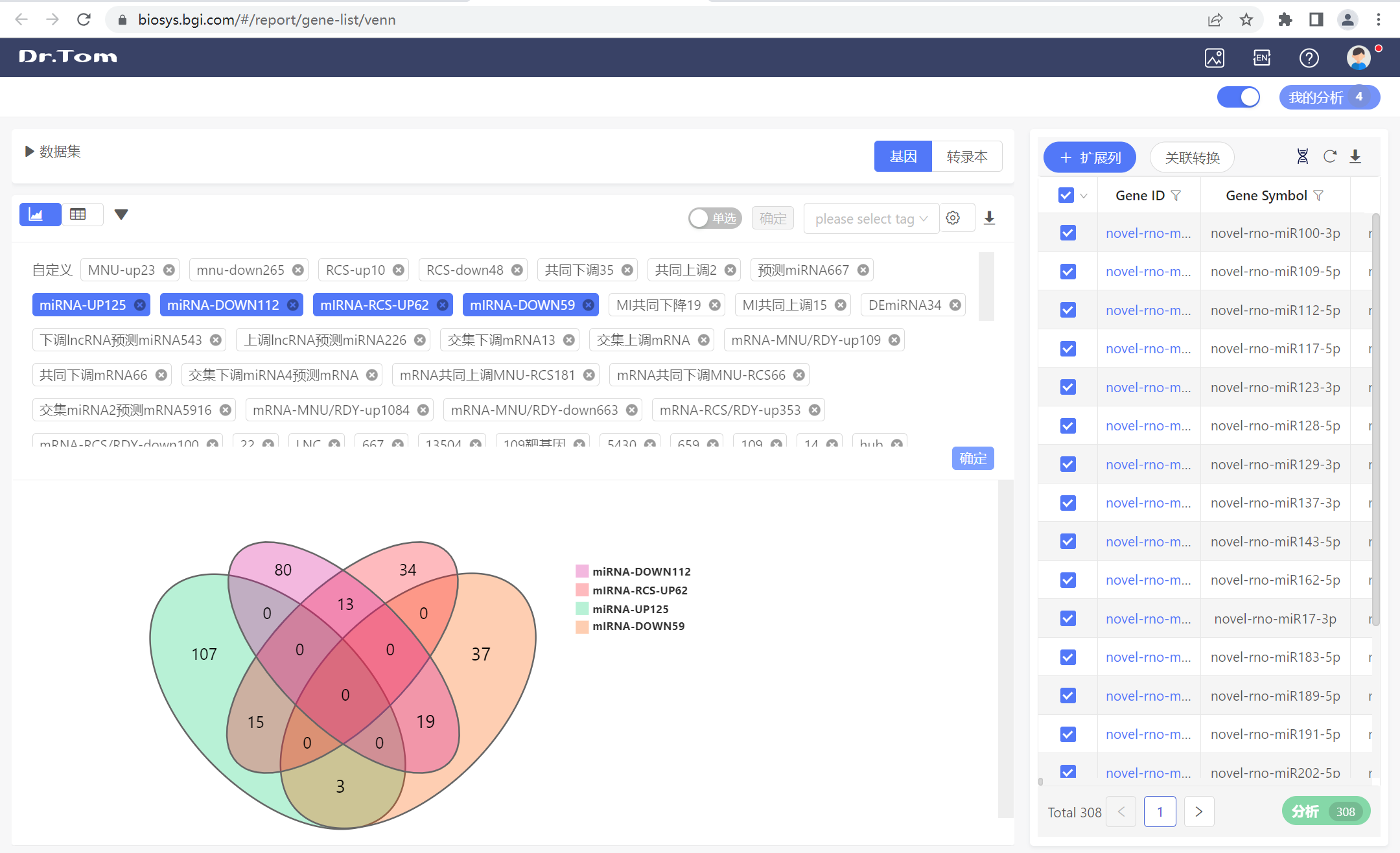

Supplement: Supplementary file 1 [file Data_Sheet_1.ZIP › Original data/Fig 2/Evidence of Volcano plot and Venn plot/Venn-miRNA.png]

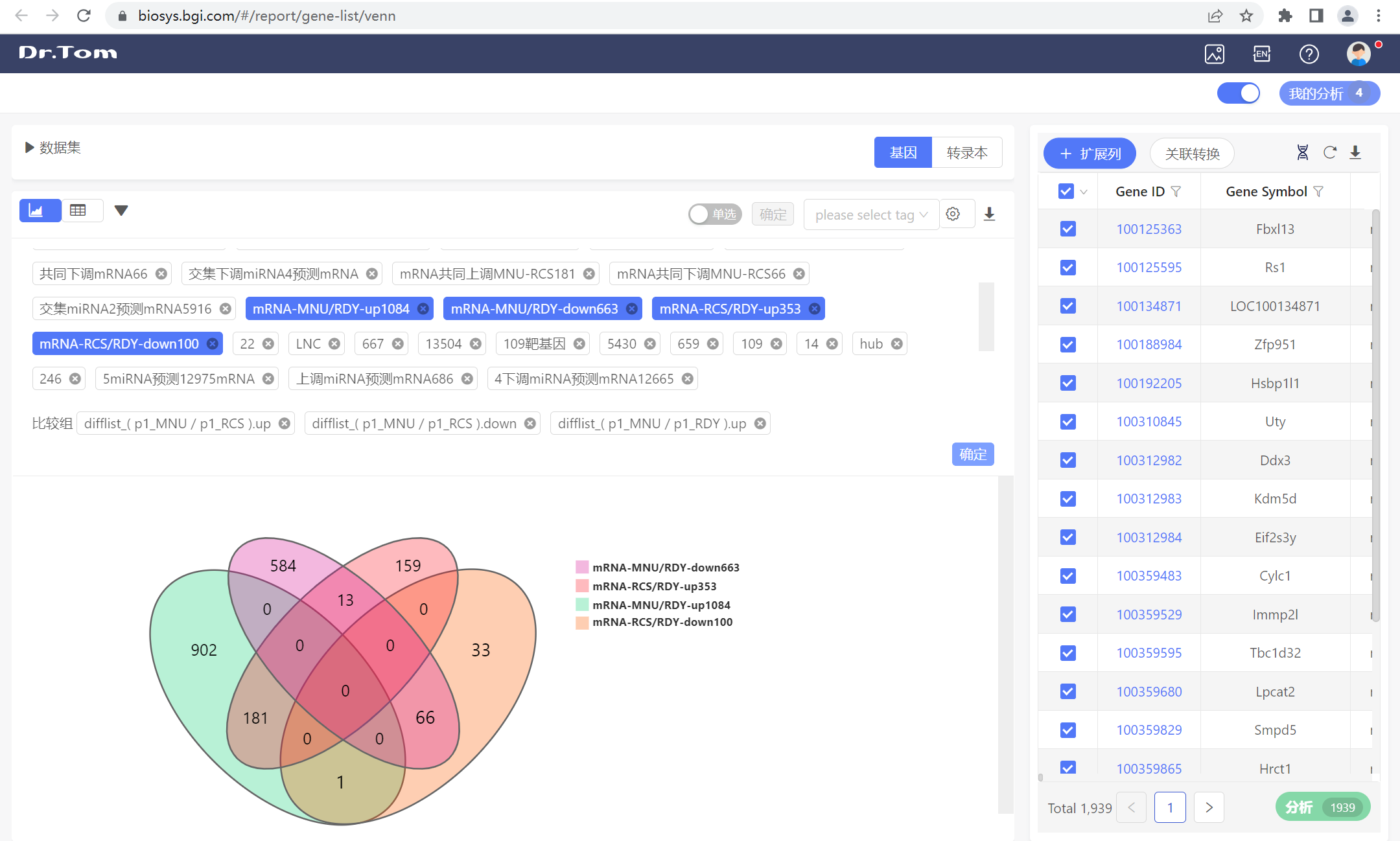

Supplement: Supplementary file 1 [file Data_Sheet_1.ZIP › Original data/Fig 2/Evidence of Volcano plot and Venn plot/Venn-mRNA.png]

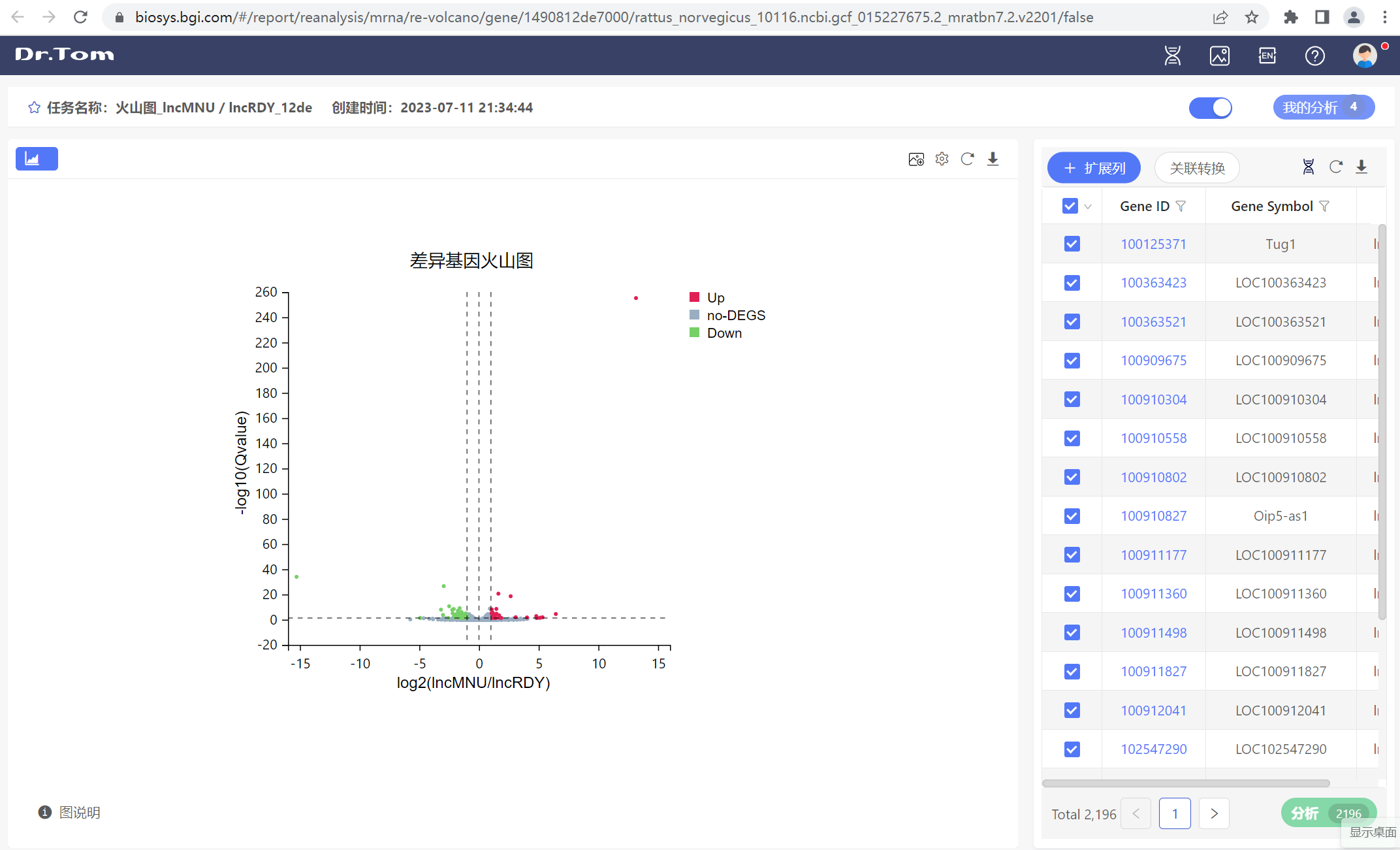

Supplement: Supplementary file 1 [file Data_Sheet_1.ZIP › Original data/Fig 2/Evidence of Volcano plot and Venn plot/Volcano plot-lncRNA in MNU vs RDY group.png]

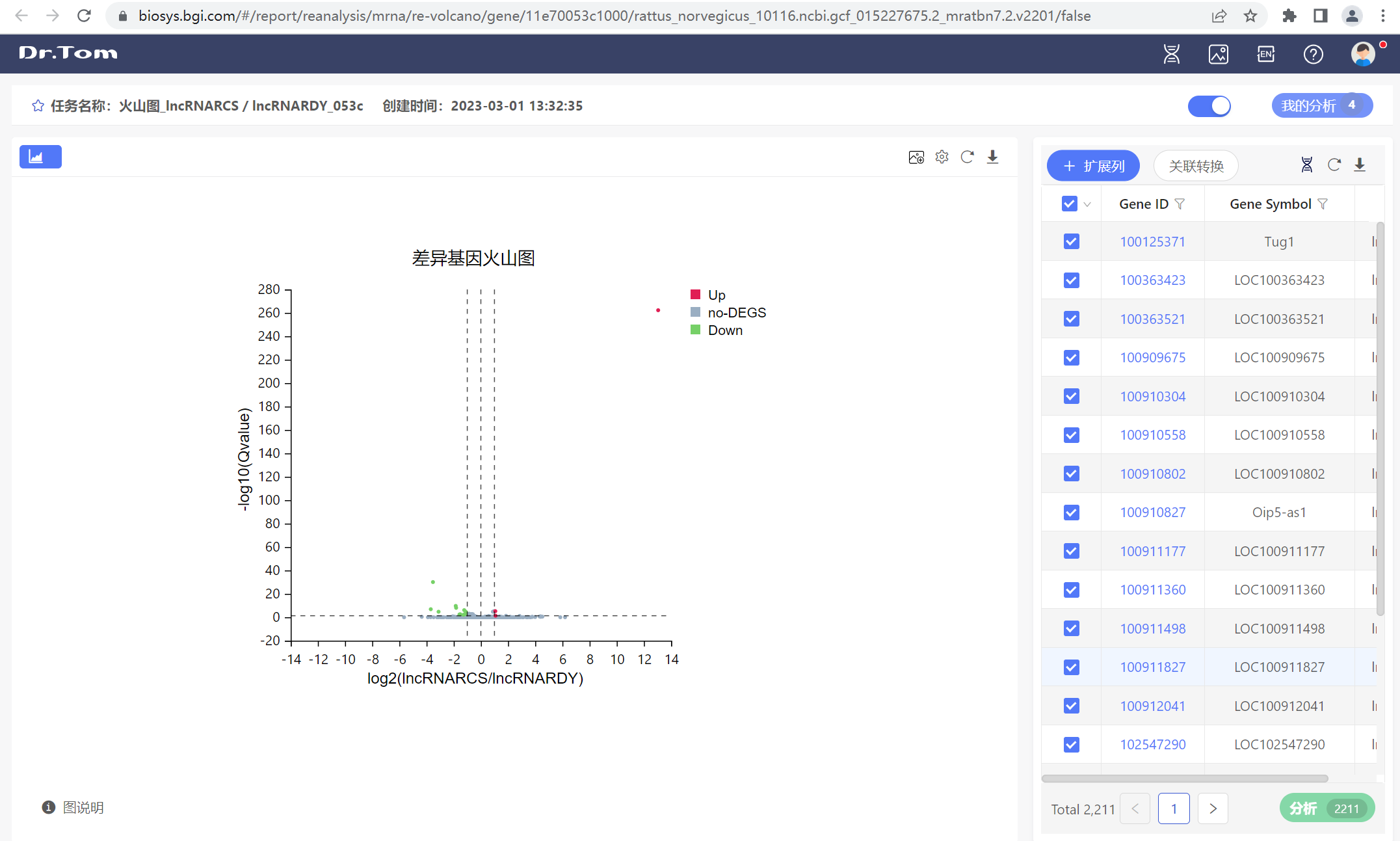

Supplement: Supplementary file 1 [file Data_Sheet_1.ZIP › Original data/Fig 2/Evidence of Volcano plot and Venn plot/Volcano plot-lncRNA in RCS vs RDY group.png]

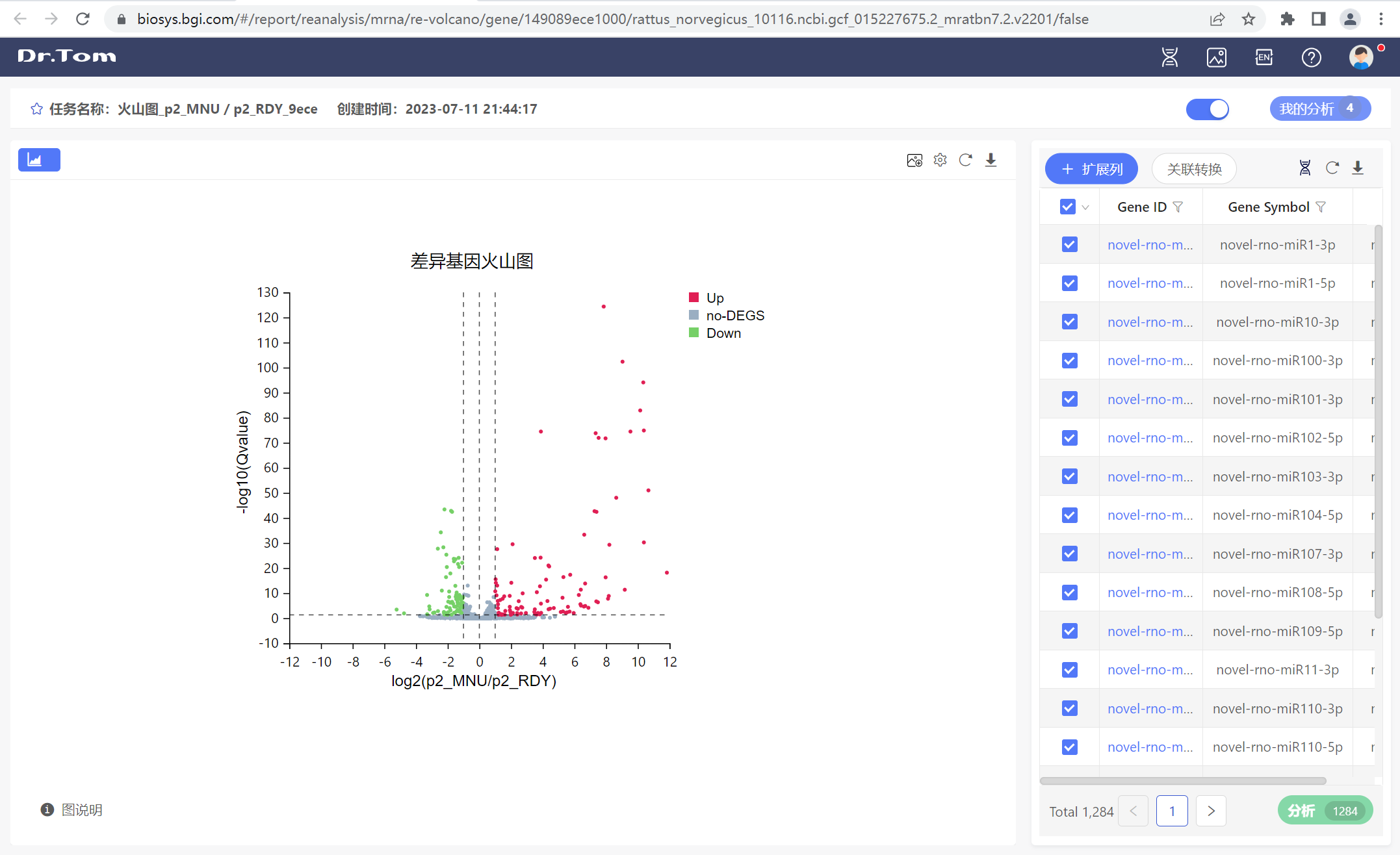

Supplement: Supplementary file 1 [file Data_Sheet_1.ZIP › Original data/Fig 2/Evidence of Volcano plot and Venn plot/Volcano plot-miRNA in MNU vs RDY group.png]

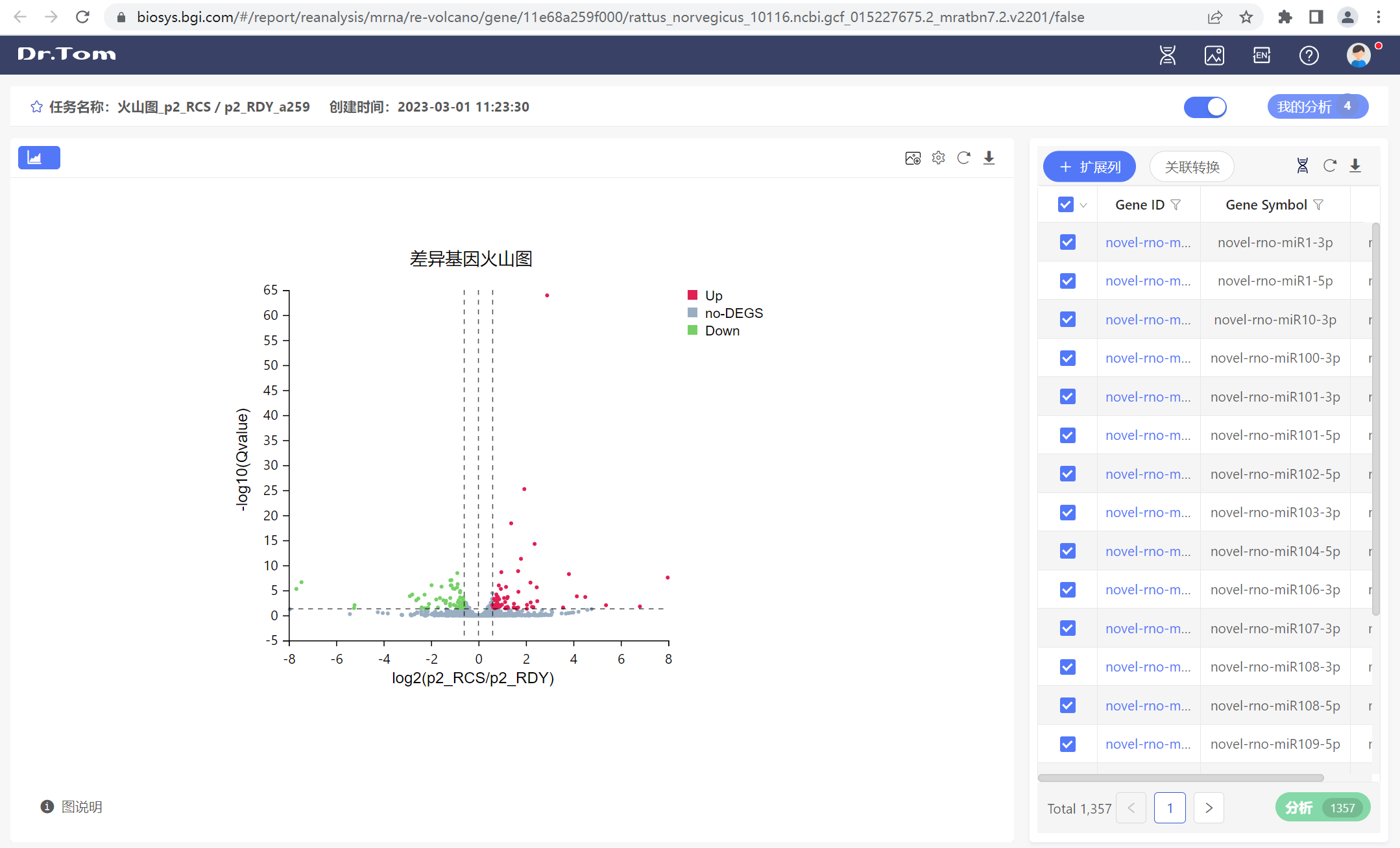

Supplement: Supplementary file 1 [file Data_Sheet_1.ZIP › Original data/Fig 2/Evidence of Volcano plot and Venn plot/Volcano plot-miRNA in RCS vs RDY group.png]

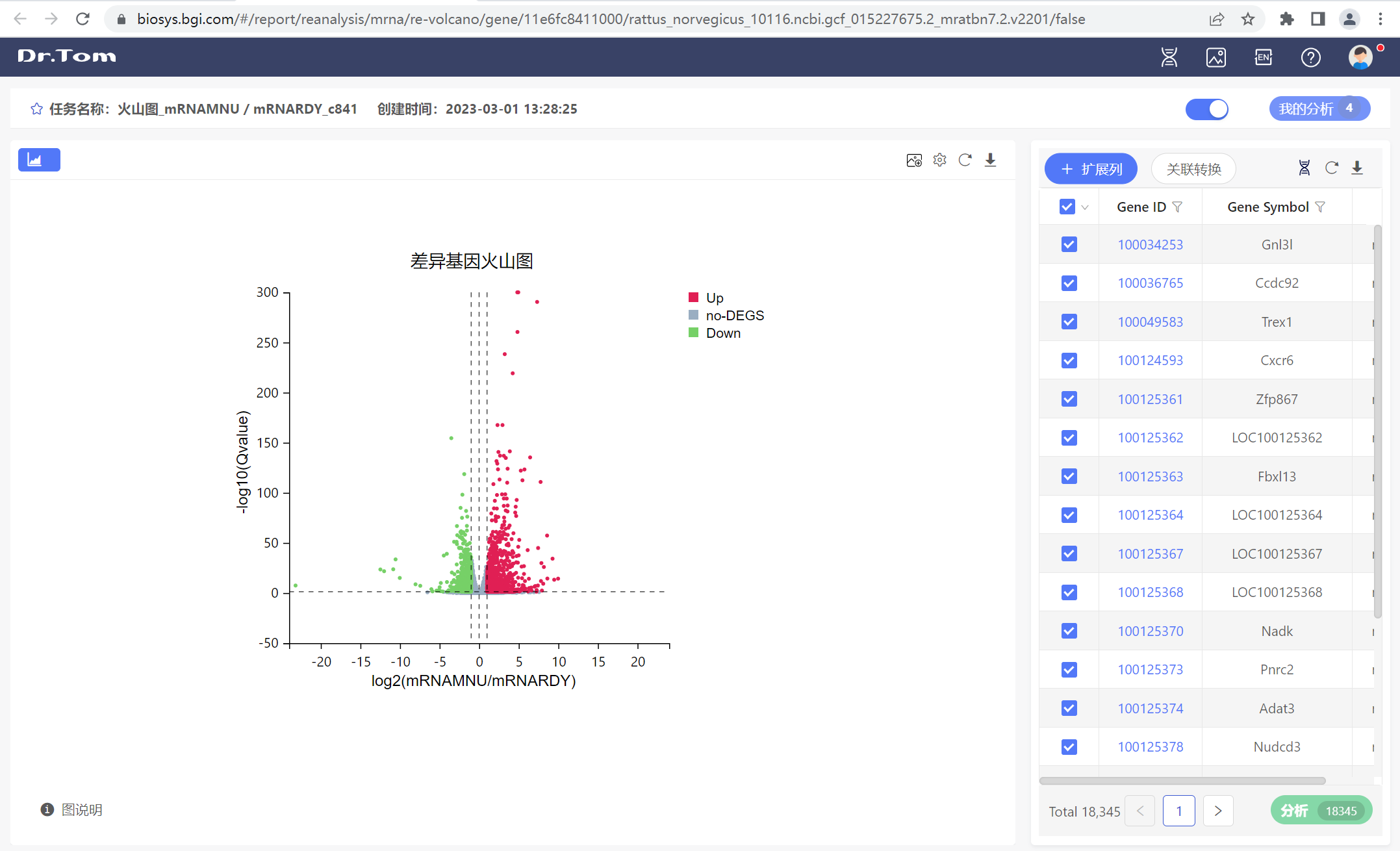

Supplement: Supplementary file 1 [file Data_Sheet_1.ZIP › Original data/Fig 2/Evidence of Volcano plot and Venn plot/Volcano plot-mRNA in MNU vs RDY group.png]

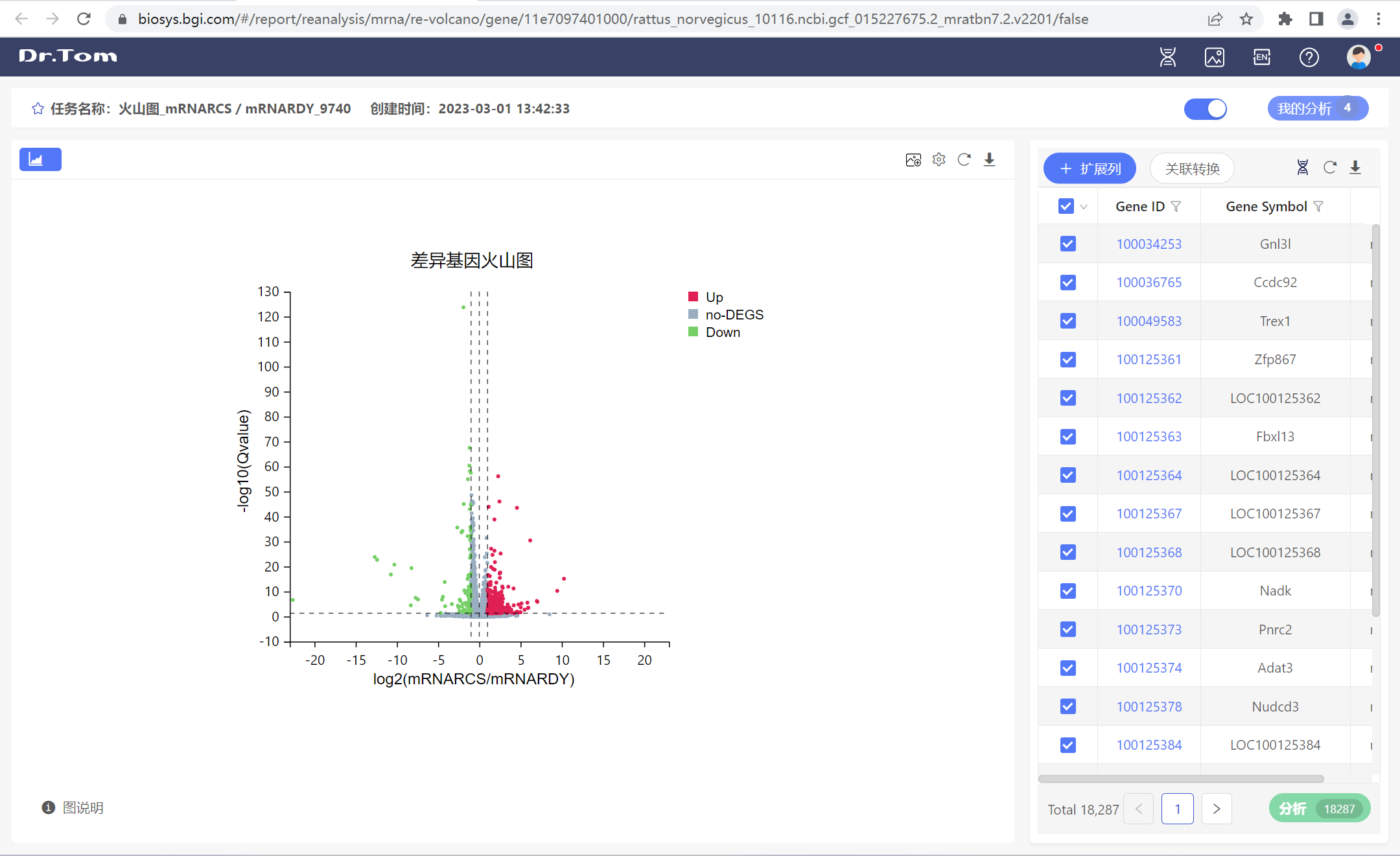

Supplement: Supplementary file 1 [file Data_Sheet_1.ZIP › Original data/Fig 2/Evidence of Volcano plot and Venn plot/Volcano plot-mRNA in RCS vs RDY group.png]

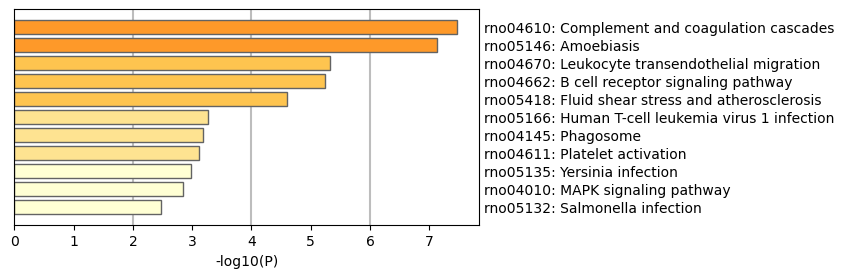

Supplement: Supplementary file 1 [file Data_Sheet_1.ZIP › Original data/Fig 6/Heatmap Selected GO.png]

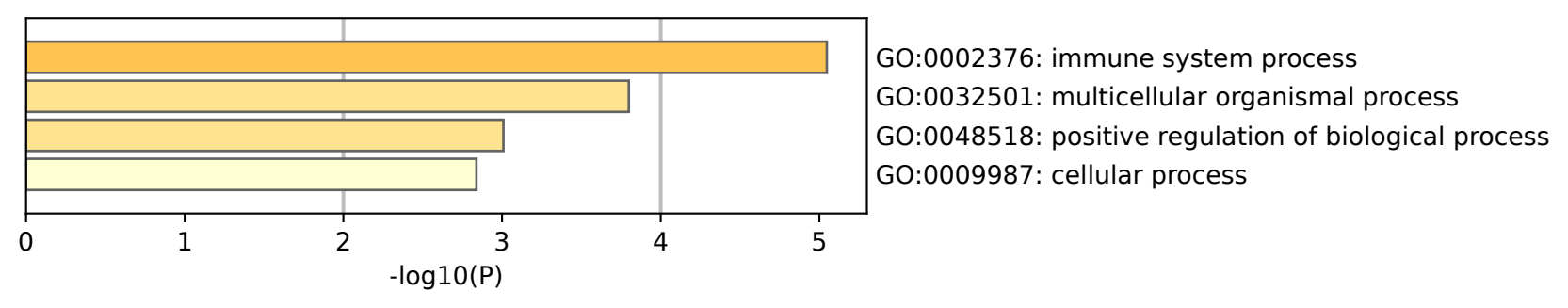

Supplement: Supplementary file 1 [file Data_Sheet_1.ZIP › Original data/Fig 8/Result 1. Heatmap Selected GO Parent.pdf]

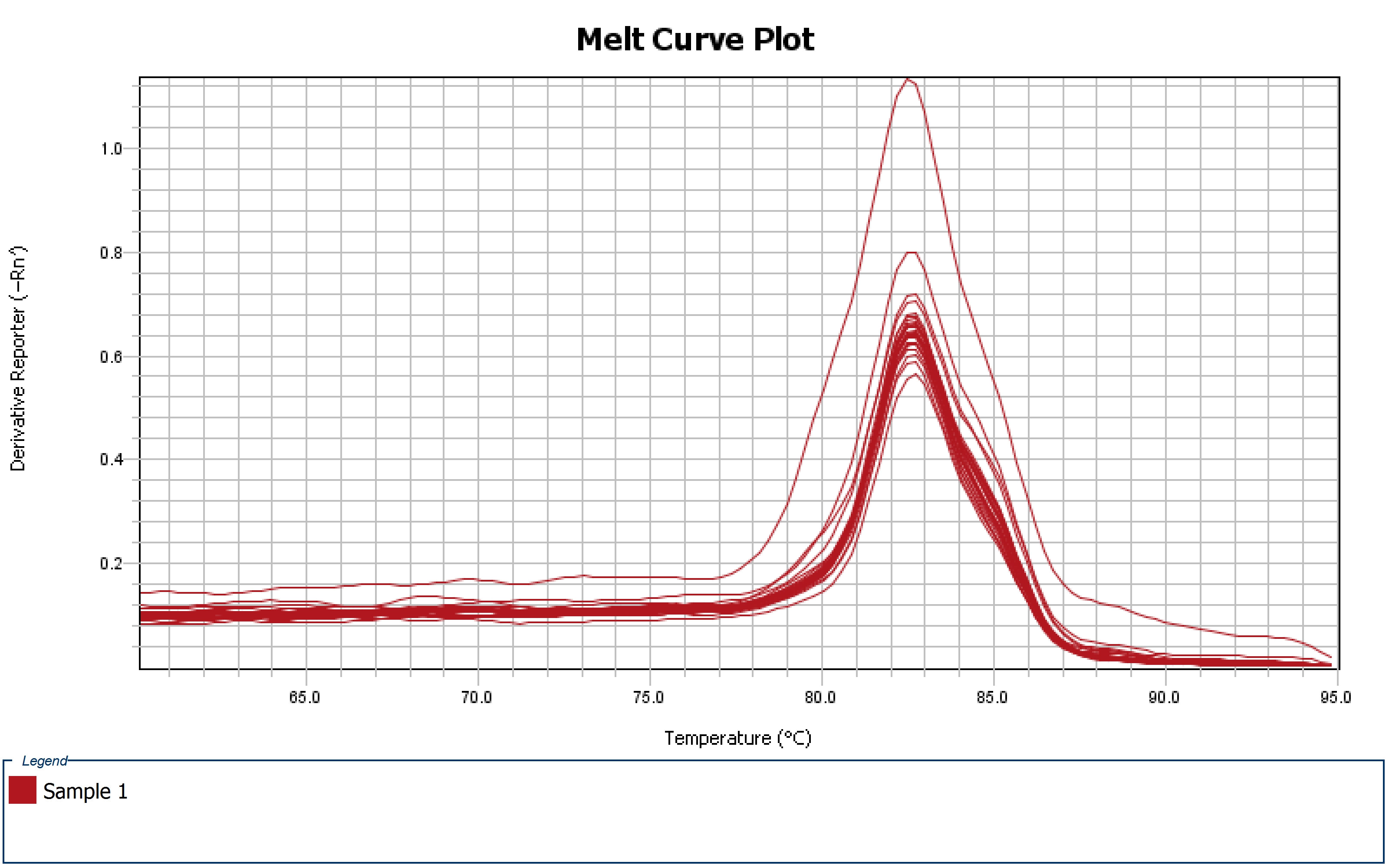

Supplement: Supplementary file 1 [file Data_Sheet_1.ZIP › Original data/Fig 9/File 1. Solubilization and amplification curves of CeRNAs/C1qa M.jpg]

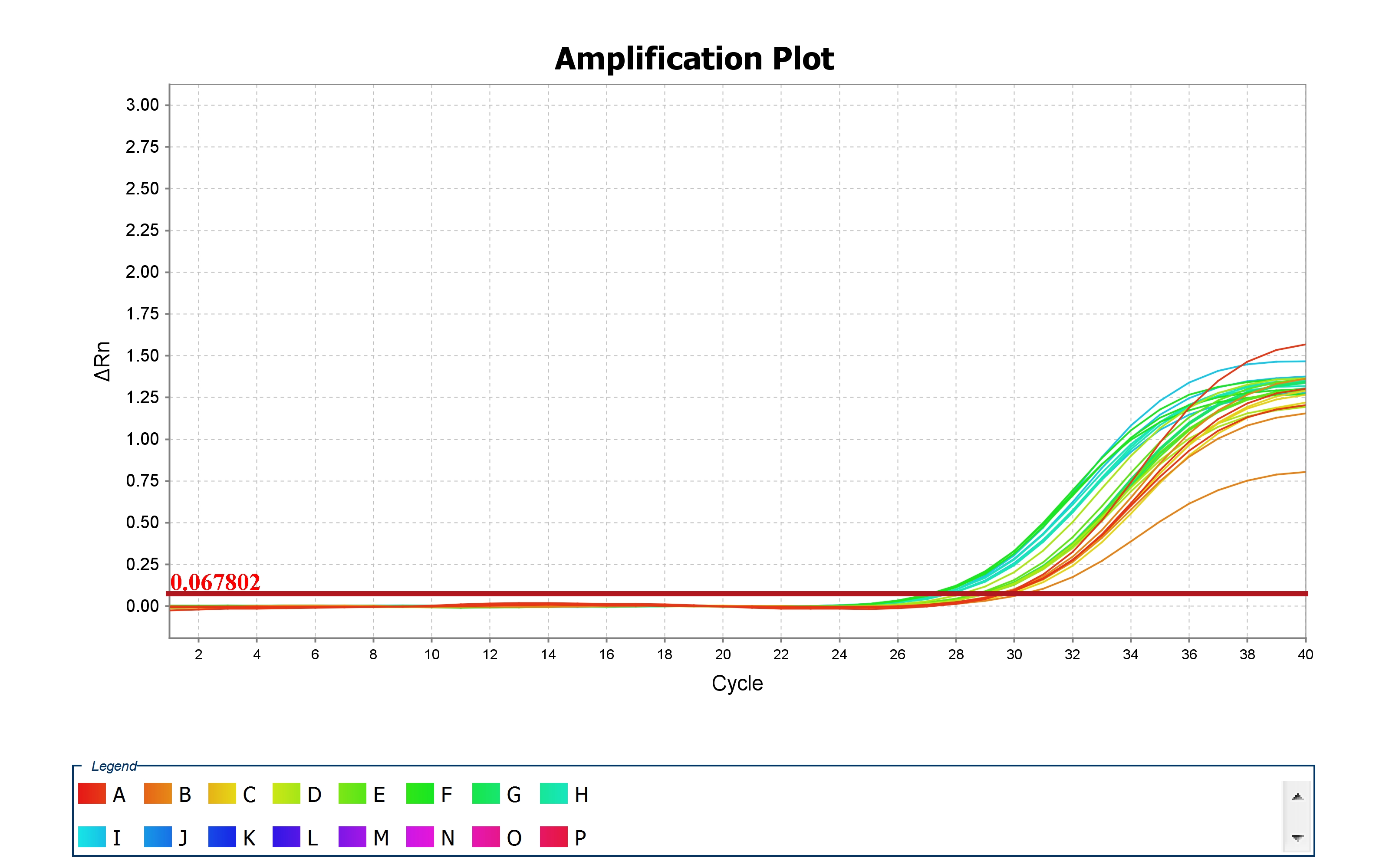

Supplement: Supplementary file 1 [file Data_Sheet_1.ZIP › Original data/Fig 9/File 1. Solubilization and amplification curves of CeRNAs/C1qa.jpg]

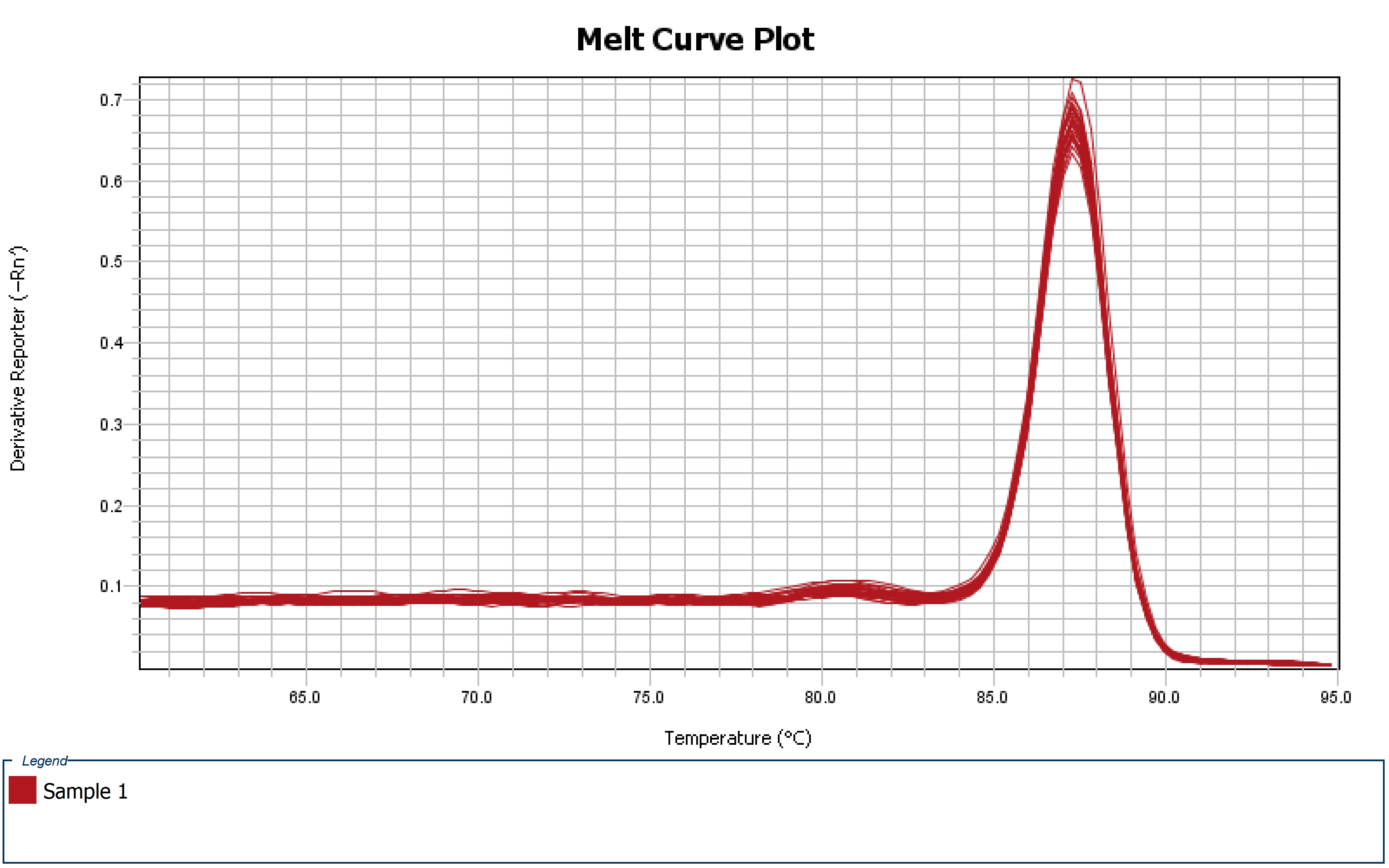

Supplement: Supplementary file 1 [file Data_Sheet_1.ZIP › Original data/Fig 9/File 1. Solubilization and amplification curves of CeRNAs/C1qb M.jpg]

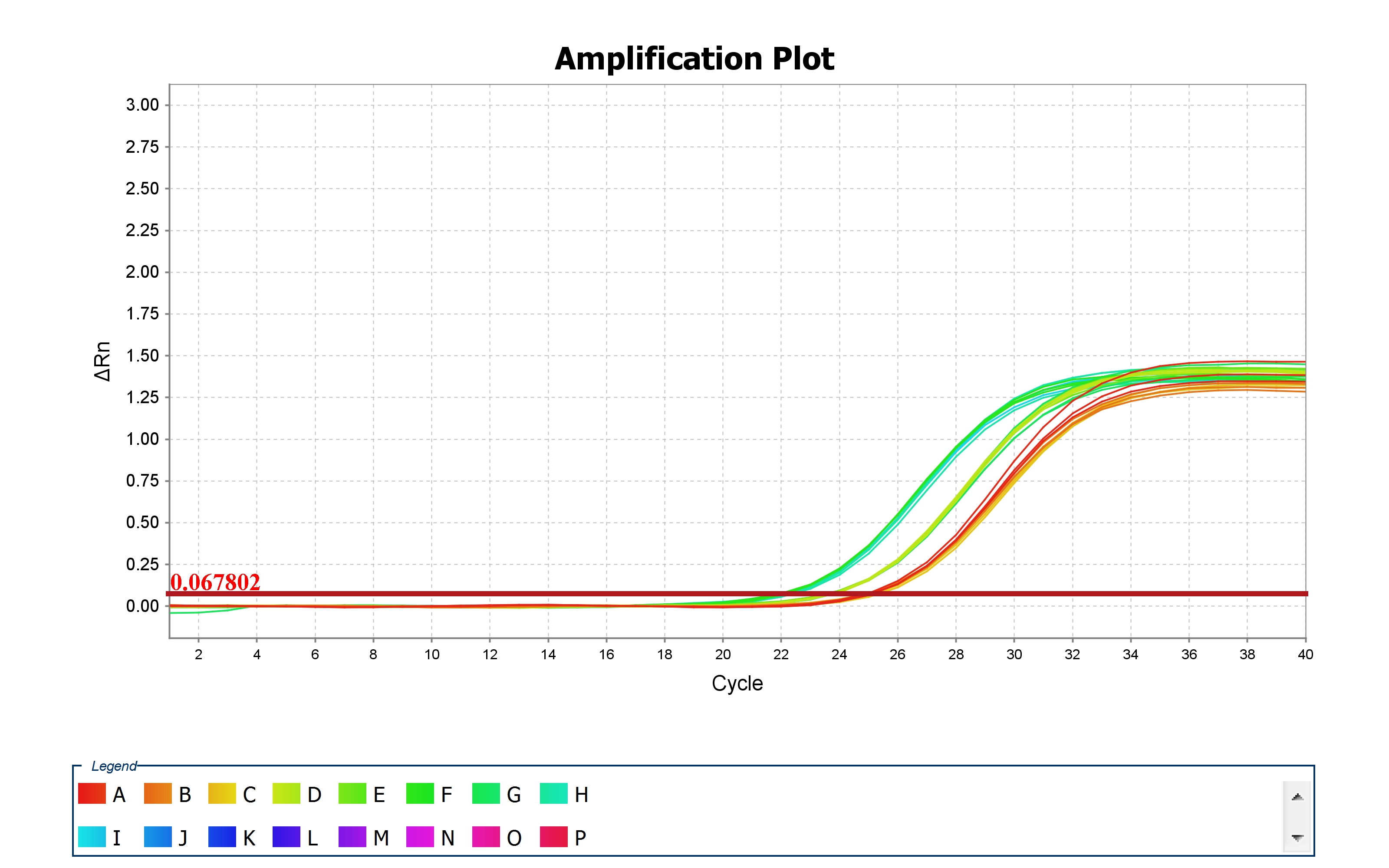

Supplement: Supplementary file 1 [file Data_Sheet_1.ZIP › Original data/Fig 9/File 1. Solubilization and amplification curves of CeRNAs/C1qb.jpg]

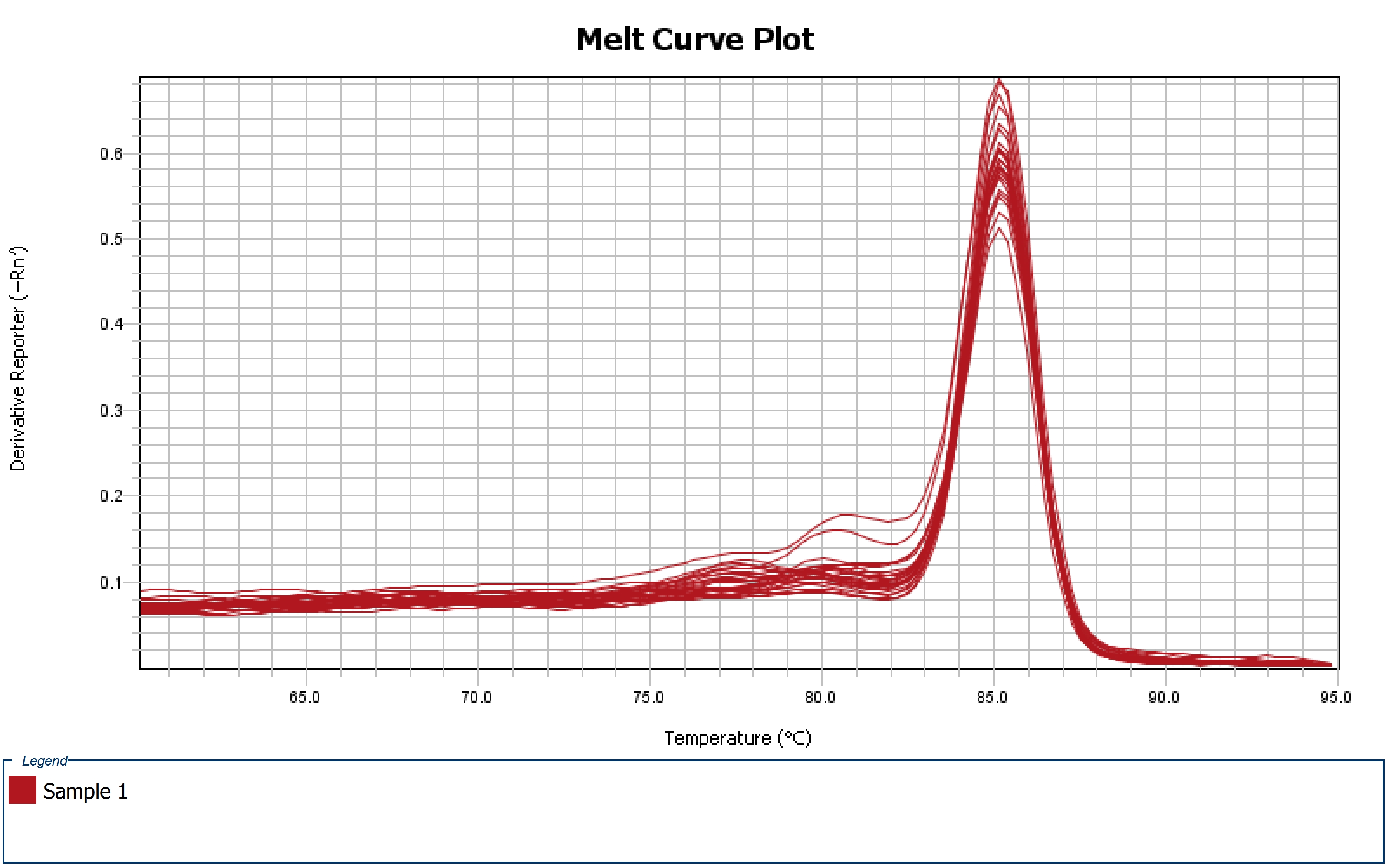

Supplement: Supplementary file 1 [file Data_Sheet_1.ZIP › Original data/Fig 9/File 1. Solubilization and amplification curves of CeRNAs/Card11 M.jpg]

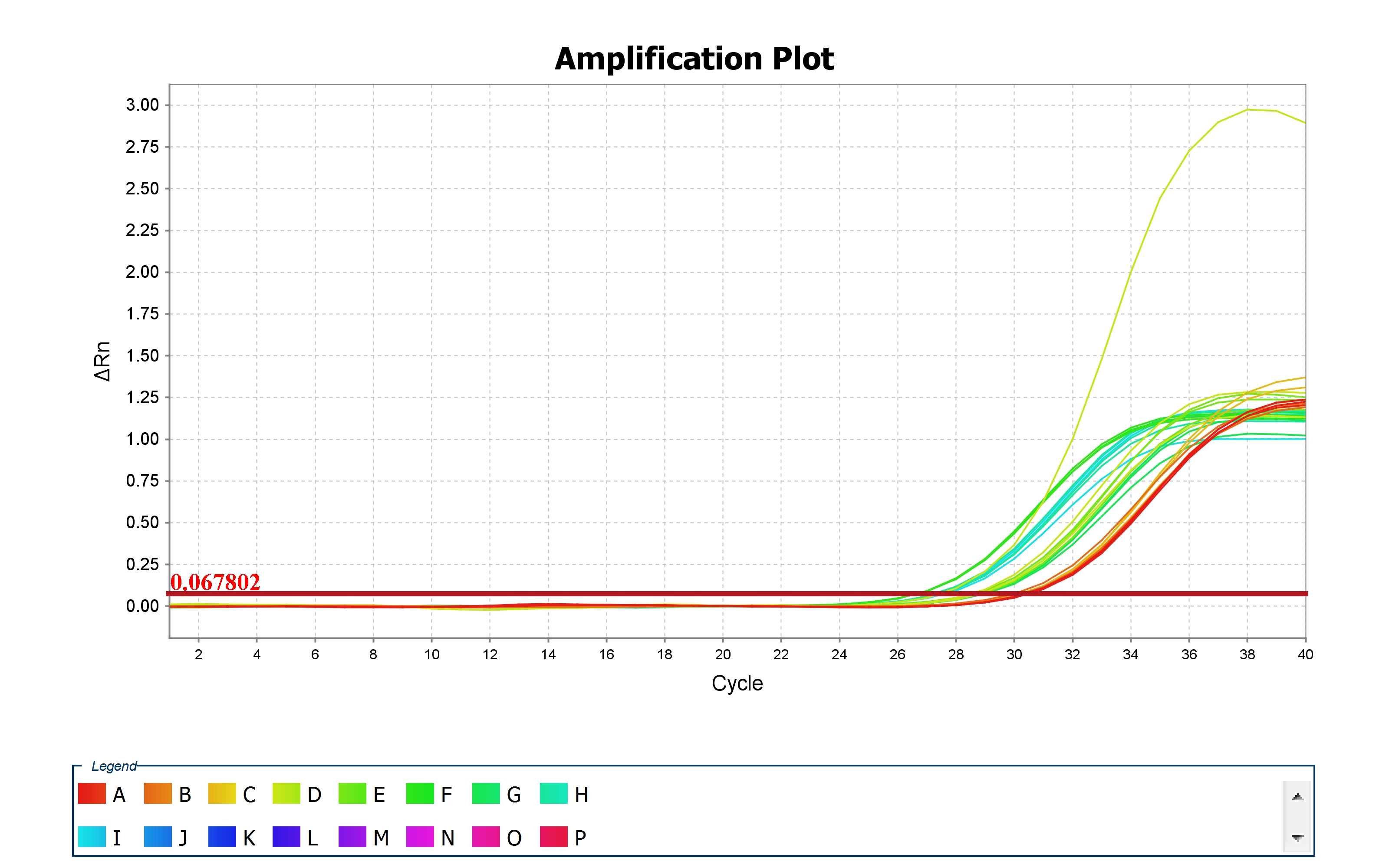

Supplement: Supplementary file 1 [file Data_Sheet_1.ZIP › Original data/Fig 9/File 1. Solubilization and amplification curves of CeRNAs/Card11.jpg]

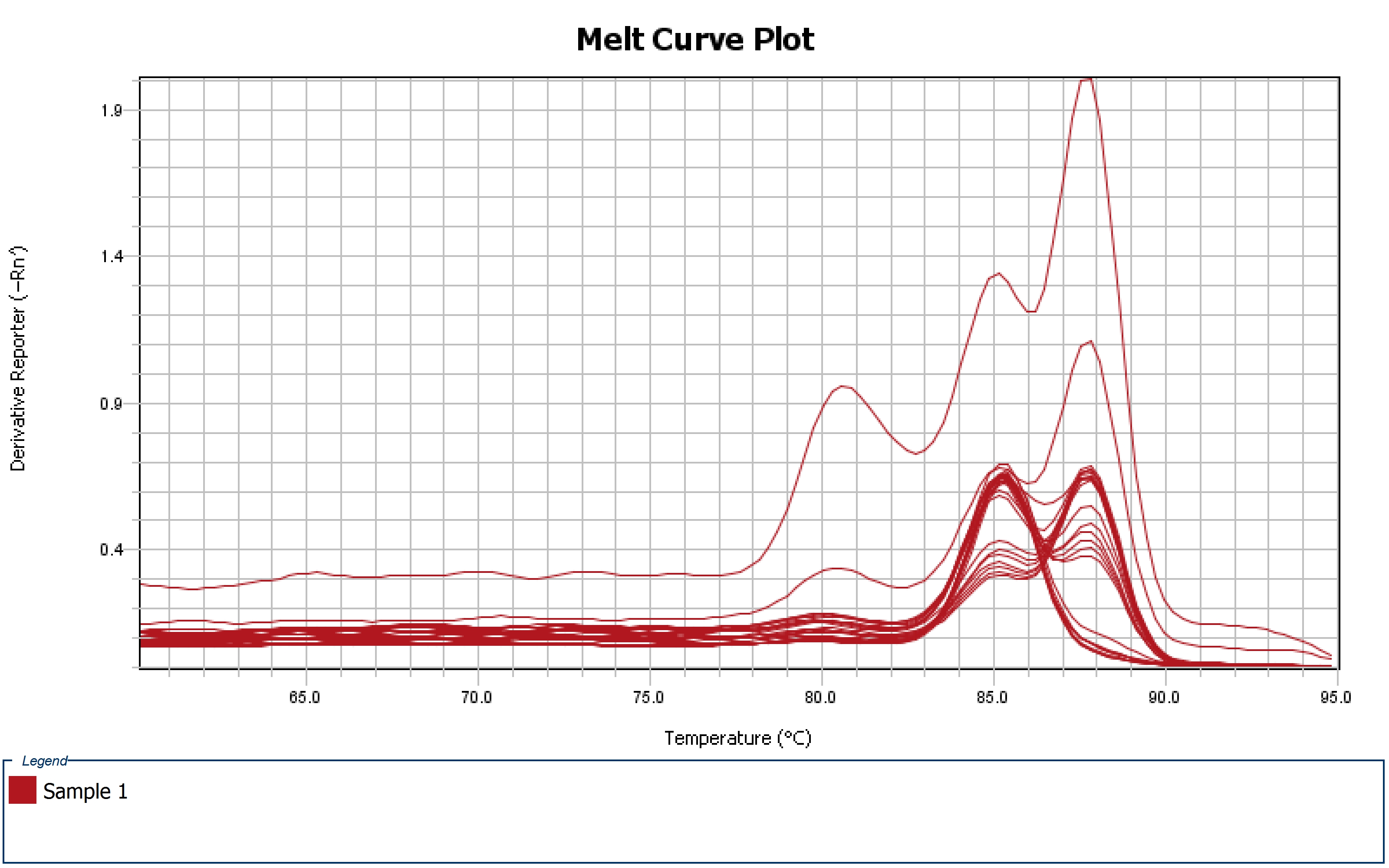

Supplement: Supplementary file 1 [file Data_Sheet_1.ZIP › Original data/Fig 9/File 1. Solubilization and amplification curves of CeRNAs/Csf2rb M.jpg]

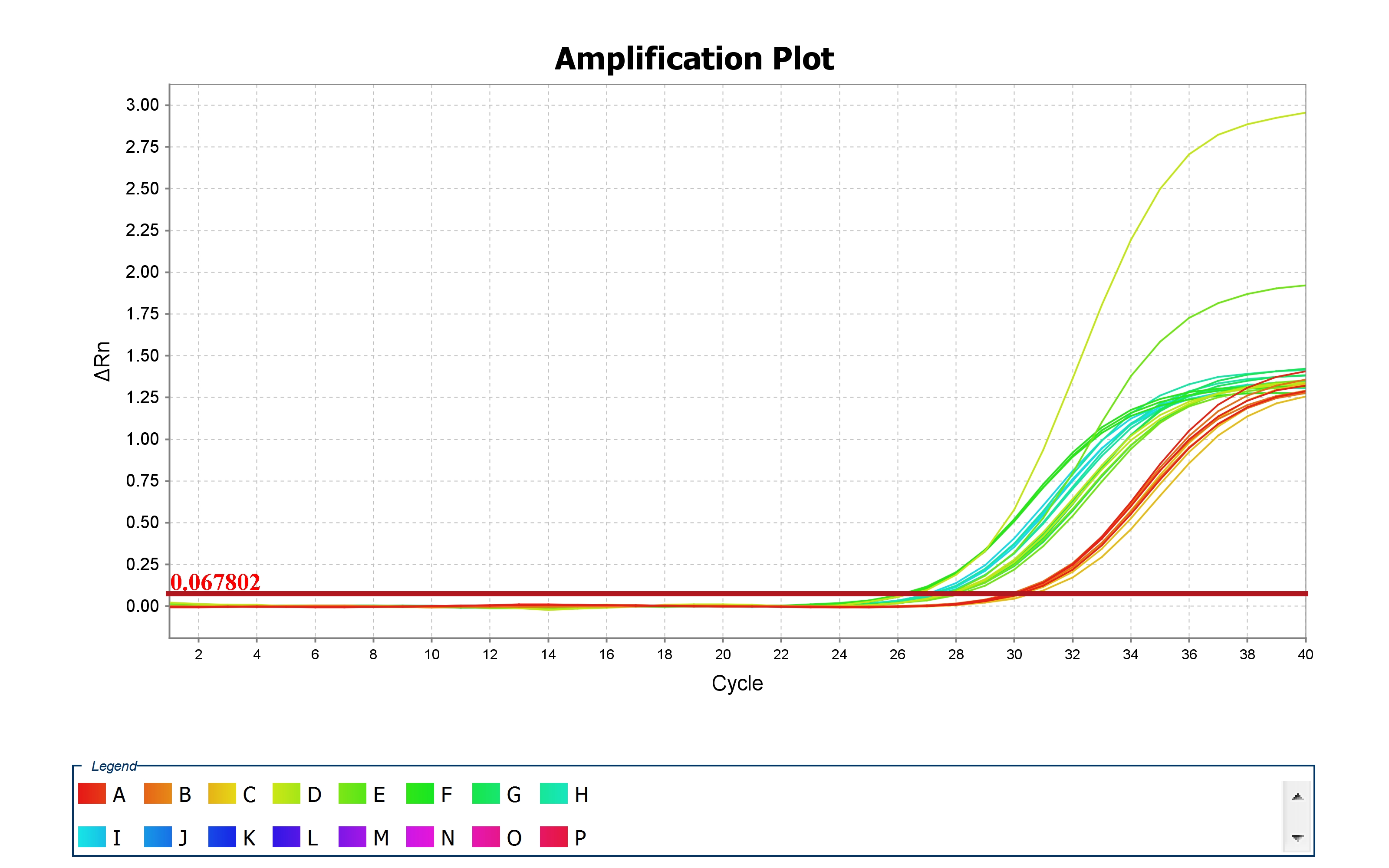

Supplement: Supplementary file 1 [file Data_Sheet_1.ZIP › Original data/Fig 9/File 1. Solubilization and amplification curves of CeRNAs/Csf2rb.jpg]

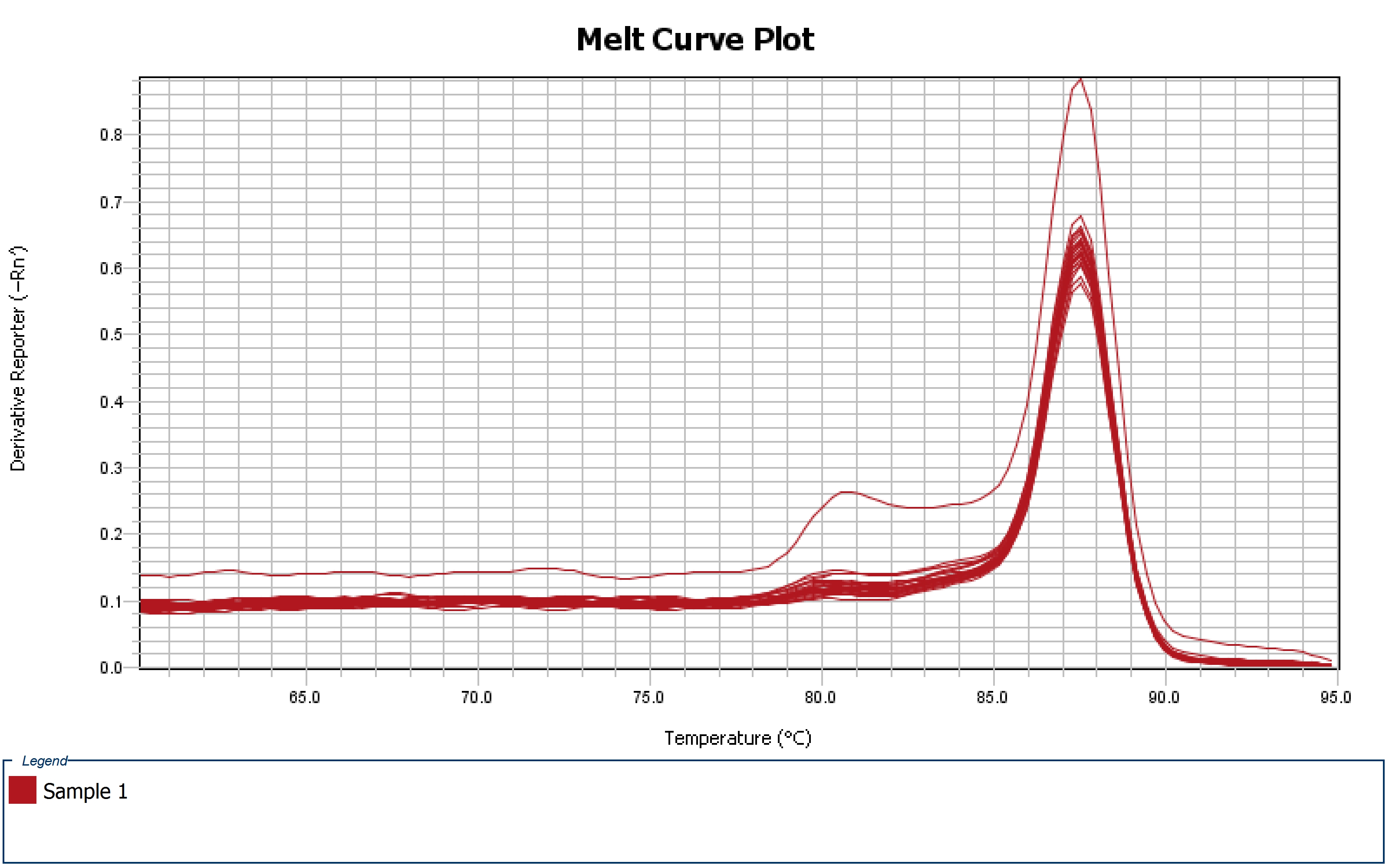

Supplement: Supplementary file 1 [file Data_Sheet_1.ZIP › Original data/Fig 9/File 1. Solubilization and amplification curves of CeRNAs/Cyth4 M.jpg]

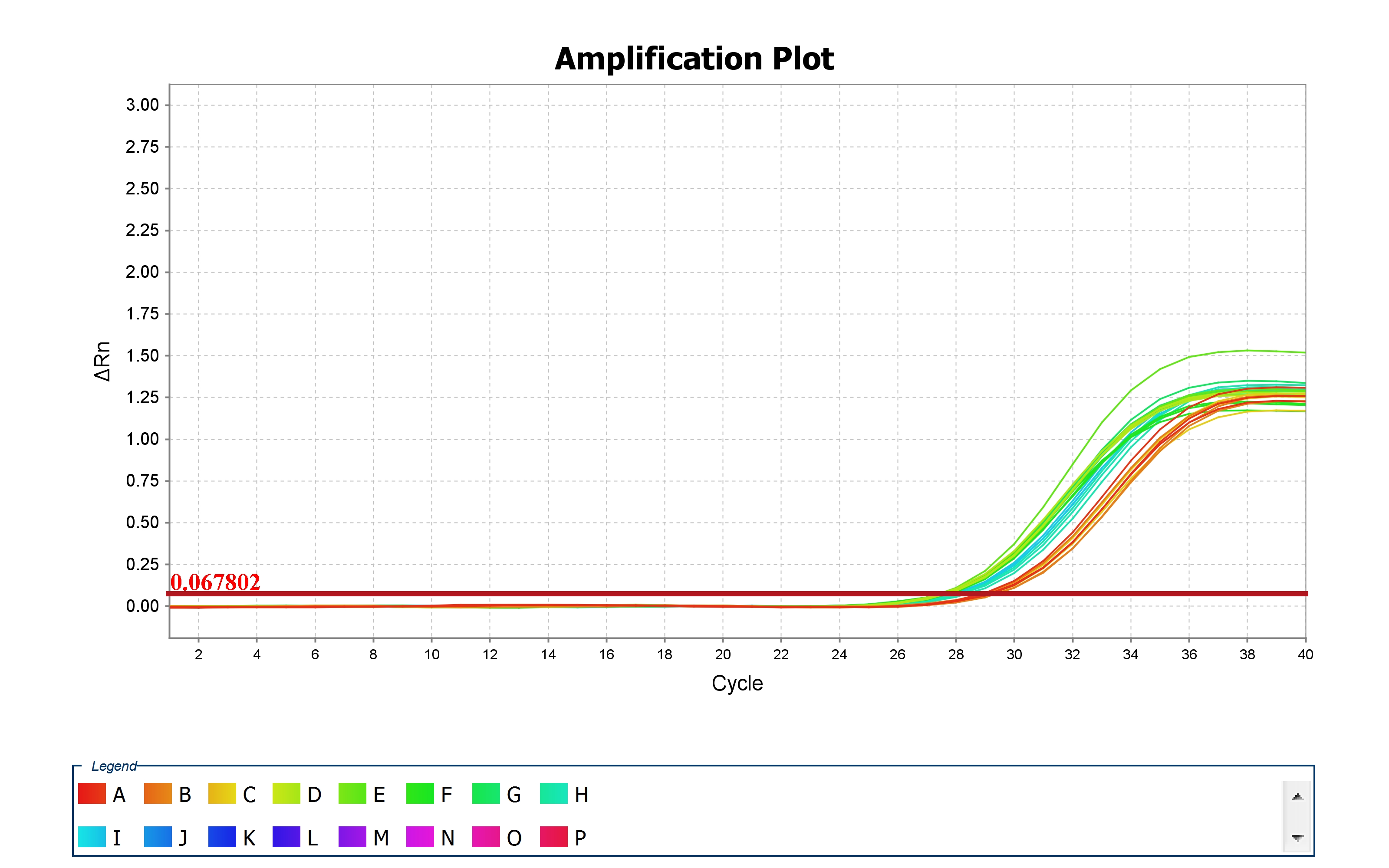

Supplement: Supplementary file 1 [file Data_Sheet_1.ZIP › Original data/Fig 9/File 1. Solubilization and amplification curves of CeRNAs/Cyth4.jpg]

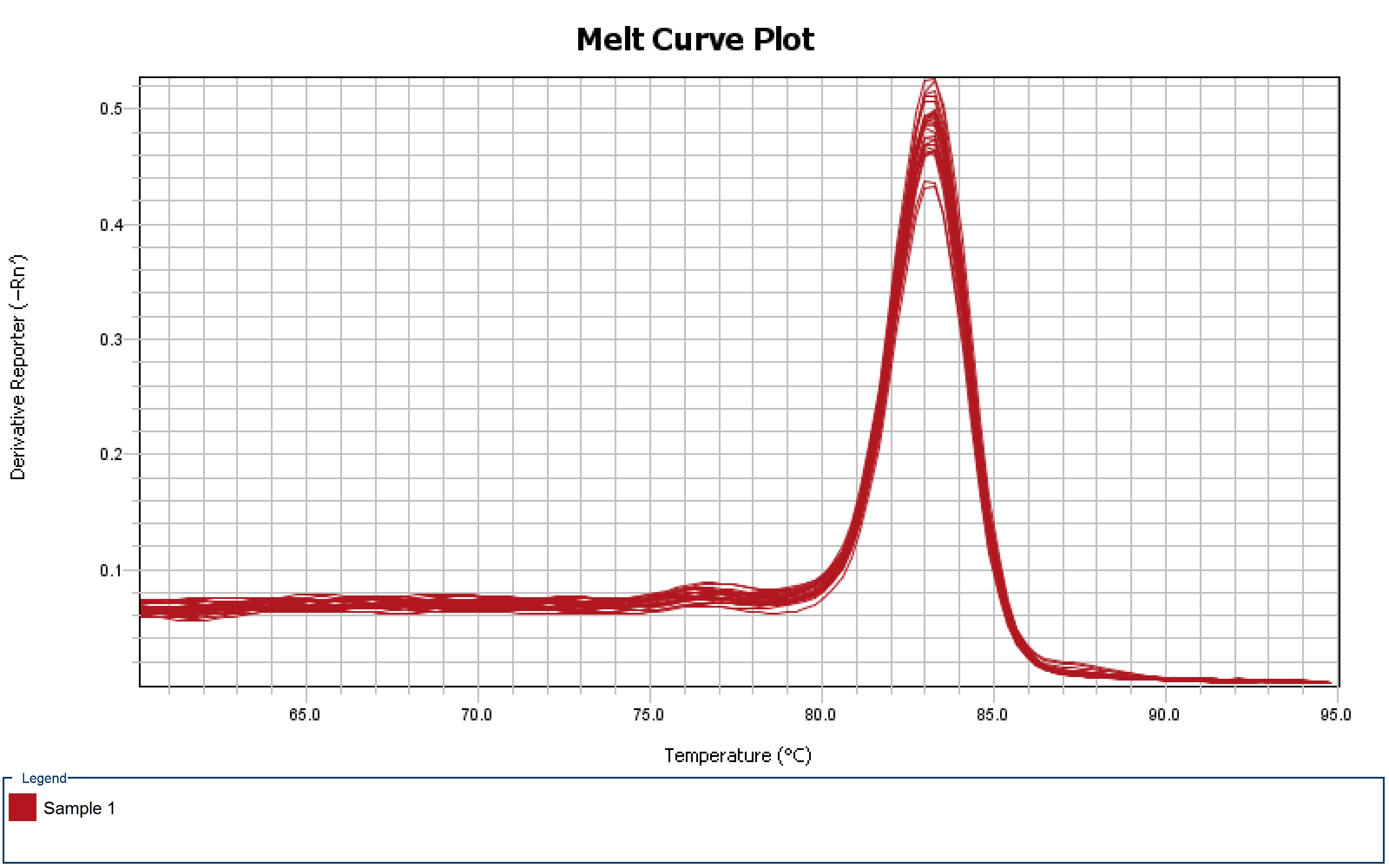

Supplement: Supplementary file 1 [file Data_Sheet_1.ZIP › Original data/Fig 9/File 1. Solubilization and amplification curves of CeRNAs/Fn1 M.jpg]

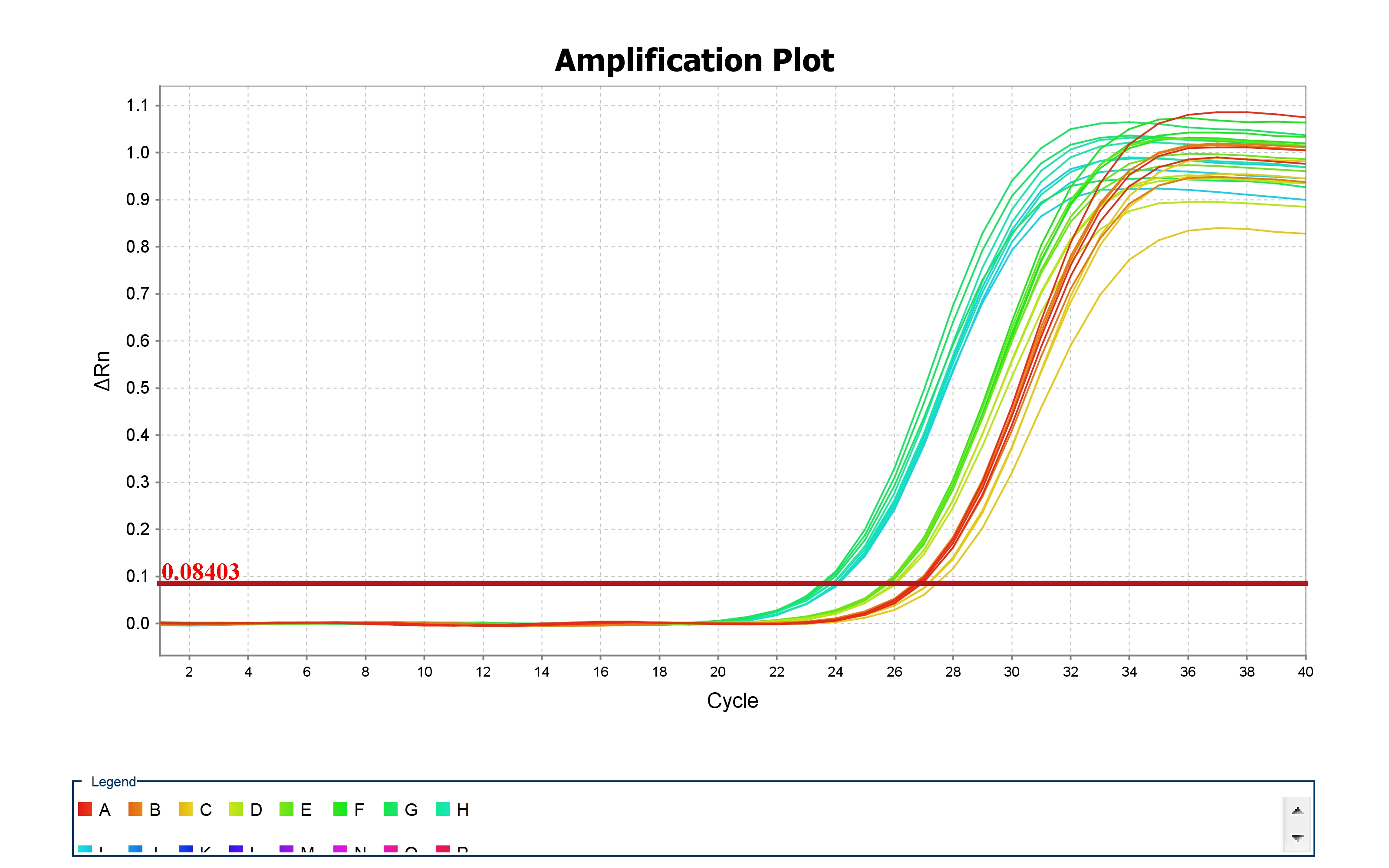

Supplement: Supplementary file 1 [file Data_Sheet_1.ZIP › Original data/Fig 9/File 1. Solubilization and amplification curves of CeRNAs/Fn1.jpg]

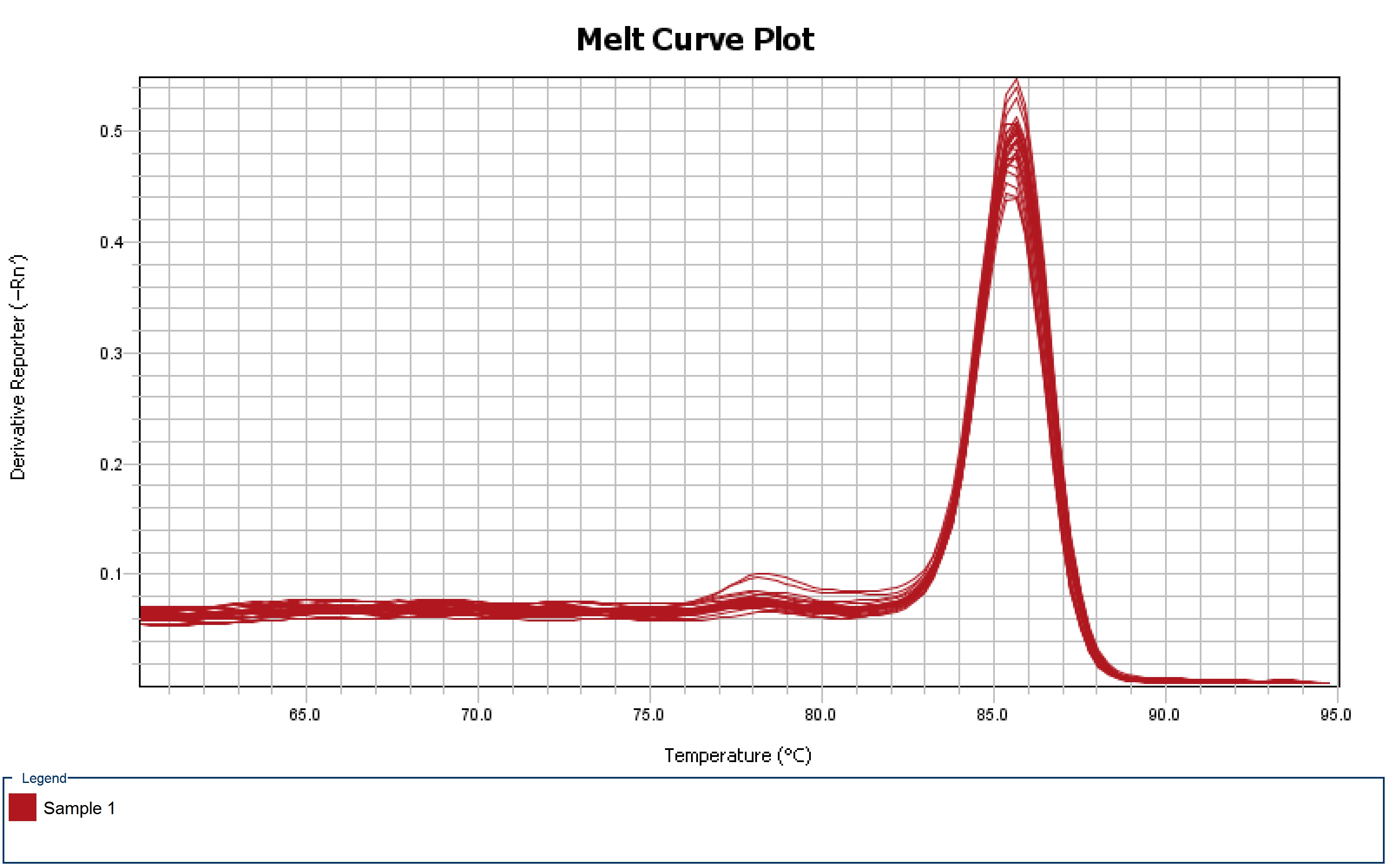

Supplement: Supplementary file 1 [file Data_Sheet_1.ZIP › Original data/Fig 9/File 1. Solubilization and amplification curves of CeRNAs/GAPDH M.jpg]

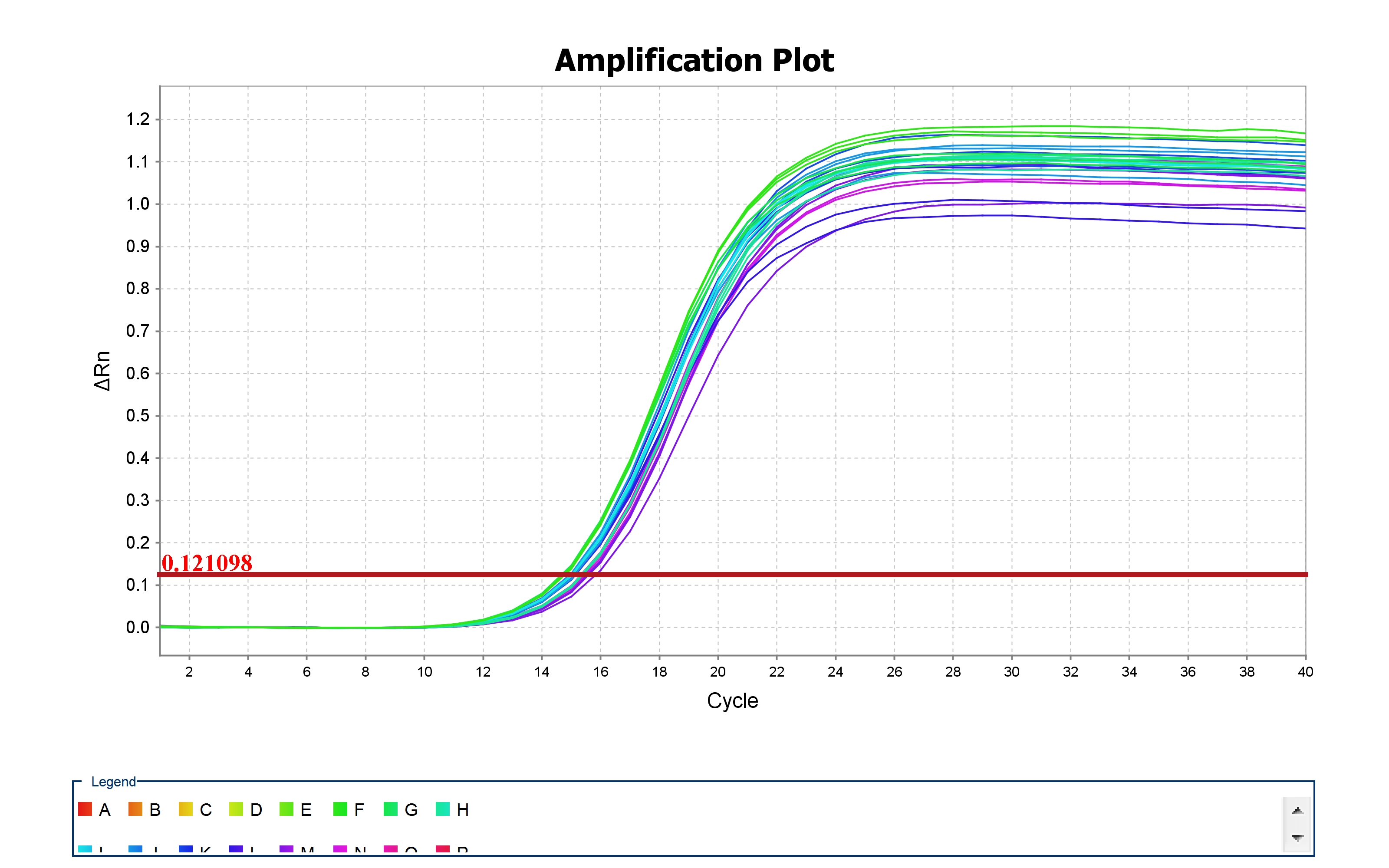

Supplement: Supplementary file 1 [file Data_Sheet_1.ZIP › Original data/Fig 9/File 1. Solubilization and amplification curves of CeRNAs/GAPDH.jpg]

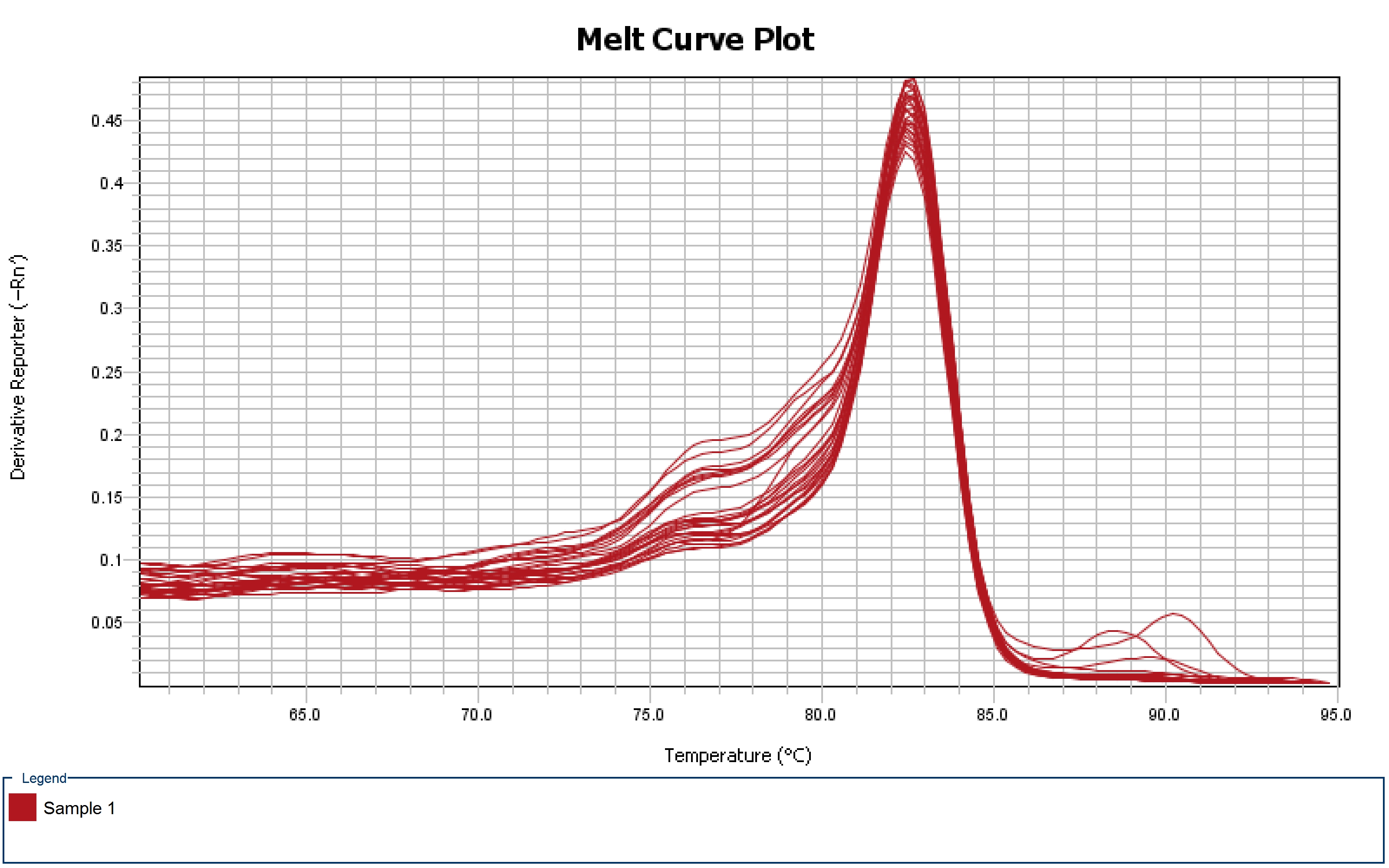

Supplement: Supplementary file 1 [file Data_Sheet_1.ZIP › Original data/Fig 9/File 1. Solubilization and amplification curves of CeRNAs/IKzf1 M.jpg]

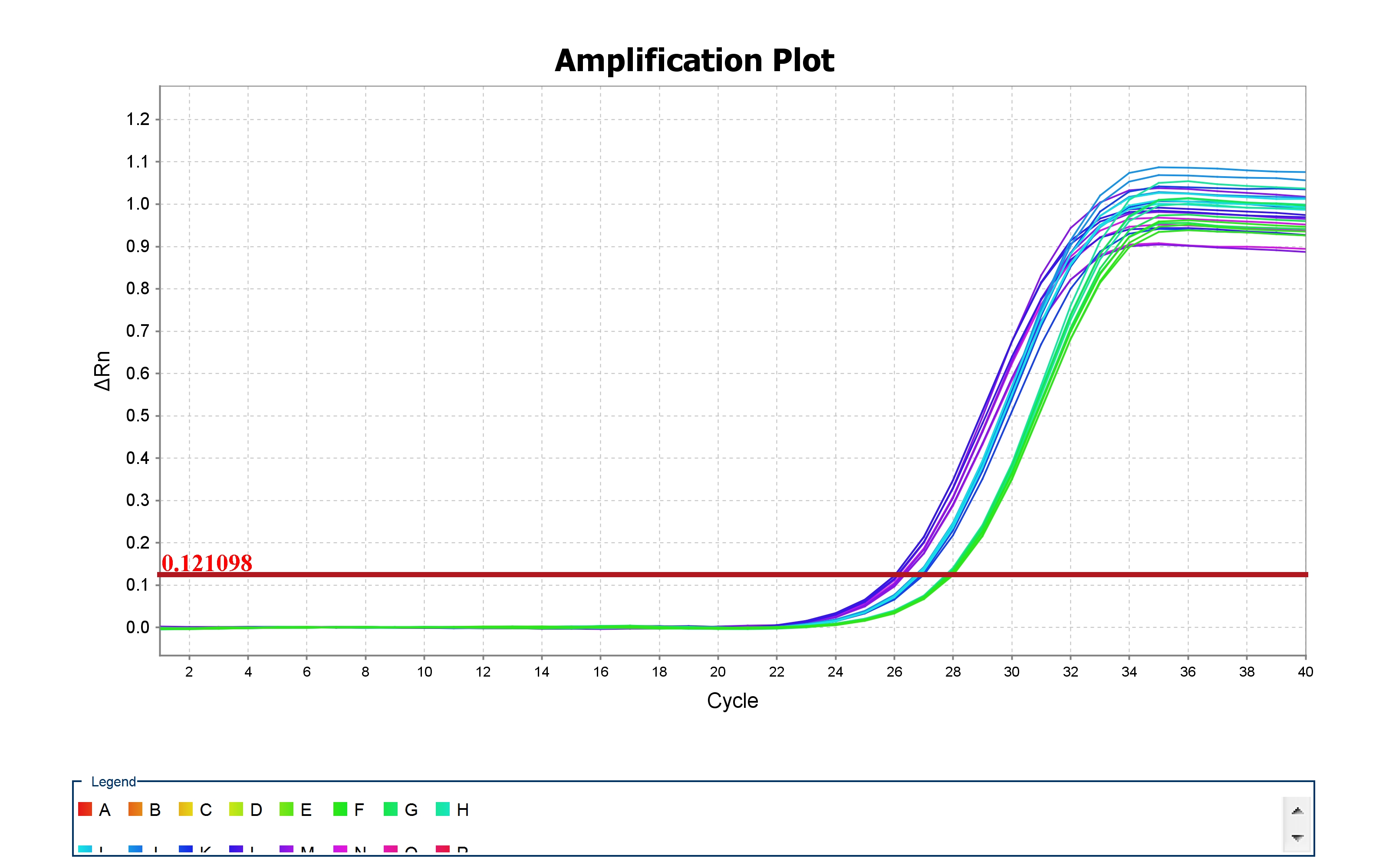

Supplement: Supplementary file 1 [file Data_Sheet_1.ZIP › Original data/Fig 9/File 1. Solubilization and amplification curves of CeRNAs/IKzf1.jpg]

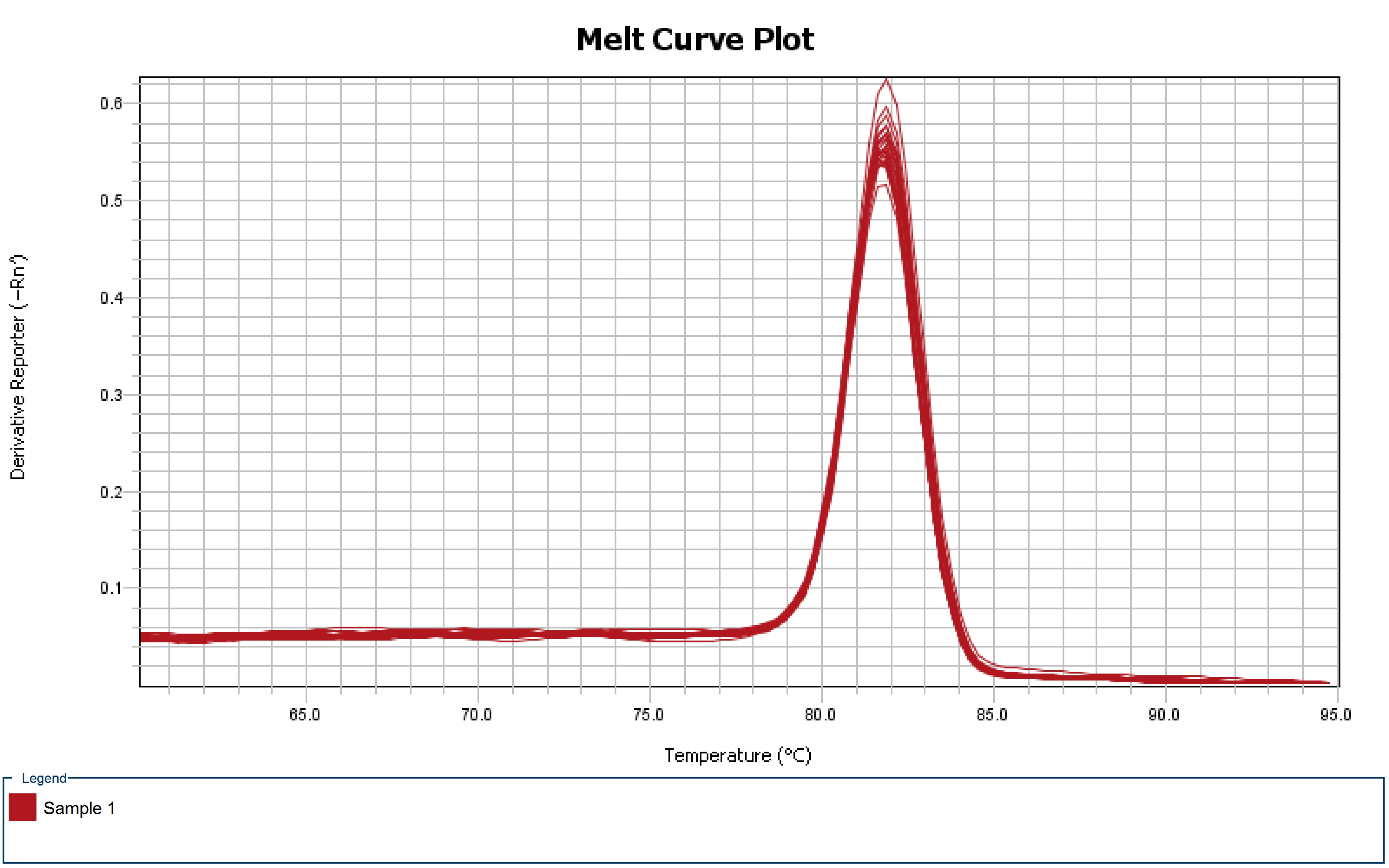

Supplement: Supplementary file 1 [file Data_Sheet_1.ZIP › Original data/Fig 9/File 1. Solubilization and amplification curves of CeRNAs/Loc100911498 M.jpg]

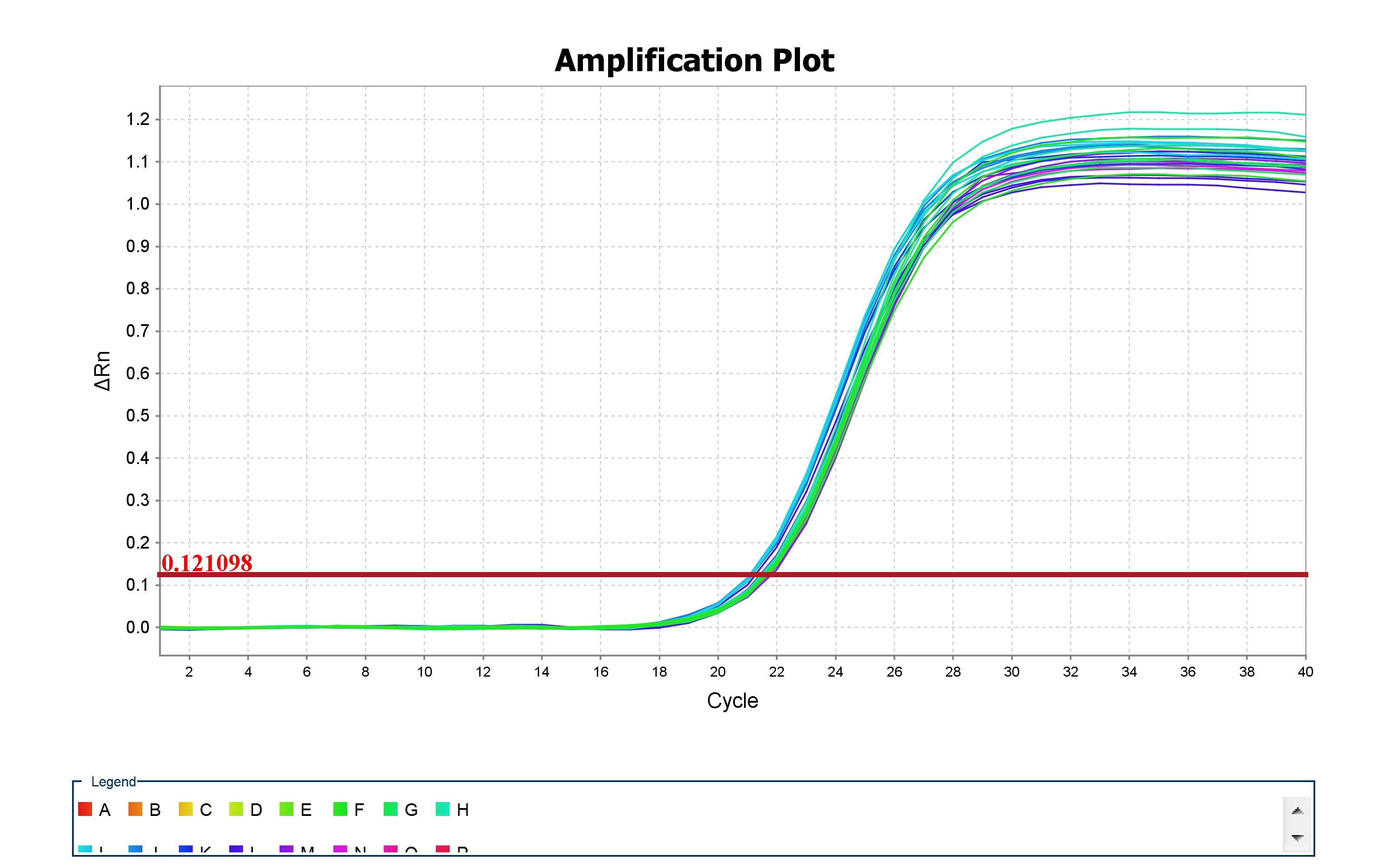

Supplement: Supplementary file 1 [file Data_Sheet_1.ZIP › Original data/Fig 9/File 1. Solubilization and amplification curves of CeRNAs/Loc100911498.jpg]

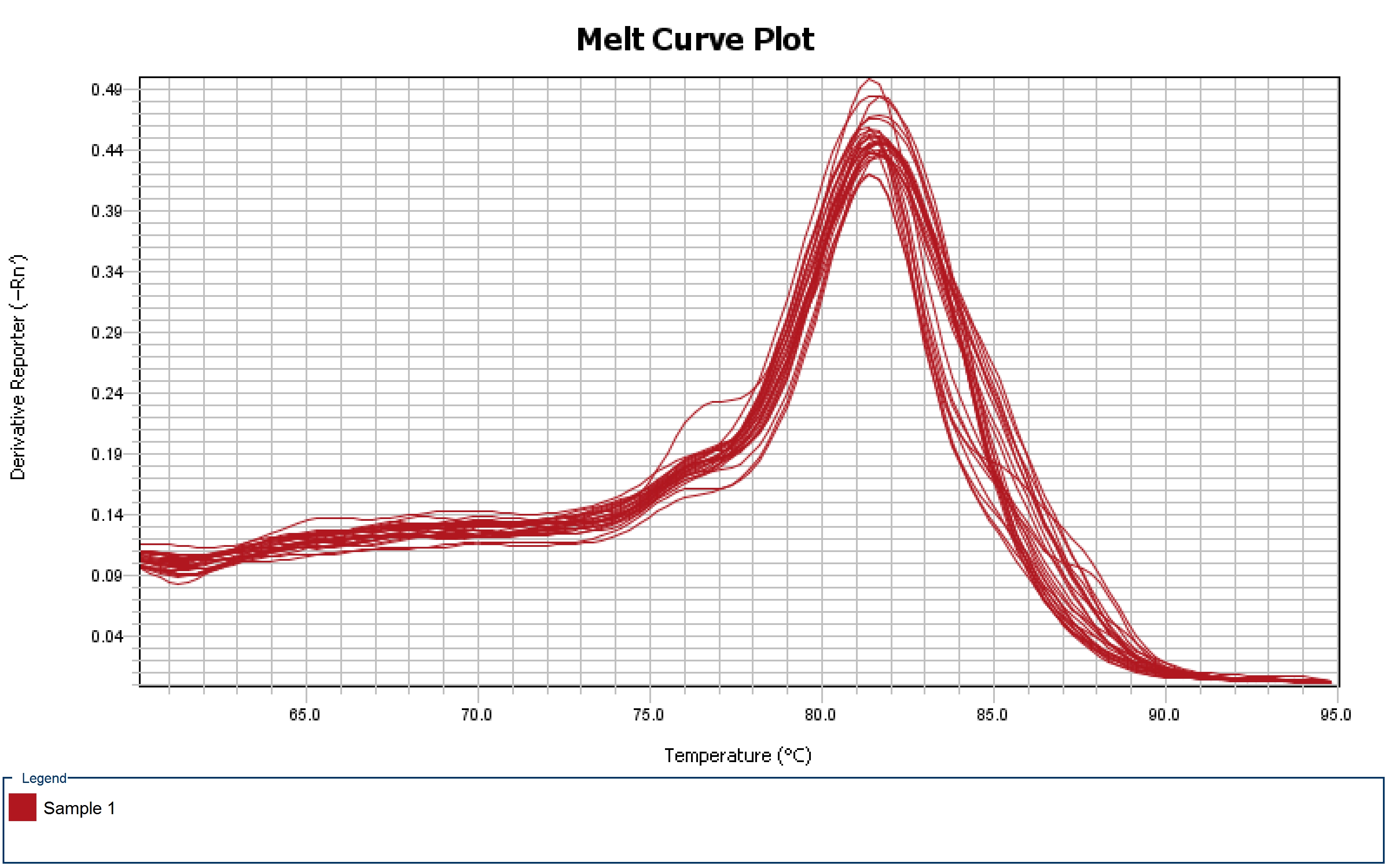

Supplement: Supplementary file 1 [file Data_Sheet_1.ZIP › Original data/Fig 9/File 1. Solubilization and amplification curves of CeRNAs/miR-297 M.jpg]

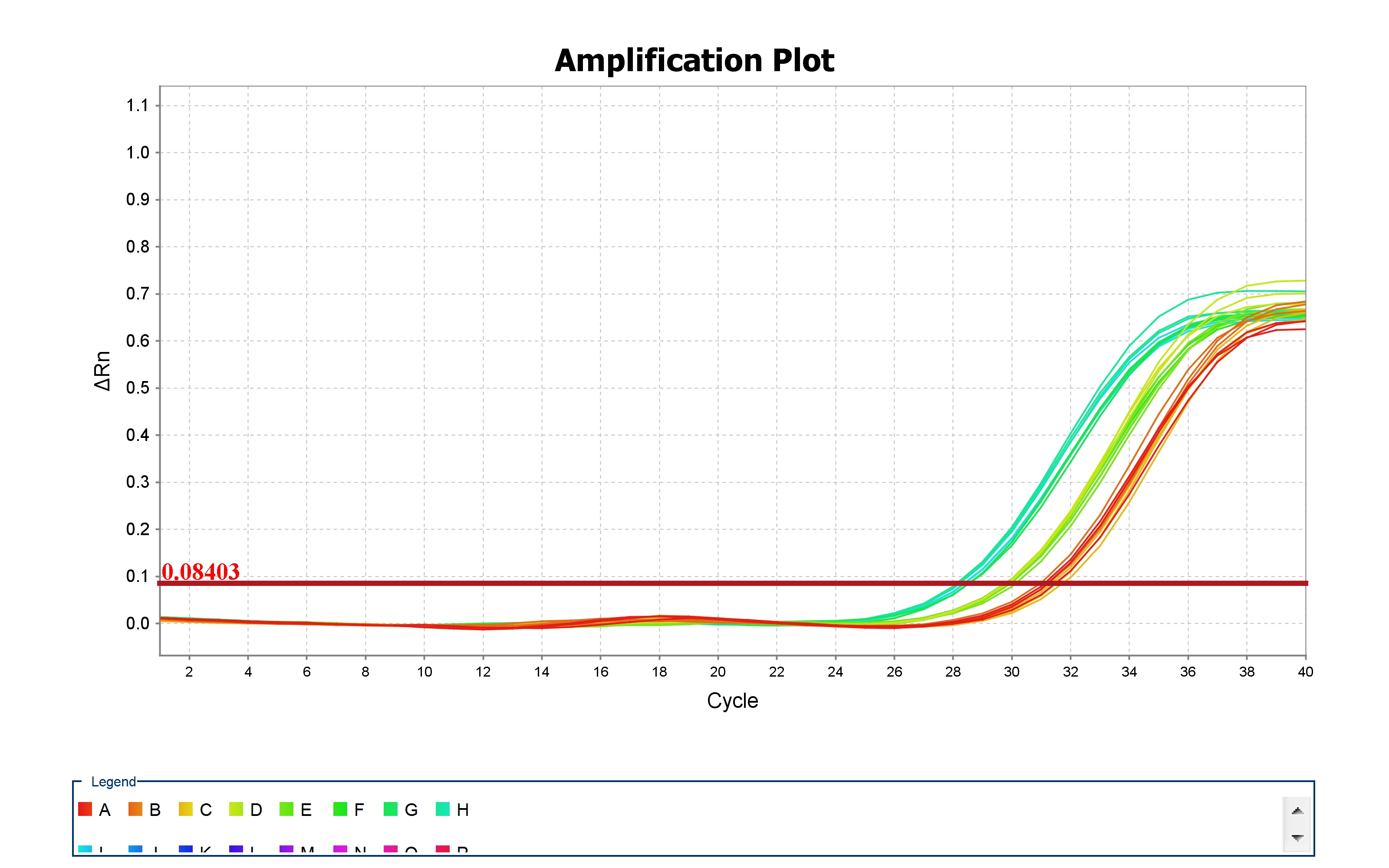

Supplement: Supplementary file 1 [file Data_Sheet_1.ZIP › Original data/Fig 9/File 1. Solubilization and amplification curves of CeRNAs/miR-297.jpg]

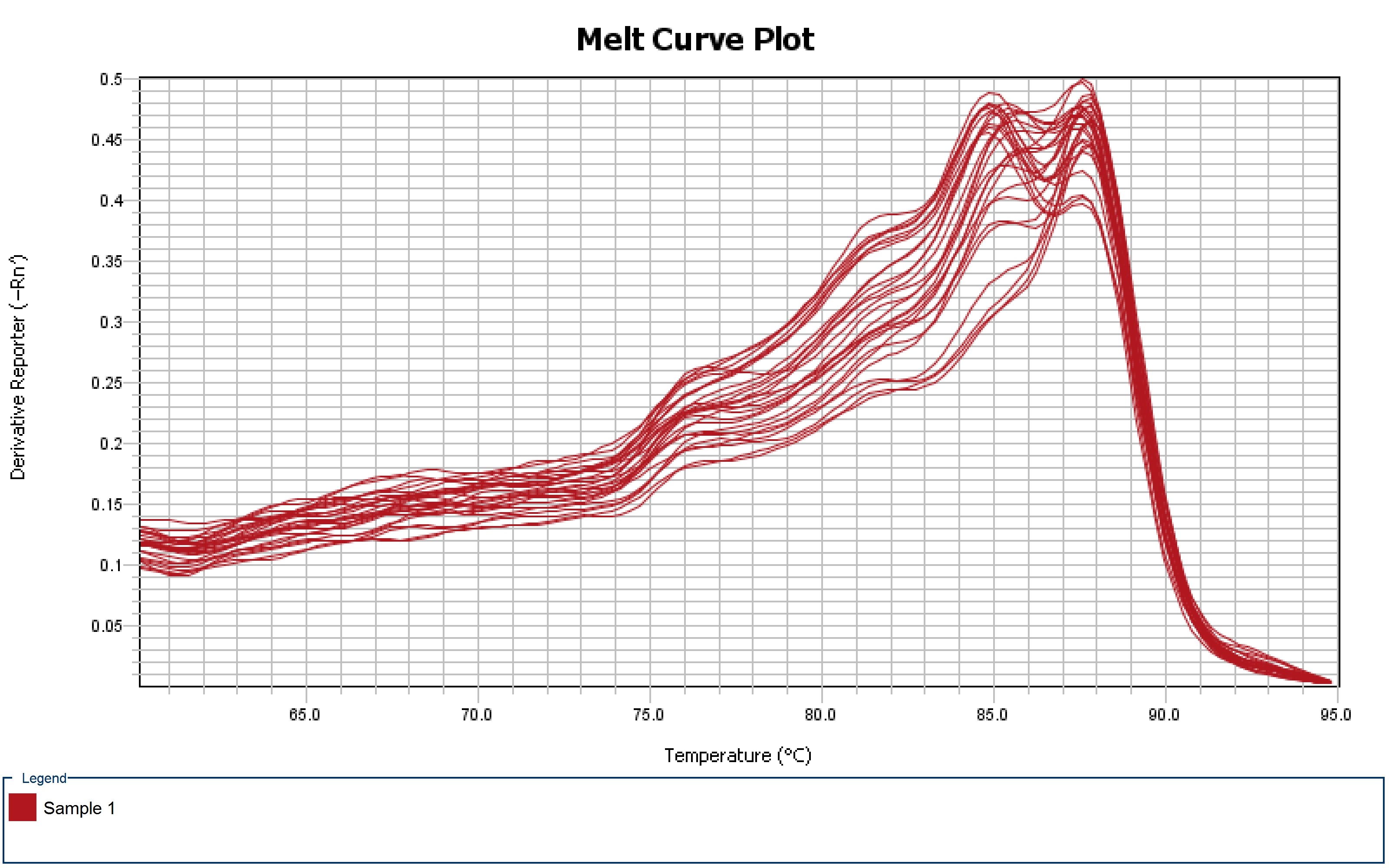

Supplement: Supplementary file 1 [file Data_Sheet_1.ZIP › Original data/Fig 9/File 1. Solubilization and amplification curves of CeRNAs/miR-323-5p M.jpg]

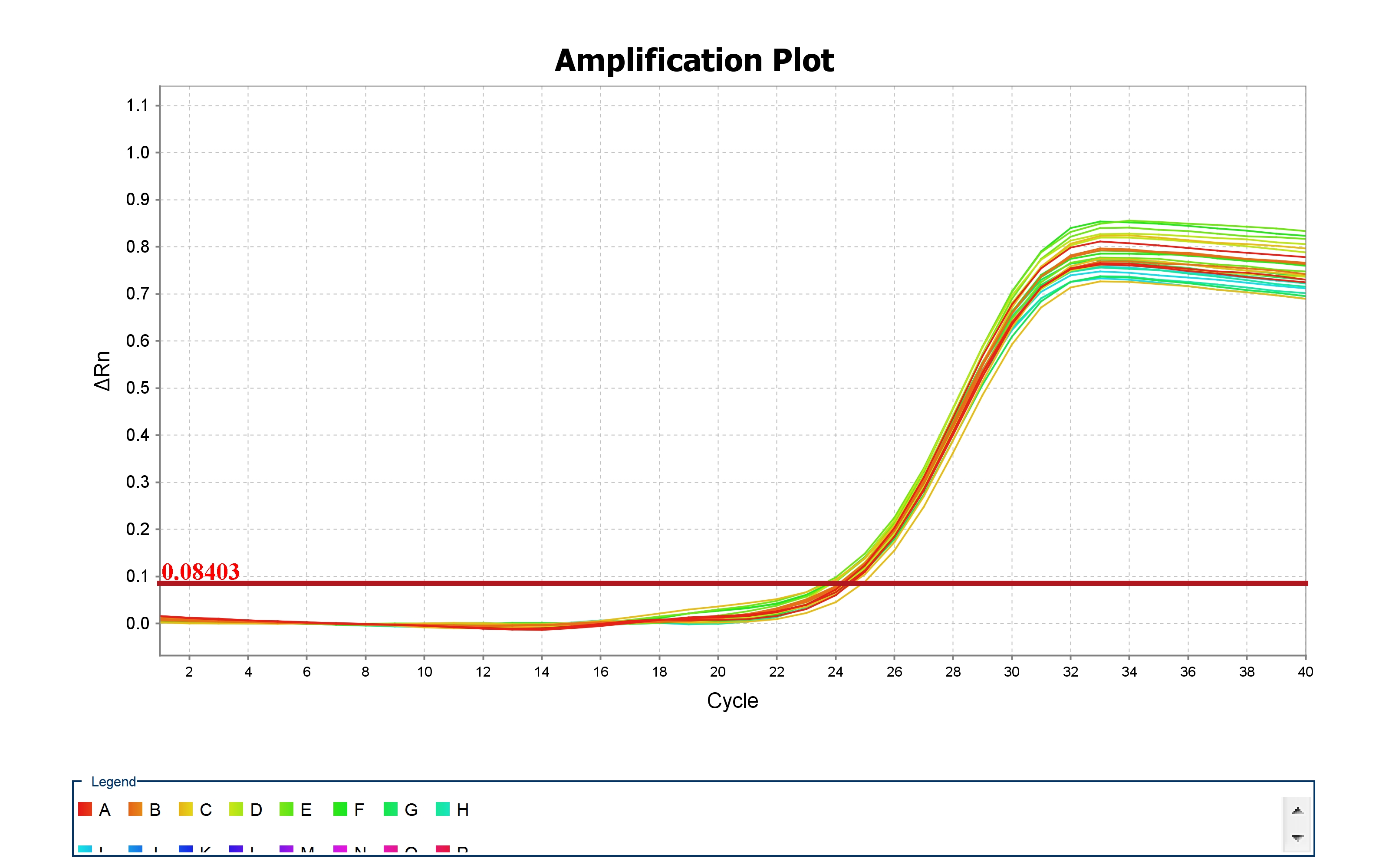

Supplement: Supplementary file 1 [file Data_Sheet_1.ZIP › Original data/Fig 9/File 1. Solubilization and amplification curves of CeRNAs/miR-323-5p.jpg]

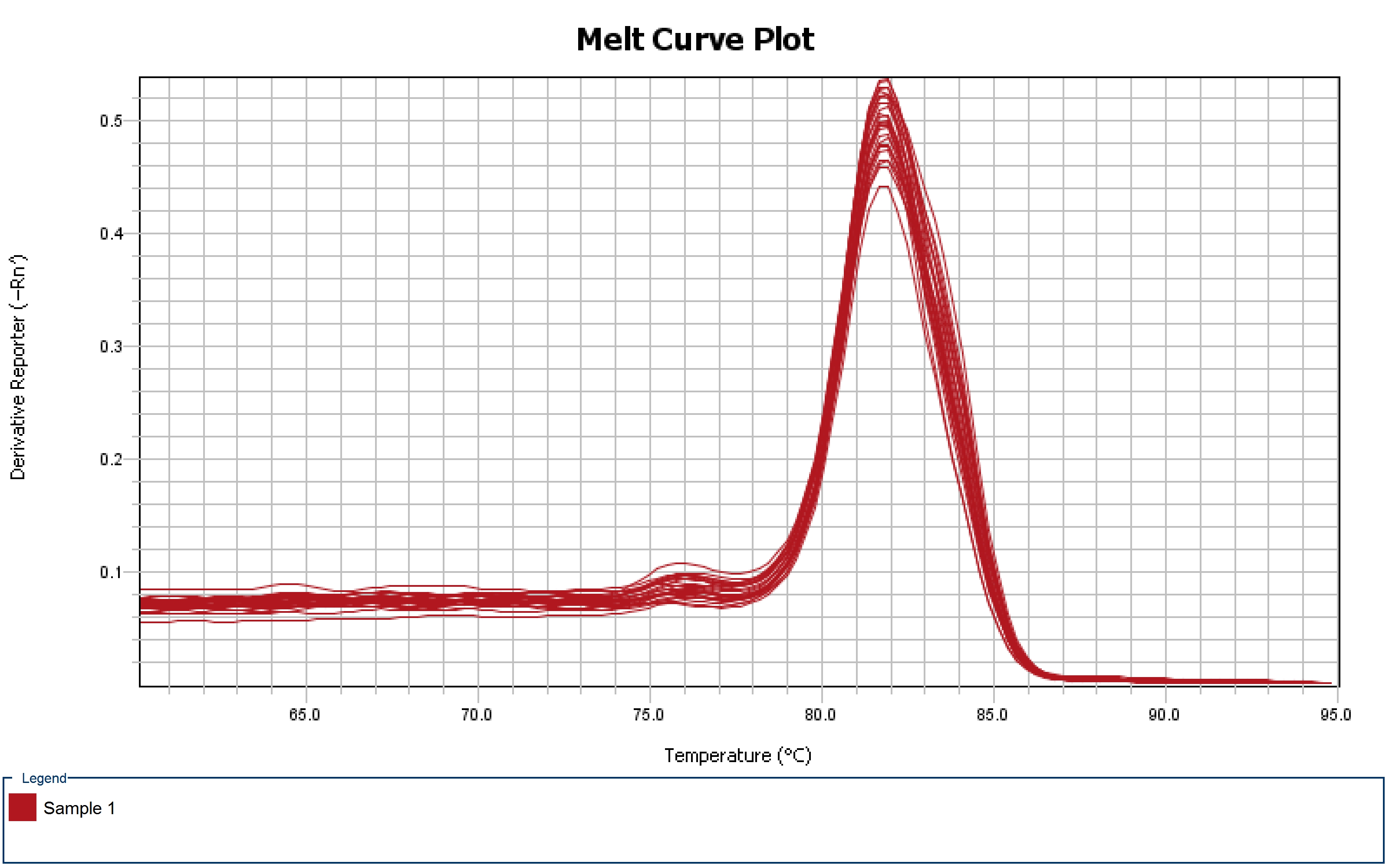

Supplement: Supplementary file 1 [file Data_Sheet_1.ZIP › Original data/Fig 9/File 1. Solubilization and amplification curves of CeRNAs/U6 M.jpg]

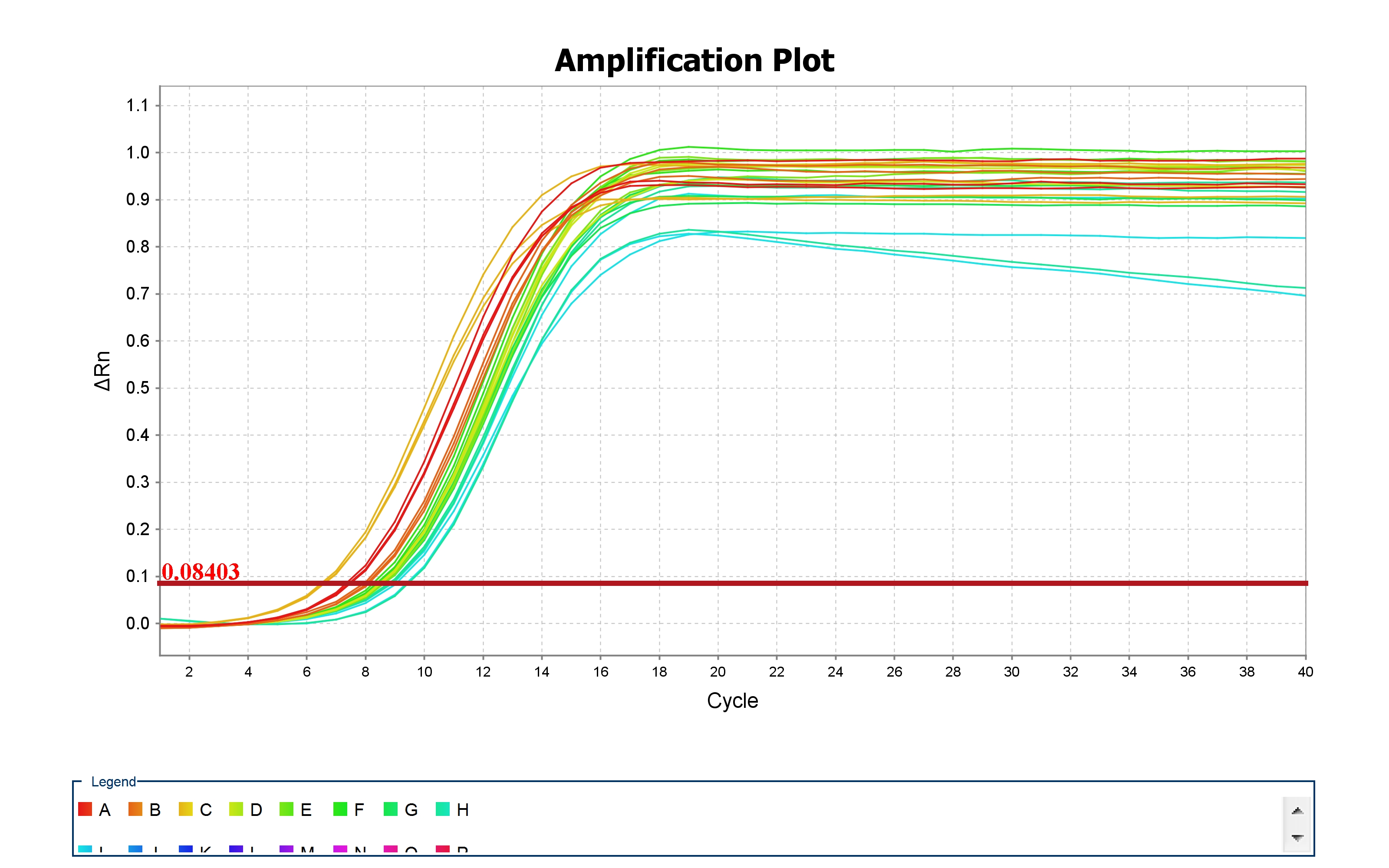

Supplement: Supplementary file 1 [file Data_Sheet_1.ZIP › Original data/Fig 9/File 1. Solubilization and amplification curves of CeRNAs/U6.jpg]

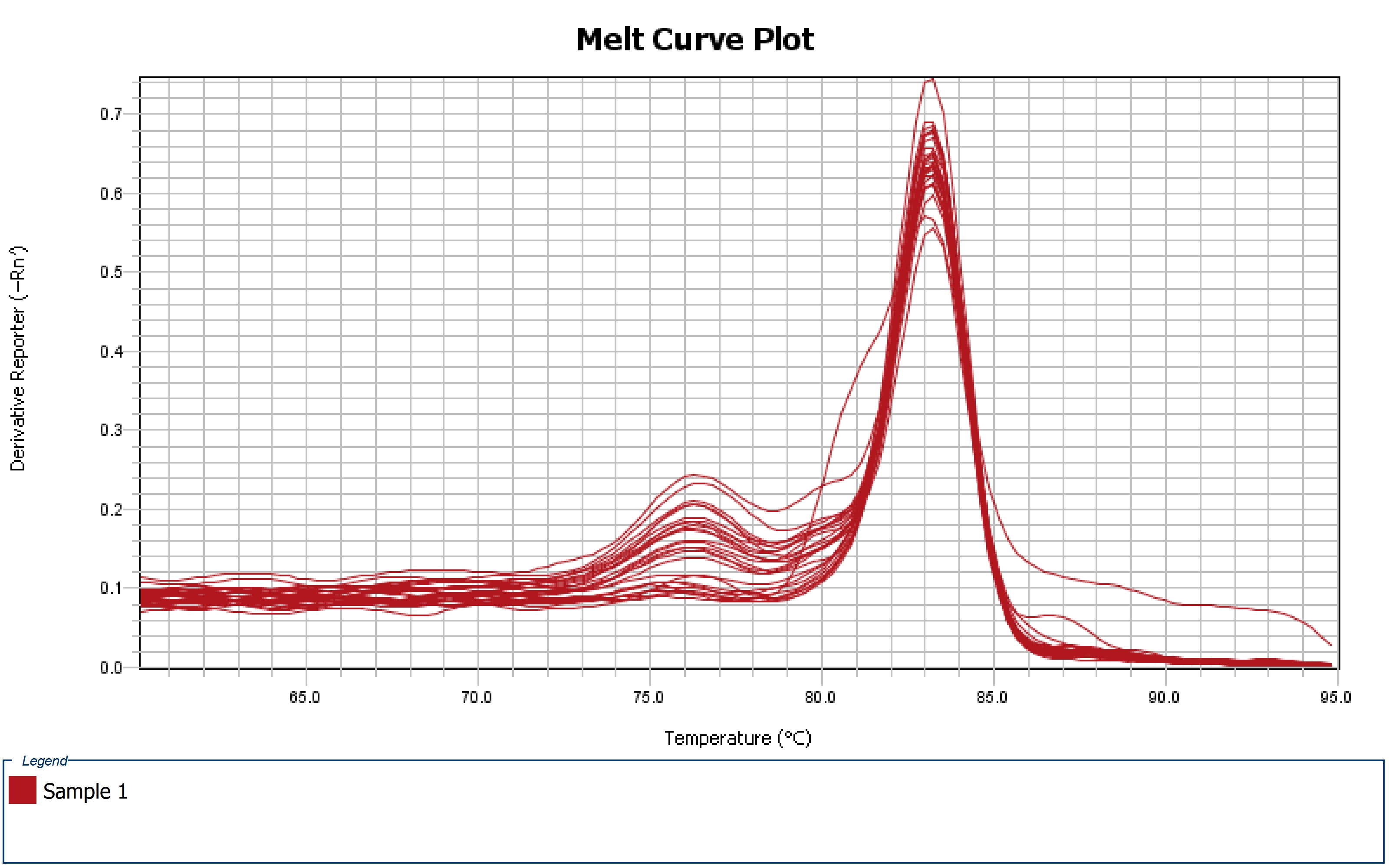

Supplement: Supplementary file 1 [file Data_Sheet_1.ZIP › Original data/Fig 9/File 1. Solubilization and amplification curves of CeRNAs/Wdfy4 M.jpg]

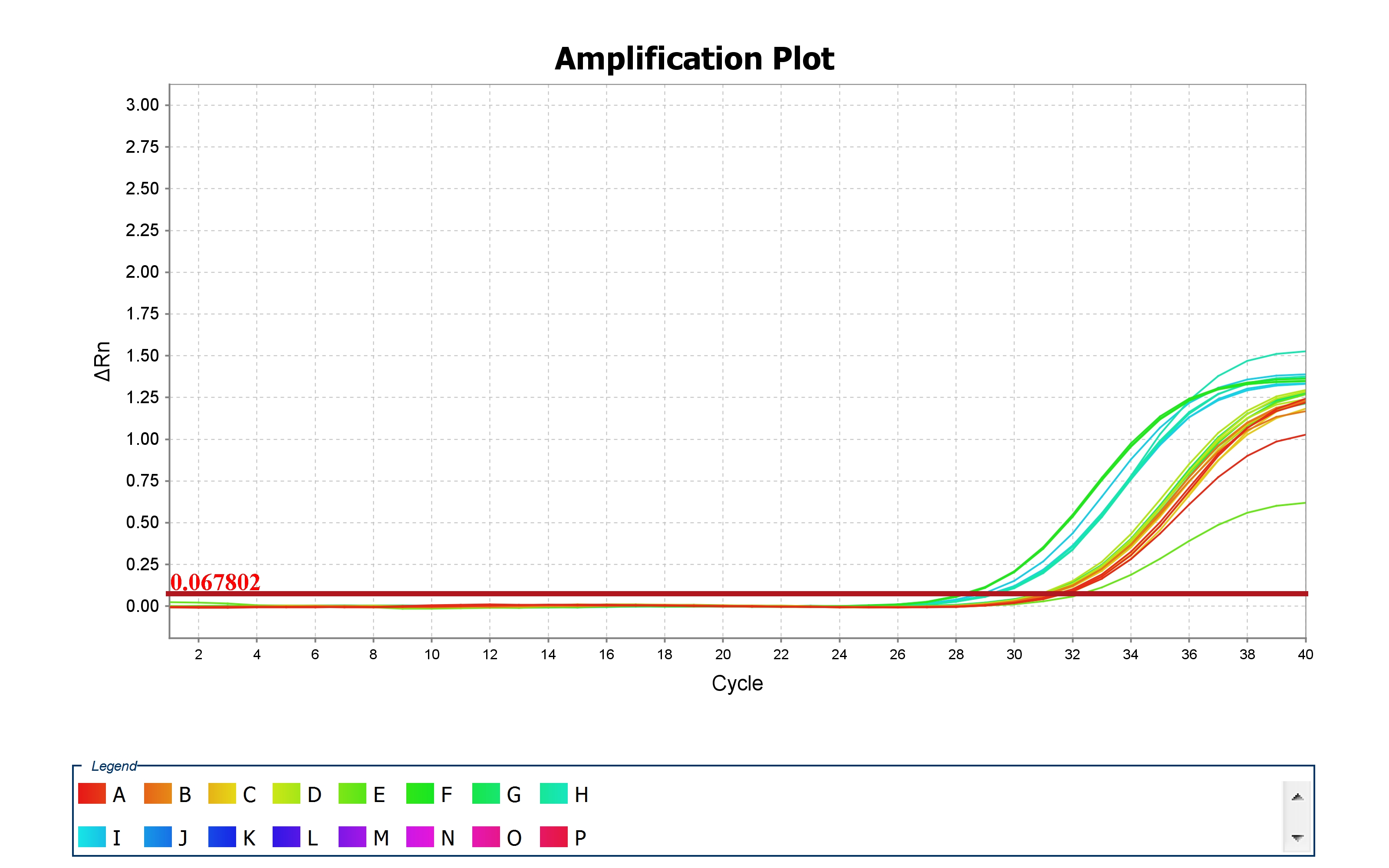

Supplement: Supplementary file 1 [file Data_Sheet_1.ZIP › Original data/Fig 9/File 1. Solubilization and amplification curves of CeRNAs/Wdfy4.jpg]
