# Supplementary material for: Analysis of shared ceRNA networks and related-hub genes in rats with primary and secondary photoreceptor degeneration
Source: Front Neurosci. 2023 Sep 21;17:1259622. doi: 10.3389/fnins.2023.1259622 (PMC10552924; doi:10.3389/fnins.2023.1259622)
Supplement: Supplementary file 2 [file Data_Sheet_2.docx]

Supplementary Material

Analysis of shared ceRNA networks and related-hub genes in rats with primary and secondary photoreceptor degeneration

Jia Liang^1†^, Dong Fang^1†^, Fei Yao^1^, Lu Chen^1^, Zhenhua Zou^1^, Xiangcheng Tang^1^, Lujia Feng^1^, Yijing Zhuang^1^, Ting Xie^1^, Pengxue Wei^1^, Pengfeng Li^1^, Huiyan Zheng^1^, Shaochong Zhang^1^*

*** Correspondence:** shaochongzhang@outlook.com; Tel: +86 182-0099-1688

^†^  These authors contributed to the work equally and share first authorship.

**Supplementary file**

**[Table S1](#_Toc77707431)**[: Detailed primer sequences of ceRNAs 2](#_Toc77707431)

**[Table S2:](#_Toc77707432)** [Share DElncRNAs and interacting DEmiRNAs 2](#_Toc77707432)

**[Table S3](#_Toc77707433)**[: Share DEmiRNAs and interacting DEmRNAs 3](#_Toc77707433)

**[Table S4](#_Toc77707434)**[: The top 5 items in three parts of GO functional enrichment analysis and 11 KEGG pathway of the shared DEGs. 4](#_Toc77707434)

**[Table S5:](#_Toc77707435)** [Detailed information of the top 8 hub genes and corresponding degree score in photoreceptor degeneration 5](#_Toc77707435)

Table S1 Detailed primer sequences of key ceRNAs

| **Category** | **Term** | **Sequences** |
| --- | --- | --- |
| lncRNA | LOC100911498 | Forward: 5′-TGTTGGGACAGCTTTATCAG-3′  Reverse: 5′- GCAGGTAGCCTTCATTTGG-3′ |
| lncRNA | URS0000B2E4CC | Forward: 5′-GGTGTGCACTGGGAGCATAA-3′  Reverse: 5′-AGAGTCTCGGGGACATAGCA-3′ |
| miRNA | rno-miR-323-5p | Forward: 5′-AGGTGGTCCGTGGCG-3′ |
| miRNA | novel-rno-miR297-3p | Forward: 5′-ATGTATGTGTGCATGTA-3′ |
| Gene | C1qb | Forward: 5′-TTCACCTACCACGCCAGTTC-3′  Reverse: 5′-GCTTCAAGACTACCCCACCC-3′ |
| Gene | C1qa | Forward: 5′-CCGCACAGGTCACTTCATCT-3′  Reverse: 5′-CGGGAGGAGGACACGATAGA-3′ |
| Gene | Fn1 | Forward:5´-GAGAATAAGCTGTACCATCGCAA-3´,  Reverse: 5´-CGACCACATAGGAAGTCCCAG-3´; |
| Gene | Wdfy4 | Forward: 5′-GGAGCCCAAGCCCAGAATTA-3′  Reverse: 5′-CGCACTGACTTCTGGATGCT-3′ |
| Gene | Cyth4 | Forward: 5′-GTCTGGCGTACAACCAAGGA-3′  Reverse: 5′-CCCCTGACTACCTCAGCTCT-3′ |
| Gene | Card11 | Forward: 5′-GCCTGTGGAGCATGAATACC-3′  Reverse: 5′-AGTCATCCATAGCTGGCCCT-3′ |
| Gene | Ikzf1 | Forward: 5′-ACGGATACAGAGAGCAACGC-3′  Reverse: 5′-CATGCGGAGTGATGTGGTTG-3′ |
| Gene | Csf2rb | Forward: 5′-GAGCGAGTGGAGCAATGAGT-3′  Reverse: 5′-ATAGATGCAGCCAAAGCGGA-3′ |
| Gene | GAPDH | Forward: 5′-AGACAGCCGCATCTTCTTGT-3′  Reverse: 5′-TGATGGCAACAATGTCCACT-3′ |

Table S2 Share DElncRNAs and interacting DEmiRNAs

| **DELncRNA** | **localization** | **Log_2_ (MNU / RDY)** | **Log_2_ (RCS / RDY)** | **Q-value (MNU / RDY)** | **Q-value**  **(RCS / RDY)** | **DEmiRNA** |
| --- | --- | --- | --- | --- | --- | --- |
| LOC100911498 | Cytoplasmic | 13.15585814 | 13.05147984 | 6.07E-256 | 5.71E-263 | rno-miR-3556a, rno-miR-425-3p, novel-rno-miR297-3p |
| URS0000B2E4CC | Cytoplasmic | 1.18183183 | 1.080097934 | 0.004000292 | 0.046982582 | rno-miR-323-5p |
| URS0000B28F2F | Cytoplasmic | -0.891618495 | -0.638513822 | 8.23E-04 | 0.058326328 | rno-miR-181d-5p |
| URS0000B349C2 | Nuclear | -0.376041019 | -0.68746618 | 0.223518507 | 0.003793066 | novel-rno-miR-57-3p |

RDY, Retinal dystrophy rats; MNU, N-methyl-N-nitrosourea; RCS, Royal College of Surgeons rats; DElncRNA, differentially expressed long noncoding RNA; DEmiRNA, differentially expressed microRNA

Table S3 Share DEmiRNAs and interacting DEmRNAs

| **DEmiRNA** | **Log_2_ (MNU / RDY)** | **Log_2_ (RCS / RDY)** | **Q-value**  **(MNU / RDY)** | **Q-value**  **(RCS / RDY)** | **DEmRNA** |
| --- | --- | --- | --- | --- | --- |
| rno-miR-3556a | -0.637685955 | -0.875473717 | 3.29E-04 | 5.64E-07 | Prtg, RGD1564409, Steap4, Adamts1 |
| rno-miR-425-3p | -0.645613566 | -0.672493157 | 0.010673722 | 0.009377389 | Tgm2, Cmklr1, Hspb1, Pld4, Lyn, Lrg1 |
| rno-miR-323-5p | -0.823942432 | -0.707853413 | 0.034638293 | 0.006847613 | Tgm2, Cmklr1, Rps6ka2, Gfap, Hspb, Aqp5, Ctsz, Icam1, Bace2, Col4a1, Cmtm3, Tmem176a, Pik3cg, Nid2, Elf4, Ikzf1, C1r, Hspg2, Wnt7b, Prtg, Sp140, Tlr13, Arhgdib, Pld4, Eng, Wdfy4, Itgax, Runx1, Fat2, Adamts4, Apcdd1, Lyn, Ddx60, Upp1, Serpinh1, Bmp2, Igtp, Parp14, Col4a2, Tubb6, Togaram2, Mall, Sp100, Itpripl1, Rsad2, Col3a1 |
| rno-miR-181d-5p | 1.005575333 | 0.782650749 | 1.97E-11 | 2.11E-04 | Tmem116, LOC103694537, Mgat4d |
| novel-rno-miR-297-3p | -3.136502374 | -2.792410129 | 3.18E-04 | 6.89E-05 | Tgm2, Cmklr1, Rps6ka2, Gfap, Aqp5, Ctsz, Icam1, Bace2, Col4a1, Cmtm3, Tmem176a, Pik3cg, Nid2, Elf4, Ikzf1, C1r, Hspg2, Wnt7b, Sp140, Tlr13, Arhgdib, Eng, Wdfy4, Itgax, Runx1, Fat2, Adamts4, Apcdd1, Card11, LOC103690031, Kremen1, F2rl1, Ccr5, P2ry6, Lcn2, Csf2rb, Gbp2, Osgin1, Slco2a1, Serpine1, Ggta1, Ptpn7, RT1-N3, Vav1, F2r, Tnfrsf1a, Fn1, Il1r1, Cebpd, Hcls1, Pik3ap1, Cd180, Rhoc, Neurog2, C1qb, Irf5, C1qa, Parp9, Axl, Trim21, Itgal, Itgb2, Sec24d, Adamts9, Map3k6, Tal1, Slc11a1, Tmem154, Eva1b, Arhgap9, Rac2, Apold1, Slc8b1, Cyth4, Rab7b, Adcy4, Arpc1b, Inpp5d, Bcl3, Cyba, Mmp2, Thbd, Cd93, Socs3 |

RDY, Retinal dystrophy rats; MNU, N-methyl-N-nitrosourea; RCS, Royal College of Surgeons rats; DEmiRNA, differentially expressed microRNA; DEmRNA, differentially expressed messenger RNA.

Table S4 The top 5 items in three parts of GO functional enrichment analysis and 11 KEGG pathway of the shared DEGs.

| **Category** | **Term** | **Count** | **q value** |
| --- | --- | --- | --- |
| GO: molecular function | GO:0002020 protease binding | 8 | 2.73E-04 |
| GO: molecular function | GO:0030369 ICAM-3 receptor activity | 2 | 0.004408096 |
| GO: molecular function | GO:0005178 integrin binding | 6 | 0.01312118 |
| GO: molecular function | GO:0015057 thrombin-activated receptor activity | 2 | 0.01312118 |
| GO: molecular function | GO:0140031 phosphorylation-dependent protein binding | 2 | 0.020661668 |
| GO: biological process | GO:0030198 extracellular matrix organization | 11 | 6.00E-06 |
| GO: biological process | GO:0007229 integrin-mediated signaling pathway | 8 | 6.13E-05 |
| GO: biological process | GO:0006954 inflammatory response | 11 | 6.85E-04 |
| GO: biological process | GO:0006909 phagocytosis | 6 | 6.85E-04 |
| GO: biological process | GO:0045766 positive regulation of angiogenesis | 8 | 6.85E-04 |
| GO: cellular component | GO:0031012 extracellular matrix | 12 | 5.34E-06 |
| GO: cellular component | GO:0009986 cell surface | 18 | 5.34E-06 |
| GO: cellular component | GO:0005615 extracellular space | 24 | 8.79E-05 |
| GO: cellular component | GO:0005604 basement membrane | 7 | 1.51E-04 |
| GO: cellular component | GO:0005581 collagen trimer | 5 | 5.99E-04 |
| KEGG pathway | rno04610: Complement and coagulation cascades | 8 | 4.85E-05 |
| KEGG pathway | rno05146: Amoebiasis | 8 | 5.62E-05 |
| KEGG pathway | rno04670: Leukocyte transendothelial migration | 7 | 0.001079887 |
| KEGG pathway | rno04662: B cell receptor signaling pathway | 6 | 0.001079887 |
| KEGG pathway | rno05418: Fluid shear stress and atherosclerosis | 7 | 0.003608412 |
| KEGG pathway | rno05166:Human T-cell leukemia virus 1 infection | 7 | 0.0257145 |
| KEGG pathway | rno04145:Phagosome | 6 | 0.0257145 |
| KEGG pathway | rno04611:Platelet activation | 5 | 0.0257145 |
| KEGG pathway | rno05135:Yersinia infection | 5 | 0.0308657 |
| KEGG pathway | rno04010:MAPK signaling pathway | 7 | 0.04757431 |
| KEGG pathway | rno05132:Salmonella infection | 6 | 0.07043737 |

Table S5 Detailed information of the top 8 hub genes and corresponding degree score in photoreceptor degeneration

| **Rank** | **Gene Symbol** | **Degree score** |  |
| --- | --- | --- | --- |
| 1 | C1qb | 8 |  |
| 1 | C1qa | 8 |  |
| 3 | Cyth4 | 7 |  |
| 3 | Wdfy4 | 7 |  |
| 3 | Fn1 | 7 |  |
| 3 | Ikzf1 | 7 |  |
| 7 | Card11 | 6 |  |
| 7 | Csf2rb | 6 |  |
